# Synthesis, biological evaluation and theoretical studies of (*E*)-1-(4-sulfamoyl-phenylethyl)-3-arylidene-5-aryl-1H-pyrrol-2(3H)-ones as human carbonic anhydrase inhibitors

**DOI:** 10.1080/14756366.2023.2189126

**Published:** 2023-03-23

**Authors:** Farhat Ramzan, Syed Ayaz Nabi, Mehak Saba Lone, Alessandro Bonardi, Aabid Hamid, Sameena Bano, Kalicharan Sharma, Syed Shafi, Mohammed Samim, Kalim Javed, Claudiu T. Supran

**Affiliations:** a Department of Chemistry, School of Chemical and Life Sciences, New Delhi, India; b Neurofarba Department, Section of Pharmaceutical Chemistry, Università degli Studidi Firenze, Firenze, Italy; c Theoretical Chemistry Section, Chemistry Division, Bhabha Atomic Research Centre, Mumbai, Maharastra, India; d Department of Computer Science and Engineering, School of Engineering Sciences and Technology, New Delhi, India; e Department of Pharmaceutical Chemistry, Delhi Pharmaceutical Sciences and Research University Pushpvihar, New Delhi, India

**Keywords:** Human carbonic anhydrase, benzenesulphonamide, pyrrolone, anticancer drugs, enzyme inhibitors

## Abstract

A series of 20 newly designed (*E*)-1-(4-sulphamoylphenylethyl)-3-arylidene-5-aryl-1H-pyrrol-2(3H)-ones was synthesised and assessed as carbonic anhydrase (CA, EC 4.2.1.1) inhibitors towards four human isoforms of pharmaceutical interest, that is, hCA I, II, IX and XII. The compounds displayed low to high nanomolar potency against all the isoforms. Introducing strong electron withdrawing groups at the *para* position of the arylidene ring increased the binding affinity to the enzyme. All compounds showed acceptable pharmacokinetic range and physicochemical characteristics as determined by computational ADMET analysis. Density Functional Theory (DFT) calculations of **3n** were carried to gain understanding on the stability of the *E* and *Z* isomers. The energy values clearly indicate the stability of *E* isomer over *Z* isomer by −8.2 kJ mol^−1^. Our findings indicate that these molecules are useful as leads for discovering new CA inhibitors.

## Graphical Abstract

**Figure:**
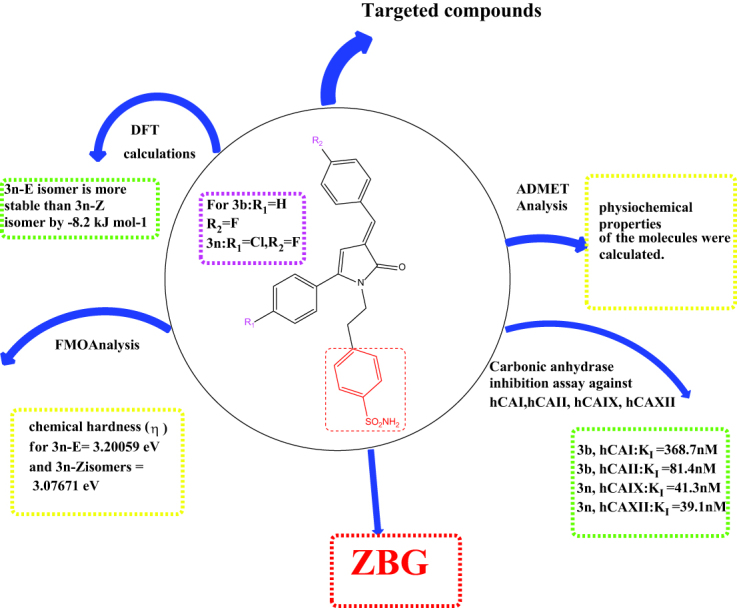

## Introduction

Carbonic anhydrases (EC 4.2.1.1; CA) are a superfamily of ubiquitous metalloenzymes, which play a vital role in several physiological processes in organisms belonging to all life kingdoms, by catalysing the reversible hydration of carbon dioxide into bicarbonate and a proton (CO_2_ +H_2_O ⇋ HCO_3_^-^ + H^+^)^1^. Among the eight unrelated gene families of CAs (α, β, γ, δ, η, ζ, θ and ι) only the α-class was found in higher vertebrates. In *Homo sapiens,* 15 isoforms of α-CAs (hCAs) were identified, each differing by oligomeric state (most are monomers, with CA VI, IX and XII being dimers), molecular features, subcellular location, distribution in tissues and organs, expression levels, as well as kinetic properties^2^^,^^3^. hCA I, III, VII, and XIII are present in the cytosol, whereas hCA IV, IX, XII, and XIV are membrane-bound, hCA VA and VB are located in the mitochondria, isoform VI is secreted, and CA VIII, X, and XI are acatalytic, cytosolic isoforms. All the catalytically active isoforms are important for maintaining the intracellular and extracellular pH and CO_2_/HCO_3_^-^ pool of cells^4^. CA isoforms play important roles in various metabolic reactions in mammals (*i.e.,* gluconeogenesis, lipogenesis and ureagenesis), electrolyte secretion, pH and CO_2_ homeostasis, respiration, bone resorption and tumourigenicity^5^. However, a variety of pathological conditions in humans have been linked to an altered expression/activity of these isoforms. Besides retinal and brain oedema, the cytosolic hCA I and II isoforms have been connected with glaucoma, altitude sickness and epilepsy^6^. hCA IX and hCA XII have been related to inflammation and tumorigenesis. They control the extracellular and intracellular pH levels favouring the growth, progression and metastases formation of many tumour types. Their overexpression in many human malignancies makes them interesting new targets in the development of anticancer drugs for treating hypoxic tumours^7^. Thus, novel therapeutic alternatives may be obtained by interfering with CA activity in such conditions, bringing it to normal levels either through inhibition or activation^8^.

**Figure 1.**
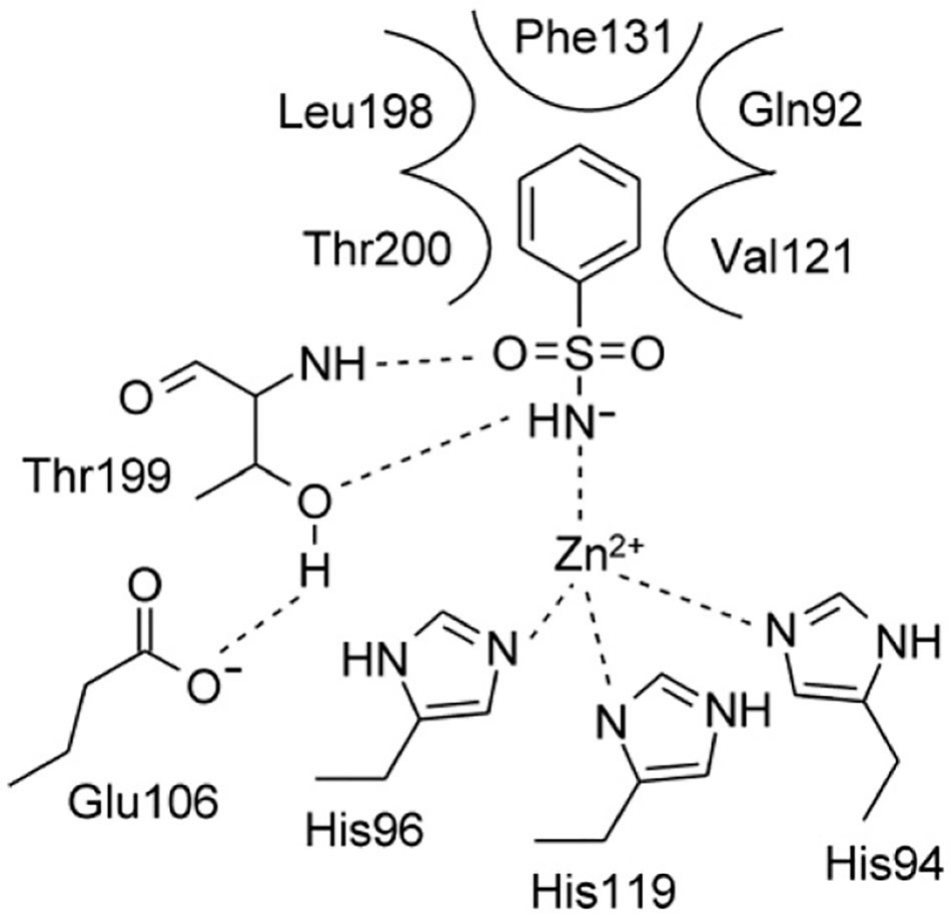
2D-representation of the α-CA-benzenesulphonamide complex displaying the estimated active site region and residues participating in the recognition of the inhibitor component.^11^

The most investigated class of CA inhibitors (CAIs) are the zinc-binders, among which the primary sulphonamides have wide therapeutic applications, such as antiglaucoma agents, diuretics, antidiabetic, and anticancer therapy^9^. In CA-catalysed processes, the inclusion of an aromatic or heteroaromatic scaffold harbouring the sulphonamide group greatly stabilizes the formation of the enzyme-ligand complex^10^. These sulphonamide inhibitors bind to CAs by replacing a water molecule or hydroxide ion coordinated to the Zn^2+^ ion from the active site of enzyme, as in the acetazolamide (AAZ), a drug that has been used in medicines since 1954. The binding mode of the sulphonamides within various CA active sites has been explored by many X-ray crystallographic studies which showed the deprotonated SO_2_NH^-^ moiety coordinated to the zinc ion, and two hydrogen bonds between the NH^-^ and S = O groups with the side-chain OH and backbone NH of the Thr199, respectively a highly conserved amino acid in all α-CAs active sites^11^ (Figure 1).

In a previous study, our team found that a set of benzenesulphonamides connected to the pyridazinone nucleus had an outstanding inhibitory effect against hCAI and II in the nanomolar range^12^. We thus continued and broadened our research in this article to examine the impact of pyrrolone-substituted ethyl benzene sulphonamides on the inhibitory profile of the hCA I, II, IX, and XII isoforms in an effort to find new potent and selective CAIs.

**Figure 2.**
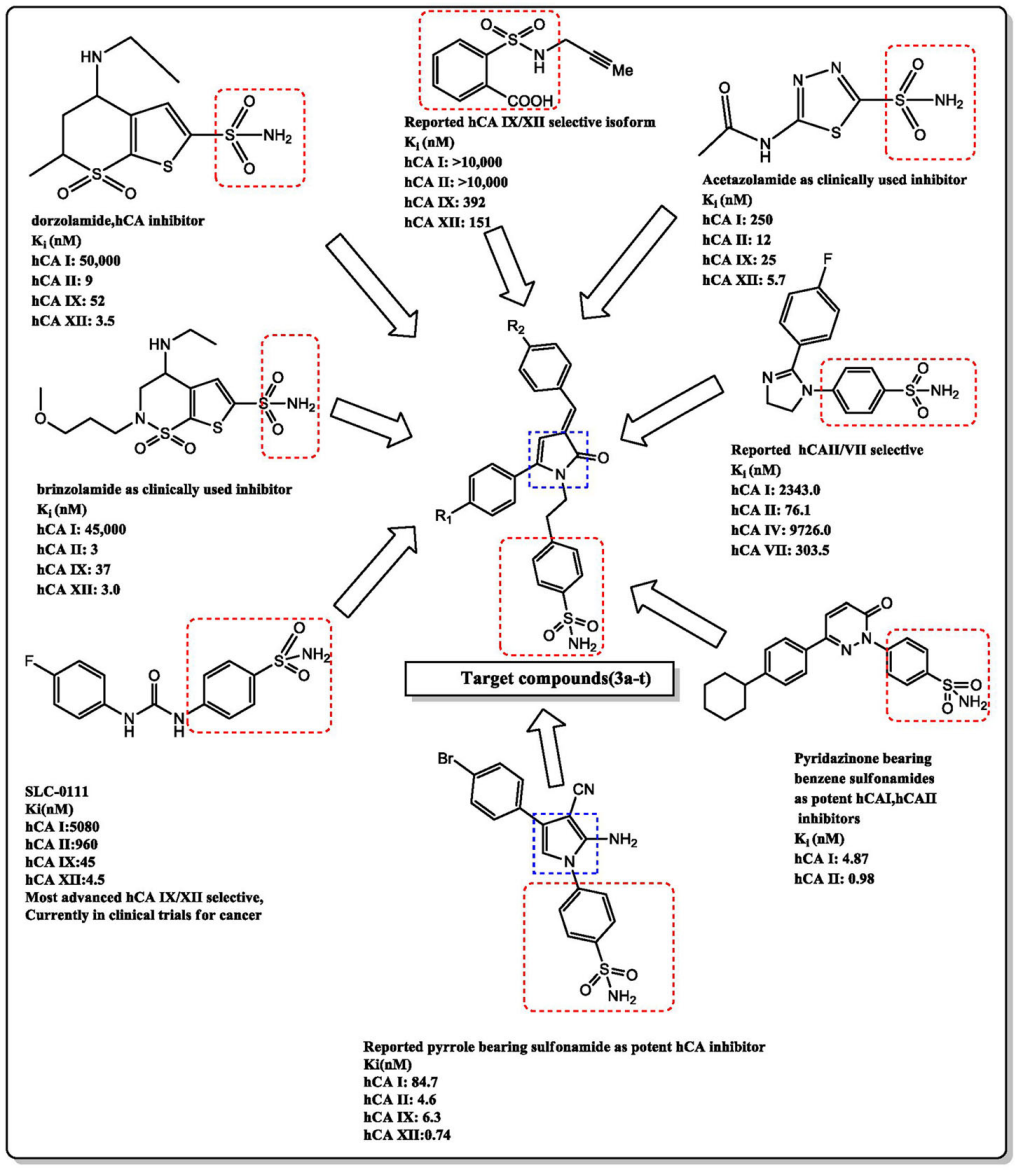
Structure of biologically active sulphonamides as carbonic anhydrase inhibitors and rationally designed template for target compounds.

Due to their significant pharmacological activity, pyrrolones (five-member heterocyclic Δ^3^or Δ^4^-lactams) have garnered a lot of interest in recent years. It has been reported that they have antibacterial^13^, antifungal^14^, anticancer^15^^,^^16^ and anti-inflammatory^17^activities (Figure 2).

## Materials and methods

### Chemistry

Chemicals and starting materials were acquired from Sigma Aldrich, Merck (Germany) and Spectrochem. Open capillary tubes were used for melting point determination and are uncorrected. BIO-RAD FTS-135 spectrophotometer (Waltham, MA, USA) was used to record Fourier Transform Infra-Red (FTIR) by making use of KBr pellets and *v*_max_ values are given in cm^−1^. Bruker spectrospin DPX 400MHZ spectrometer (Fallanden, Switzerland) was used to record ^1^H NMR spectra by using internal standard as tetramethylsilane (TMS) and solvent as CDCl_3_ or (DMSO-d_6_). Coupling constants i.e., J values are expressed in Hertz and chemical shift values are given in δ (ppm) scale. Multiplicities in chemical shift are expressed as s = singlet, d = doublet, t = triplet, and m = multiplet. Bruker spectrospin DPX at 100MHZ was used to record ^13^C spectra. Managed by mass Lynx software (version 4.1), XEVO TQ-S triple quadrupole mass spectrometer (Waters, USA) was used to scan mass spectra (MS) and more intense peak values are specified by *m/z* ratio. TLC plates (silica gel G) were utilised for determining progress of reaction and further visualisation was done by exposing to vapours of iodine. To carry elemental analysis, CHNS Elementar (Vario EL III, Hanau Germany) was used.

#### General procedure for the synthesis of (E)-1–(4-sulfamoylphenylethyl)-3-arylidene-5-aryl-1H-pyrrol-2(3H)-ones (3a-t)

The following two steps were involved in the synthesis of compounds:

1. Synthesis of *γ*-keto-4-sulfamoylphenylethylamide:

Using Friedel Crafts succinoylation, *β*-aroylpropionic acids were synthesised from the appropriate aromatic hydrocarbons. The required 2(3*H*)-furanone derivatives (**1a-t)** were prepared by refluxing the equimolar amount of appropriate *β*-aroylpropionic acids and aromatic aldehydes in acetic anhydride. Then 2(*3H*)-Furanone (3 mmol) and 4-(2-aminoethyl) benzenesulphonamide (4 mmol) were refluxed for 2–5 h in dry benzene. After completion of reaction, the volume of the reaction mixture was reduced to half by removing excess benzene through distillation. The mixture was left at room temperature to give crystals that were filtered off, washed with little volume of dry benzene and then dried to give **2a-t**.

2. Cyclisation of *γ*-keto-4-sulfamoylphenylethylamide:

The crystals of *γ*-keto-4-sulphamoylphenylethylamide (3 mmol) obtained from above experiment were refluxed for 1 h in 6 M hydrochloric acid (20 ml). After cooling the contents, the solid obtained was filtered off, washed with water followed by recrystallisation with methanol to give pure TLC (toluene: ethylacetate: formic acid: 5: 4:1) of compounds (**3a-t)**.

#### (E)-4-(2-(3-benzylidene-2-oxo-5-phenyl-2,3-dihydro-1H-pyrrol-1-yl)ethyl) benzenesulphonamide (3a)

Light yellow crystals, yield = 80%, m.p. 167–168 °C, *R_f_* = 0.74[toluene: ethyl acetate: formic acid::5:4:1]. IRυ_max_ (KBr, incm^−1^): 3743 and 3207 (NH_2_), 1676 (C = O), 1330 and 1150 (SO_2_N). ^1^H NMR (400 MHz, DMSO*-d_6_*, *δ*): 2.72 (2H, *t*, PhCH_2_CH_2_N), 3.89 (2H, *t*, PhCH_2_CH_2_N), 6.39 (1H, *s*, H-4 pyrrolone ring), 7.12 (2H, d, *J* = 8.00 Hz, H-3′, H-5′), 7.25–7.64 (13H_,_ *m*, SO_2_NH_2_, olefinic H and aromatic protons of Ar^1^, Ar^2^ and sulphamoyl phenyl ring), 7.74 (2H, d, *J* = 8.4 Hz, H-3′′, H-5′′). ^13^C NMR (100 MHz, DMSO-*d_6_*, *δ*): 34.55, 41.86, 100.58, 123.60(2 C), 126.17, 128.11(2 C), 129.29(4 C), 129.46 (2 C), 130.08, 130.25(2 C), 130.40, 131.19, 132.46, 132.73, 134.82, 142.79, 150.24, 170.06. ESI-MS (*m/z*): 430[M^+^], 431[M^+^+1], 432[M + 2]. CHNS Analysis for C_25_H_22_N_2_O_3_S Found (Calculated): C: 69.75(69.89), H: 5.15(5.17), N:6.51 (6.53), O:11.15 (11.11), S: 7.45 (7.55).

#### (E)-4-(2-(3-(4-fluorobenzylidene)-2-oxo-5-phenyl-2,3-dihydro-1H-pyrrol-1-yl)ethyl)benzenesulphonamide (3b)

Light yellow crystals, yield = 85%, m.p. 170–171 °C, R_f_ = 0.73[toluene: ethyl acetate: formic acid; 5:4:1]. IRυ_max_ (KBr, incm^−1^): 3743 and 3207 (NH_2_), 1676 (C = O), 1330 and 1150 (SO_2_N). ^1^H NMR (400 MHz, DMSO-*d_6_*, *δ*): 2.71(2H, *t*, PhCH_2_CH_2_N), 3.89 (2H, *t*, PhCH_2_CH_2_N), 6.39 (1H, *s*, H-4 pyrrolone ring), 7.12 (2H, d, *J* = 8.4 Hz, H-3, H-5), 7.25–7.64 (12H, *m*, SO_2_NH_2_, olefinic H and aromatic protons of Ar^1^, Ar^2^ and sulphamoyl phenyl ring), 7.74 (2H, d, *J* = 8.4 Hz, H-3′′, H-5′′).^13^C NMR (100 MHz, DMSO-*d_6_*, δ): 34.55, 41.86, 100.58, 123.60 (2 C), 126.17, 128.12 (2 C), 129.29 (4 C), 129.46 (2 C), 130.08, 130.25 (2 C), 130.40, 131.20, 132.46, 132.74, 134.83, 142.79, 150.25, 170.07. ESI-MS (*m/z*): 448[M^+^], 449[M^+^+1]. CHNS Analysis for C_25_H_21_FN_2_O_3_S Found (Calculated): C: 66.95(66.94), H: 4.72(4.73), F: 4.24(4.23); N:6.25 (6.27), O:10.70 (10.71), S: 7.15 (7.25).

#### (E)-4-(2-(3-(4-chlorobenzylidene)-2-oxo-5-phenyl-2,3-dihydro-1H-pyrrol-1-yl)ethyl)benzenesulphonamide (3c)

Light brown crystals, yield = 94%, m.p 192–193 °C, R_f_ = 0.72 [toluene: ethyl acetate: formic acid; 5:4:1]. IRυ_max_ (KBr, in cm^−1^): 3743 and 3207 (NH_2_), 1676 (C = O), 1330 and 1150 (SO_2_N). ^1^H NMR (300 MHz, CDCl_3_, *δ*): 2.82 (2H, *t*, PhCH_2_CH_2_N), 3.93 (2H, *t*, PhCH_2_CH_2_N), 4.81 (2H, *s*, SO_2_NH_2_), 6.08 (1H, *s*, H-4 pyrrolone ring), 7.07 (2H, d, *J* = 8.1 Hz, H-3′, H-5′), 7.36–7.44 (8H, *m*, aromatic protons of Ar^1^, Ar^2,^ sulphamoyl phenyl ring and olefinic H), 7.55 (2H, d, *J* = 8.1 Hz, H-2′, H-6′), 7.71 (2H, d, *J* = 8.1 Hz, H-3′′, H-5′′). ESI-MS (*m/z*): 464 [M^+^], 465[M^+^+1], 467[M^+^+3]. CHNS Analysis for C_25_H_21_ClN_2_O_3_S. Found (Calculated): C: 64.58(64.59), H:4.55 (4.51), Cl: 7.62(7.63), N:6.02(6.04), O:10.32 (10.31),S: 6.90 (6.93).

#### (E)-4-(2-(3-(4-bromobenzylidene)-2-oxo-5-phenyl-2,3-dihydro-1H-pyrrol-1-yl)ethyl)benzenesulphonamide (3d)

Dark brown crystals, yield = 89%, m.p. 214–215 °C, R_f_ = 0.71[toluene: ethyl acetate: formic acid; 5:4:1]. IRυ_max_ (KBr, incm^−1^): 3743 and 3207 (NH_2_), 1676 (C = O), 1330 and 1150 (SO_2_N). ^1^H NMR (400 MHz, DMSO-*d_6_*, δ): 2.71 (2H, *t*, PhCH_2_CH_2_N), 3.89 (2H, *t*, PhCH_2_CH_2_N), 6.39 (1H, *s*, H-4 pyrrolone ring), 7.12 (2H, d, *J* = 8.00 Hz, H-3′, H-5′), 7.25–7.64 (12H_,_ *m*, SO_2_NH_2_, olefinic H and aromatic protons of Ar^1^, Ar^2^ and sulphamoyl phenyl ring), 7.74 (2H, d, *J* = 8.00 Hz, H-3′′, H-5′′). ^13^C NMR (100 MHz, DMSO-*d_6_*, δ): 34.55, 41.86, 100.58, 123.60 (2 C), 126.17, 128.12 (2 C), 129.29(4 C), 129.46(2 C), 130.08, 130.25(2 C), 130.41, 131.20, 132.46, 132.74, 134.82, 142.79, 150.25, 170.20. ESI-MS (*m/z*): 509 [M^+^], 511[M^+^+2], 512[M^+^+3]. CHNS Analysis for C_25_H_21_BrN_2_O_3_S Found (Calculated): C: 58.94(58.91), H:4.16 (4.15), Br: 15.69(15.63), N:5.50(5.48), O: 9.42 (9.40), S: 6.29 (6.38).

#### (E)-4-(2-(3-benzylidene-2-oxo-5-(p-tolyl)-2,3-dihydro-1H-pyrrol-1-yl)ethyl)benzenesulphonamide (3e)

Light Yellow crystals, yield = 95%, m.p. 189-190 °C, R_f_ = 0.73[toluene: ethyl acetate: formic acid; 5:4:1]. IRυ_max_ (KBr, in cm^−1^): 3745 and 3191 (NH_2_), 1654 (C = O), 1331 and 1156 (SO_2_N). ^1^H NMR (400 MHz, DMSO-*d_6_*, *δ*): 2.50 (3H, s, CH_3_), 2.73 (2H, t, PhCH_2_CH_2_N), 3.89 (2H, t, PhCH_2_CH_2_N), 6.37(1H, s, H-4 pyrrolone ring), 7.15(2H, d, J = 8.4 Hz, H-3′ and H-5′ Ar^1^ ring), 7.27-7.66 (12H, m, SO_2_NH_2_, olefinic H, aromatic protons of Ar^1^, Ar^2^ and sulfamoyl phenyl ring), 7.77 (2H, d, J = 7.20 Hz, H-3′′, H-5′′). ^13^C NMR (100 MHz, DMSO-*d_6_*, δ): 21.44, 34.59, 41.84, 100.33, 126.17(3 C), 128.01(2 C), 128.44(2 C), 128.79(2 C), 129.47(2 C), 129.51(2 C), 129.79, 129.86, 130.15, 130.84, 131.49, 135.69, 139.72, 142.86, 170.24. ESI-MS (*m/z*): 444[M^+^], 445[M^+^+1], 446[M^+^+2], 447[M^+^+3]. CHNS Analysis for C_26_H_24_N_2_O_3_S Found (Calculated): C: 70.25(70.31), H: 5.44(5.45), N: 6.30(6.34), O: 10.80(10.71), S: 7.21 (7.28).

#### (E)-4-(2-(3-(4-fluorobenzylidene)-2-oxo-5-(p-tolyl)-2,3-dihydro-1H-pyrrol-1-yl)ethyl)benzenesulphonamide (3f)

Light Yellow crystals, yield = 92%, m.p. 196–197 °C, R_f_ = 0.72 [toluene: ethyl acetate: formic acid; 5:4:1]. IRυ_max_ (KBr, in cm^−1^): 3745 and 3191 (NH_2_), 1654 (C = O), 1331 and 1156 (SO_2_N). ^1^H NMR (400 MHz, DMSO-*d_6_*, δ): 2.38 (3H, *s*, CH_3_), 2.72 (2H, *t*, PhCH_2_CH_2_N), 3.88 (2H, *t*, PhCH_2_CH_2_N), 6.34 (1H, *s*, H-4 pyrrolone ring), 7.13–7.86 (15H, *m*, SO_2_NH_2_, olefinic H and aromatic protons of Ar^1^, Ar^2^ and sulphamoyl phenyl ring). ESI-MS (*m/z*): 462[M^+^]. CHNS Analysis for C_26_H_23_FN_2_O_3_S Found (Calculated): C: 67.51(67.41), H:5.01 (5.15), F:4.11(4.23), N: 6.06(6.14), O: 10.38 (10.37), S: 6.93 (6.92).

#### (E)-4-(2-(3-(4-chlorobenzylidene)-2-oxo-5-(p-tolyl)-2,3-dihydro-1H-pyrrol-1-yl)ethyl)benzenesulphonamide (3 g)

Dark brown crystals, yield = 96%; m.p. 224–225 °C, R_f_ = 0.71 [toluene: ethyl acetate: formic acid; 5:4:1]. IRυ_max_ (KBr, in cm^−1^): 3745 and 3191 (NH_2_), 1654 (C = O), 1331 and 1156 (SO_2_N). ^1^H NMR (400 MHz, DMSO-*d_6_*, δ): 2.38 (3H, *s*, CH_3_), 2.72 (2H, *t*, PhCH_2_CH_2_N), 3.88 (2H, *t*, PhCH_2_CH_2_N), 6.34 (1H, *s*, H-4 pyrrolone ring), 7.14 (2H, d, *J* = 8.4 Hz, H-3′, H-5′), 7.23–7.38 (7H, m, SO_2_NH_2_, olefinic H and aromatic protons of Ar^1^ and Ar^2^), 7.48 (2H, d, J = 8.8 Hz, H-2′′, H-6′′), 7.63 (2H, d, *J* = 8.00 Hz, H-2, H-6), 7.79 (2H, d, *J* = 8.00 Hz, H-3′′, H-5′′). ESI-MS (*m/z*): 478[M^+^], 479[M^+^+1], 481[M^+^+3]. CHNS Analysis for C_26_H_23_ClN_2_O_3_S Found (Calculated): C: 65.20(65.23), H:4.84(4.85), Cl:7.40(7.43), N:5.85(5.84), O:10.02(10.04), S: 6.69(6.68).

#### (E)-4-(2-(3-(4-bromobenzylidene)-2-oxo-5-(p-tolyl)-2,3-dihydro-1H-pyrrol-1-yl)ethyl)benzenesulphonamide (3h)

Dark brown crystals, yield = 98%, m.p. 233–234 °C, R_f_ = 0.70[toluene: ethyl acetate: formic acid; 5:4:1]. IR_υmax_ (KBr, in cm^−1^): 3745 and 3191(NH_2_), 1654 (C = O), 1331 and 1156 (SO_2_N). ^1^H NMR (400 MHz, DMSO-*d_6_*, δ): 2.38 (3H, *s*, CH_3_), 2.72 (2H, *t*, PhCH_2_CH_2_N), 3.88 (2H, *t*, PhCH_2_CH_2_N), 6.33 (1H, *s*, H-4 pyrrolone ring), 7.14 (2H, d, *J* = 8.4 Hz, H-3′ and H-5′ Ar^1^ ring), 7.21(*s*, 1H, olefinic H), 7.27–7.64 (10H, *m*, SO_2_NH_2_, aromatic protons of Ar^1^, Ar^2^ and sulphamoyl phenyl ring), 7.78 (2H, d, *J* = 8.00 Hz, H-3′′, H-5′′). ESI-MS(*m/z*):523[M^+^], 525[M^+^+2]. CHNS Analysis for C_26_H_23_BrN_2_O_3_S Found (Calculated): C: 59.66(59.61), H:4.43 (4.45), Br:15.27(15.29), N: 5.35(5.34), O: 9.17 (9.07), S: 6.13(6.18).

#### (E)-4-(2-(3-benzylidene-5-(4-ethylphenyl)-2-oxo-2,3-dihydro-1H-pyrrol-1-yl)ethyl)benzenesulphonamide (3i)

Light yellow crystals, yield = 90%, m.p. 183–184 °C, R_f_ = 0.75[toluene: ethyl acetate: formic acid; 5:4:1]. IRυ_max_ (KBr, in cm^−1^): 3390 and 3218 (NH_2_), 1653 (C = O), 1329 and 1155 (SO_2_N). ^1^H NMR (400 MHz, DMSO-*d_6_*, δ): 1.23 (3H, *t*, CH_3_CH_2_-), 2.65–2.74 (merged quartet of 2H of CH_3_CH_2_- with triplet of 2H of PhCH_2_CH_2_N), 3.89 (2H, *t*, PhCH_2_CH_2_N), 6.36 (1H, *s*, H-4 pyrrolone ring), 7.13 (2H, d, *J* = 7.60 Hz, H-3′ and H-5′), 7.24 (1H, *s*, olefinic H), 7.29 (2H, *s*, SO_2_NH_2_), 7.32–7.40 (5H, *m*, H-2′,H-6′, H-3, H-4, H-5), 7.48 (2H, d, *J* = 8.40 Hz, H-2′′ and H-6′′), 7.63(2H, d, *J* = 8.00 Hz, H-2 and H-6), 7.80(2H, d, *J* = 8.40 Hz, H-3′′ and H-5′′). ^13^C NMR (100 MHz, DMSO-*d_6_*, δ): 15.92, 28.51, 34.59, 41.85, 100.18, 126.16(2 C), 128.12(2 C), 128.59(2 C), 128.68(2 C), 129.45(2 C), 129.51, 129.88, 130.25, 132.47(2 C), 134.59, 134.62, 142.74, 142.82, 146.05, 150.31, 170.14. ESI-MS (*m/z*): 458[M^+^], 459[M^+^+1]. CHNS Analysis for C_27_H_26_N_2_O_3_S Found (Calculated): C: 70.72(70.71), H:5.71(5.68), N: 6.11(6.24), O:10.47 (10.57), S: 6.99(6.89).

#### (E)-4-(2-(5-(4-ethylphenyl)-3-(4-fluorobenzylidene)-2-oxo-2,3-dihydro-1H-pyrrol-1-yl)ethyl)benzenesulphonamide (3j)

Light yellow crystals, yield = 88%; m.p. 185–186 °C, R_f_ = 0.74[toluene: ethyl acetate: formic acid; 5:4:1]. IRυ_max_ (KBr, in cm^−1^): 3390 and 3218 (NH_2_), 1653 (C = O), 1329 and 1155 (SO_2_N). ^1^H NMR (400 MHz, DMSO-*d_6_*, δ): 1.19 (3H, *t*, CH_3_CH_2_-), 2.60–2.70 (quartet of 2H of CH_3_CH_2_ merged with triplet of 2H of PhCH_2_CH_2_N), 3.85 (2H, *t*, PhCH_2_CH_2_N), 6.33 (1H, *s*, H-4 pyrrolone ring), 7.09 (2H, d, *J* = 8.00 Hz, H-3′ and H-5′ Ar^1^ ring), 7.20 (1H, *s*, olefinic H), 7.26 (2H,s, SO_2_NH_2_), 7.30 (2H, d, *J* = 8.00 Hz, H-3 and H-5), 7.35 (2H, d, *J* = 8.00 Hz, H-2′ and H-6′), 7.45 (2H, d, *J* = 8.00 Hz, H-2′′ and H-6′′), 7.59 (2H, d, *J* = 8.00 Hz, H-2 and H-6), 7.77 (2H, d, *J* = 8.00 Hz, H-3′′ and H-5′′). ESI-MS (*m/z*):476[M^+^], 477[M^+^+1], 478[M^+^+2]. CHNS Analysis for C_27_H_25_FN_2_O_3_S Found (Calculated): C: 68.05(68.10), H:5.29 (5.39), F:3.99(3.89), N: 5.88(5.84), O: 10.07 (10.02), S: 6.73(6.78).

#### (E)-4-(2-(3-(4-chlorobenzylidene)-5-(4-ethylphenyl)-2-oxo-2,3-dihydro-1H-pyrrol-1-yl)ethyl)benzenesulphonamide (3k)

Dark brown crystals, yield = 95%, m.p. 186–187 °C, R_f_ = 0.73[toluene: ethyl acetate: formic acid; 5:4:1]. IRυ_max_ (KBr, in cm^−1^): 3390 and 3218 (NH_2_), 1653 (C = O), 1329 and 1155 (SO_2_N). ^1^H NMR (400 MHz, DMSO-*d_6_*, δ): 1.22 (3H, *t*, CH_3_CH_2_-), 2.65–2.74 (quartet of 2H of CH_3_CH_2_ merged with triplet of 2H of PhCH_2_CH_2_N), 3.89 (2H, *t*, PhCH_2_CH_2_N), 6.37 (1H, *s*, H-4 pyrrolone ring), 7.13 (2H, d, *J* = 8.40 Hz, H-3′ and H-5′ Ar^1^ ring), 7.24 (1H, *s*, olefinic H), 7.313(2H,s, SO_2_NH_2_), 7.33 (2H, d, *J* = 8.00 Hz, H-2′ and H-6′ Ar^1^ ring), 7.39 (2H, d, *J* = 8.40 Hz, H-3 and H-5), 7.48(2H, d, *J* = 8.00 Hz, H-2′′ and H-6′′) , 7.63 (2H, d, *J* = 8.00 Hz, H-2 and H-6), 7.81 (2H, d, *J* = 8.40 Hz, H-3′′ and H-5′′). ^13^C NMR (100 MHz, DMSO-*d_6_*, δ): 15.96, 28.51, 34.58, 41.83, 100.18, 126.16(2 C), 128.11(2 C), 128.58(2 C), 128.69(2 C), 129.46(2 C), 129.51, 129.89, 130.20, 132.49(2 C), 134.57, 134.62, 142.71, 142.82, 146.04, 150.28, 170.13. ESI-MS(*m/z*): 493[M^+^], 495[M^+^+2], 496[M^+^+3]. CHNS Analysis for C_27_H_25_ClN_2_O_3_S Found (Calculated): C:65.78(65.61), H:5.11(5.15), Cl:7.19(7.13), N:5.68(5.71), O:9.74(9.77), S:6.50(6.58).

#### (E)-4-(2-(3-(4-bromobenzylidene)-5-(4-ethylphenyl)-2-oxo-2,3-dihydro-1H-pyrrol-1-yl)ethyl)benzenesulphonamide (3 l)

Dark brown crystals, yield =90%; m.p. 188–189 °C, R_f_ = 0.72[toluene: ethyl acetate: formic acid; 5:4:1]. IRυ_max_ (KBr, in cm^−1^): 3390 and 3218 (NH_2_), 1653 (C = O), 1329 and 1155 (SO_2_N). ^1^H NMR (400 MHz, DMSO-*d_6_*, δ): 1.22 (3H, *t*, CH_3_CH_2_-), 2.67–2.73 (quartet of 2H of CH_3_CH_2_- merged with triplet of 2H of PhCH_2_CH_2_N), 3.88(2H, *t*, PhCH_2_CH_2_N), 6.35(1H, *s*, H-4 pyrrolone ring), 7.12(2H, d, *J* = 8.40 Hz, H-3′ and H-5′), 7.24(1H, *s*, olefinic H), 7.26 (2H, *s*, SO_2_NH_2_), 7.33(2H, d, *J* = 8.80 Hz, H-2′ and H-6′), 7.38(2H, d, *J* = 8.40 Hz, H-2′′ and H-6′′), 7.48(2H, d, *J* = 8.00 Hz, H3, H5), 7.62(2H, d, *J* = 8.00 Hz, H-2 and H-6), 7.80(2H, d, *J* = 8.80 Hz, H-3′′ and H-5′′). ESI-MS (*m/z*):537[M^+^], 539[M^+^+2], 540[M^+^+3]. CHNS Analysis for C_27_H_25_BrN_2_O_3_S Found (Calculated):C: 60.34(60.31), H:4.69 (4.65), Br:14.87(14.83), N: 5.21(5.34), O: 8.93(8.94), S: 5.97(5.88).

#### (E)-4-(2-(3-benzylidene-5-(4-chlorophenyl)-2-oxo-2,3-dihydro-1H-pyrrol-1-yl)ethyl)benzenesulphonamide (3 m)

Light yellow crystals, yield = 87%, m.p. 189–190 °C, R_f_ = 0.73[toluene: ethyl acetate: formic acid; 5:4:1]. IRυ_max_ (KBr, in cm^−1^): 3456 and 3190 (NH_2_), 1670 (C = O), 1323 and 1148 cm^−1^ (SO_2_N).^1^H NMR (400 MHz, DMSO-*d_6_*, δ): 2.74 (2H, *t*, PhCH_2_CH_2_N), 3.89 (2H, *t*, PhCH_2_CH_2_N), 6.43 (1H, *s*, H-4 pyrrolone ring), 7.14 (2H, d, *J* = 8.00 Hz, H-2′ and H-6′), 7.31–7.46 (8H, *m*, SO_2_NH_2_, olefinic H and aromatic protons of Ar^1^, Ar^2^), 7.54 (2H, d, *J* = 8.40 Hz, H-2′′ and H-6′′),7.63(2H, d, *J* = 8.40 Hz, H-2 and H-6), 7.805 (2H, d, *J* = 8.00 Hz, H-3′′ and H-5′′). ^13^C NMR (125 MHz, DMSO-*d_6_*, δ): 34.54, 41.83, 100.1, 126.17(2 C), 129.34, 129.53(4 C), 129.91(2 C), 130.05(2 C), 130.40, 130.97(2 C), 132.40(2 C), 134.63, 135.52, 142.76, 142.81, 148.48, 170.10. ESI-MS(*m/z*): 464[M^+^], 465[M^+^+1]. CHNS Analysis for C_25_H_21_ClN_2_O_3_S Found (Calculated): C:64.58(64.51), H:4.55(4.53), Cl:7.62(7.63), N: 6.02(6.04), O: 10.32 (10.37), S: 6.90(6.98).

#### (E)-4-(2-(5-(4-chlorophenyl)-3-(4-fluorobenzylidene)-2-oxo-2,3-dihydro-1H-pyrrol-1-yl)ethyl)benzenesulphonamide (3n)

Light yellow crystals, yield = 88%, m.p. 159-160 °C, R_f_ = 0.72[toluene: ethyl acetate: formic acid; 5:4:1]. IRυ_max_ (KBr, in cm^−1^): 3456 and 3176 (NH_2_), 1670 (C = O), 1330 and 1156 cm^−1^ (SO_2_N). ^1^H NMR (400 MHz, DMSO-*d_6_*, δ): 2.74 (2H, *t*, PhCH_2_CH_2_N), 3.89 (2H, *t*, PhCH_2_CH_2_N), 6.43 (1H, *s*, H-4 pyrrolone ring), 7.17 (2H, d, *J* = 8.00 Hz, H-3 and H-5), 7.32 (2H, d, *J* = 8.00 Hz, H-2′ and H-6′), 7.43–7.47 (5H, *m*, SO_2_NH_2_, olefinic H and H-3′, H-5′), 7.54 (2H, d, *J* = 8.00 Hz, H-2′′ and H-6′′), 7.64(2H, d, *J* = 8.00 Hz, H-2 and H-6), 7.79 (2H, d, *J* = 8.00 Hz, H-3′′ and H-5′′). ^13^C NMR (125 MHz, DMSO-*d_6_*, δ): 34.59, 41.95, 100.94, 126.16(2 C), 129.36, 129.62(4 C), 129.95(2 C), 130.09(2 C), 130.44, 130.96(2 C), 132.33(2 C), 134.68, 135.54, 142.79, 142.87, 148.53, 170.10. ESI-MS(*m/z*): 482[M^+^], 483[M^+^+1], 485[M^+^+3]. CHNS Analysis for C_25_H_20_ClFN_2_O_3_S Found (Calculated): C: 62.17(62.21), H:4.17(4.25), Cl:7.34(7.43), F:3.93(3.87), N: 5.80(5.84), O: 9.94 (9.97),S: 6.64(6.68).

#### (E)-4-(2-(3-(4-chlorobenzylidene)-5-(4-chlorophenyl)-2-oxo-2,3-dihydro-1H-pyrrol-1-yl)ethyl)benzenesulphonamide (3o)

Light brown crystals, yield = 77%; m.p. 163–164 °C, R_f_ = 0.71[toluene: ethyl acetate: formic acid; 5:4:1]. IRυ_max_ (KBr, in cm^−1^): 3456 and 3176 (NH_2_), 1670 (C = O), 1330 and 1156 cm^−1^ (SO_2_N). ^1^H NMR (400 MHz, DMSO-*d_6_*, δ): 2.73(2H, *t*, PhCH_2_CH_2_N), 3.87(2H, *t*, PhCH_2_CH_2_N), 6.41(1H, *s*, H-4 pyrrolone ring), 7.12(2H, d, *J* = 8.40 Hz, H-2′ and H-6′), 7.21–7.53 (9H, *m*, SO_2_NH_2_, olefinic H, H-3, H-5, H-2′′, H-6′′and H-3′, H-5′), 7.62(2H, d, *J* = 8.00 Hz, H-2 and H-6), 7.81(2H, d, *J* = 8.00 Hz, H-3′′ and H-5′′). ESI-MS (*m/z*): 499[M^+^], 500[M^+^+1], 501[M^+^+2]. CHNS Analysis for C_25_H_20_Cl_2_N_2_O_3_S Found (Calculated): C:60.12(60.13), H:4.04(4.05), Cl:14.20(14.23), N: 5.61(5.64), O: 9.61 (9.67), S: 6.42(6.43).

#### (E)-4-(2-(3-(4-bromobenzylidene)-5-(4-chlorophenyl)-2-oxo-2,3-dihydro-1H-pyrrol-1-yl)ethyl)benzenesulphonamide (3p)

Dark brown crystals, yield = 85%, m.p. 176–177 °C, R_f_ = 0.70 [toluene: ethyl acetate: formic acid; 5:4:1]. IRυ_max_ (KBr, in cm^−1^): 3456 and 3176 (NH_2_), 1670 (C = O), 1330 and 1156 cm^−1^ (SO_2_N). ^1^H NMR (400 MHz, DMSO-*d_6_*, δ): 2.73 (2H, *t*, PhCH_2_CH_2_N), 3.87 (2H, *t*, PhCH_2_CH_2_N), 6.40(1H, *s*, H-4 pyrrolone ring), 7.12 (2H, d, *J* = 8.00 Hz, H-2′ and H-6′), 7.35 (1H, *s*, olefinic H), 7.37 (2H, *s*, SO_2_NH_2_), 7.44 (2H, d, *J* = 8.00 Hz, H-3′ and H-5′), 7.52 (2H, d, *J* = 8.00 Hz, H-2′′ and H-6′′), 7.61–7.65 (4H_,_ *m*, H-2, H-3, H-5, H-6), 7.72 (2H, d, *J* = 8.00 Hz, H-3′′ and H-5′′). ESI-MS (*m/z*): 542[M^+^-1], 543[M^+^]. CHNS Analysis for C_25_H_20_BrClN_2_O_3_S Found(Calculated): C:55.21(55.31), H:3.71(3.75), Br:14.69(14.63), Cl:6.52(6.57), N: 5.15(5.14), O: 8.83(8.87), S: 5.90(5.88).

#### (E)-4-(2-(3-benzylidene-5-(4-(tert-butyl)phenyl)-2-oxo-2,3-dihydro-1H-pyrrol-1-yl)ethyl)benzenesulphonamide (3q)

Light brown crystals, yield = 87%, m.p. 183–184 °C, R_f_ = 0.71[toluene: ethyl acetate: formic acid; 5:4:1].IRυ_max_ (KBr, in cm^−1^): 3747 and 3208 (NH_2_), 1675 (C = O), 1330 and 1151 cm^−1^ (SO_2_N). ^1^HNMR (400 MHz, DMSO-*d_6_*, δ): 1.28 (9H, *s*, 3 × CH_3_), 2.67(2H, *t*, PhCH_2_CH_2_N), 3.84 (2H, *t*, PhCH_2_CH_2_N), 6.33(1H, *s*, H-4 pyrrolone ring), 7.06 (2H, d, *J* = 8.00 Hz, H-3′ and H-5′), 7.21 (1H,s olefinic H), 7.24(2H, *s*, SO_2_NH_2_), 7.35 (2H, d, *J* = 8.00 Hz, H-2′ and H-6′), 7.44–7.48 (5H, *m*, H-3, H-4, H-5, H-2′′, H-6′′), 7.57 (2H, d, *J* = 8.00 Hz, H-2 and H-6), 7.77(2H, d, *J* = 8.00 Hz, H-3′′ and H-5′′). ESI-MS(*m/z*): 486[M^+^], 487[M^+^+1]. CHNS Analysis for C_29_H_30_N_2_O_3_S Found (Calculated): C: 71.58(71.56), H:6.21(6.25), N: 5.76(5.84), O: 9.86 (9.97), S: 6.59(6.58).

#### (E)-4-(2-(5-(4-(tert-butyl)phenyl)-3-(4-fluorobenzylidene)-2-oxo-2,3-dihydro-1H-pyrrol-1-yl)ethyl)benzenesulphonamide (3r)

Light yellow crystals, yield = 77%, m.p. 187–188 °C, R_f_ = 0.71[toluene: ethyl acetate: formic acid; 5:4:1]. IRυ_max_ (KBr, in cm^−1^): 3456 and 3286 (NH_2_), 1673 (C = O), 1375 and 1178 cm^−1^ (SO_2_N). ^1^H NMR (400 MHz, DMSO-*d_6_*, δ): 1.27 (9H, *s*, 3 × CH_3_), 2.67 (2H, *t*, PhCH_2_CH_2_N), 3.86 (2H, *t*, PhCH_2_CH_2_N), 6.34 (1H, *s*, H-4 pyrrolone ring), 7.06 (2H, d, *J* = 8.00 Hz, H-3 and H-5), 7.21 (1H, *s*, olefinic H), 7.26 (2H, *s*, SO_2_NH_2_), 7.35(2H, d, *J* = 8.00 Hz, H-3′ and H-5′), 7.44–7.48 (4H, *m*, H-2′, H-6′, H-2′′, H-6′′), 7.57 (2H, d, *J* = 8.00 Hz, H-2 and H-6), 7.77 (2H, d, *J* = 8.00 Hz, H-3′′ and H-5′′). ESI-MS(*m/z*): 504[M^+^], 505[M^+^+1], 506[M^+^+2]. CHNS Analysis for C_29_H_29_FN_2_O_3_S Found (Calculated): C: 69.02(69.07), H:5.79 (5.75), F:3.76(3.77), N: 5.55(5.54), O: 9.51 (9.57), S: 6.35(6.38).

#### (E)-4-(2-(5-(4-(tert-butyl)phenyl)-3-(4-chlorobenzylidene)-2-oxo-2,3-dihydro-1H-pyrrol-1-yl)ethyl)benzenesulphonamide (3s)

yellow crystals, yield = 95%, m.p. 196–197 °C, R_f_ = 0.70[toluene: ethyl acetate: formic acid; 5:4:1].IRυ_max_ (KBr, in cm^−1^): 3456 and 3286 (NH_2_), 1673 (C = O), 1375 and 1178 cm^−1^ (SO_2_N). ^1^H NMR (400 MHz, DMSO-*d_6_*, δ): 1.30 (9H, *s*, 3 × CH_3_), 2.67 (2H, *t*, PhCH_2_CH_2_N), 3.86 (2H, *t*, PhCH_2_CH_2_N), 6.32(1H, *s*, H-4 pyrrolone ring), 7.06 (2H, d, *J* = 8.00 Hz, H-3′ and H-5′), 7.21 (1H, *s*, olefinic H), 7.25 (2H, *s*, SO_2_NH_2_), 7.35(2H, d, *J* = 8.00 Hz, H-2′ and H-6′), 7.40–7.48 (4H_,_ *m*, H-3, H-5, H-2′′, H-6′′), 7.57(2H, d, *J* = 8.00 Hz, H-2 and H-6), 7.77 (2H, d, *J* = 8.00 Hz, H-3′′ and H-5′′). ESI-MS(*m/z*): 521[M^+^], 522[M^+^+1]. CHNS Analysis for C_29_H_29_ClN_2_O_3_S Found (Calculated): C: 66.85(66.81), H:5.61 (5.65), Cl:6.80(6.87), N: 5.38(5.34), O: 9.21 (9.37), S: 6.15(6.18).

#### (E)-4-(2-(3-(4-bromobenzylidene)-5-(4-(tert-butyl)phenyl)-2-oxo-2,3-dihydro-1H-pyrrol-1-yl)ethyl)benzenesulphonamide (3t)

Dark brown crystals, yield = 92%, m.p. 204–205 °C, R_f_ = 0.70[toluene: ethyl acetate: formic acid; 5:4:1]. IRυ_max_ (KBr, in cm^−1^): 3456 and 3286(NH_2_), 1673 (C = O), 1375 and 1178 cm^−1^ (SO_2_N). ^1^H NMR (400 MHz, DMSO-*d_6_*, δ): 1.28 (9H, *s*, 3 × CH_3_), 2.66 (2H, *t*, PhCH_2_CH_2_N), 3.85 (2H, *t*, PhCH_2_CH_2_N), 6.34 (1H, *s*, H-4 pyrrolone ring), 7.06 (2H, d, *J* = 8.00 Hz, H-3′ and H-5′), 7.21 (1H, *s*, olefinic H), 7.25 (2H, *s*, SO_2_NH_2_), 7.35 (2H, d, *J* = 8.00 Hz, H-2′ and H-6′), 7.43–7.48 (4H, *m*, H-3, H-5, H-2′′, H-6′′), 7.57 (2H, d, *J* = 8.00 Hz, H-2 and H-6), 7.77 (2H, d, *J* = 8.00 Hz, H-3′′ and H-5′′). ESI-MS(*m/z*): 565[M^+^], 567[M^+^+2]. CHNS Analysis for C_29_H_29_BrN_2_O_3_S Found (Calculated): C: 61.59(61.56), H:5.17 (5.25), Br:14.13(14.17), N: 4.95(4.84), O: 8.49 (8.57),S: 5.67(5.68).

## Carbonic anhydrase inhibition assay

By using Stopped-Flow CO_2_ hydrase assay, derivatives **3a–t** were tested as inhibitors of the hCAs I, II, IX, and XII. Acetazolamide (AAZ), a commonly used sulphonamide inhibitor was used for comparing the inhibition data. The Inhibition effects of these compounds on enzyme activities were evaluated under in-vitro conditions. The CA catalysed CO_2_ hydration activity was measured using an Applied Photophysics stopped-flow instrument. Working at the maximum absorbance of 557 nm, phenol red (at a concentration of 0.2 mM) has been employed as an indicator, with 20 mM Na_2_SO_4_ (for maintaining constant the ionic strength) and 20 mM Hepes (pH 7.5) as buffer, following the initial rates of the CA-catalysed CO_2_ hydration reaction for a duration of 10–100 s. The CO_2_ concentrations used to determine the inhibition constants and kinetic parameters ranged from 1.7 to 17 mM. At least six traces of the initial 5–10% of the reaction have been used for measuring the initial velocity for every inhibitor. In the same way, the uncatalyzed rates were analysed and deducted from the total measured rates. Preparation of stock solutions of the inhibitor (0.1 mM) in distilled- deionised water was carried out, followed by the dilutions up to 0.05 nM by the assay buffer. The inhibitor and enzyme solutions were preincubated at room temperature for 15 min prior to the experiment to promote the formation of the E-I complex. By employing nonlinear least-squares methods using PRISM 3 and the Cheng-Prusoff equation, the inhibition constants were determined, as was previously reported^18–20^^,^^21^ and indicate the average of at least three separate measurements that fall between the range of 5 and 10%. As reported earlier^22–24^ all isoforms were recombinant ones obtained in house and their concentrations in the assay system was in the range of 6.8 − 12.4 nM.

## ADMET Analysis

Using the Qikprop module of Maestro 9.6 Schrodinger programme, the ADMET evaluations of the compounds **(3a-t)** were carried out. It offers a set of parameters to compare a given molecule’s characteristics with 95% of all the known drugs. Various molecular descriptors were calculated that influence the physicochemical characteristics of a molecule. Based on the chemical structures of the molecules, the theoretical parameters were computed. Various descriptors such as human oral absorption, gut–blood barrier permeability, partition coefficient, CNS (central nervous system) activity were calculated^25^.

## DFT study for E and Z isomers of compound 3n

Density functional theory (DFT) studies were carried for both the geometrical isomers (*E* and *Z*) of compound **3n** using ORCA Program Package Version 4.2^26^. We employed B3LYP functional^27^^,^^28^ and def2-TZVPD3BJ basis sets^29^ to perform the DFT calculations of the compounds.

## Results and discussion

### Chemistry

The current research is an expansion of our ongoing initiative^12^^,^^30–33^ to create potentially and biologically active drugs by adopting a hybrid pharmacophore strategy. Designing and synthesising of hybrid molecules were achieved by joining the pyrrolone ring through two methylene groups with benzene sulphonamide. Here, we present the synthesis and inhibitory profile of twenty novel (*E*)-1–(4-sulfamoylphenylethyl)-3-arylidene-5-aryl-1H-pyrrol-2(3H)-ones **(3a-t)** against two cystosolic (hCA I and II) and two membrane bound, tumour-related hCA IX and XII isoforms. The exocyclic double bond in the target compounds **(3a–t)** allows for the possibility of either *E* or *Z*-isomers. The (*E*)-configuration appears to be suggested by calculations of δ values using incremental parameters for the hydrogen (semicyclic double bond). This is consistent with the results that have previously been reported^14^. A nuclear overhauser effect (NOE) experiment was carried to analyse the compound **3k** in order to ascertain the geometry of the target compounds. The interaction between an olefinic proton and H-4 on the pyrrolone ring would be clearly visible through NOE analysis if **3k** is the *Z* isomer. The fact that an olefinic proton did not engage in interaction with H-4 on the pyrrolone ring suggests the *E* form (Figure S1 and S2). The outcome of the inhibitory efficacy of such sulphonamide derivatives displayed good results and further strengthened the significance of the sulphonamides in the medicinal chemistry field of CAIs.

**Scheme 1.**
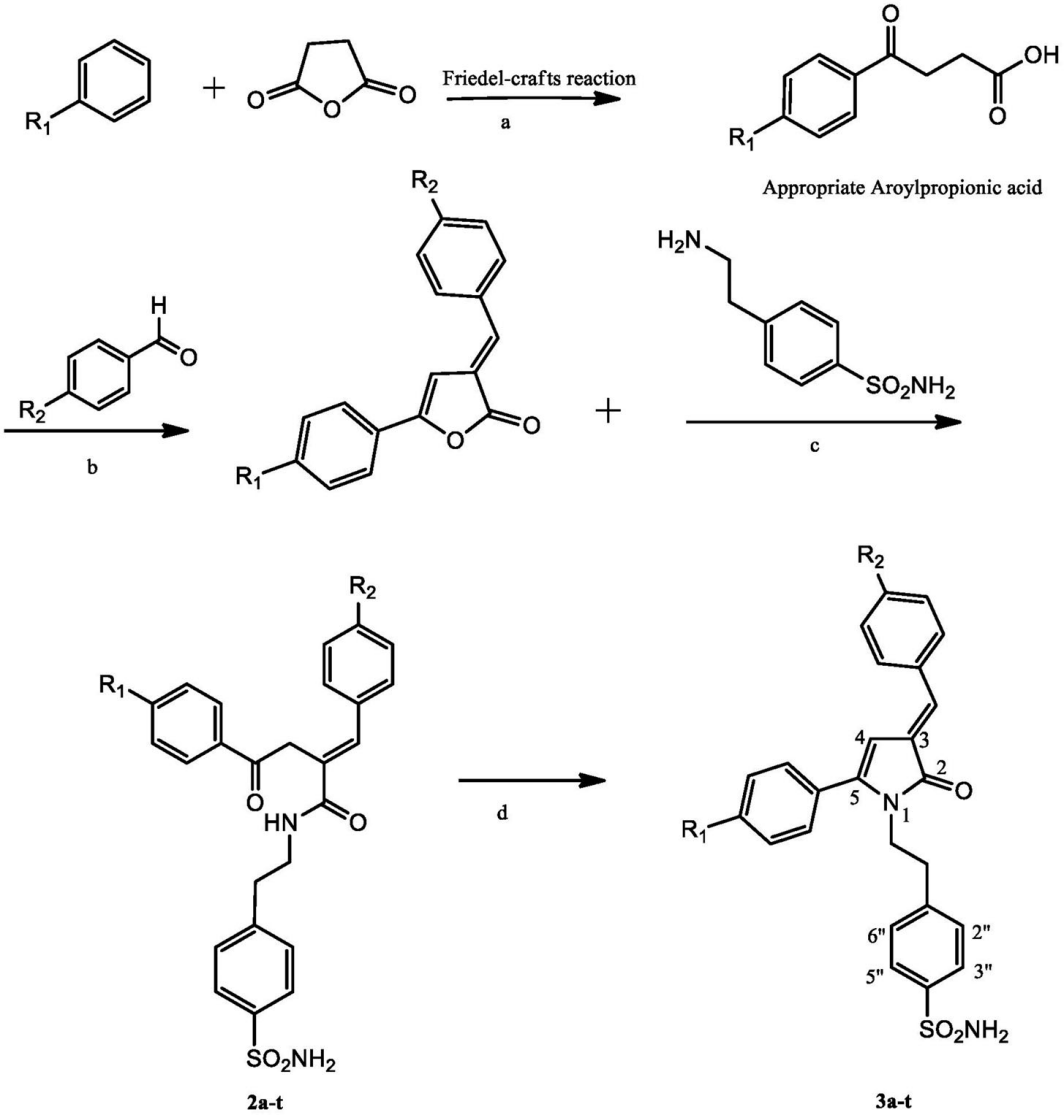
Synthesis of pyrrolone substituted ethyl benzene sulphonamide derivatives **(3a-t)**. Reagents and conditions (a) Anhydrous AlCl_3_, stirring at room temperature, 6h; (b) Acetic anhydride, Triethylamine, reflux, 4-5 h; (c) Dry benzene, Reflux, 4h; (d) 6M HCl, reflux, 2h). **1a-3a, 1b-3b, 1c-3c, 1d-3d;** R_1_=H,**1e-3e, 1f-3f, 1g-3g, 1h-3h;** R_1_= CH_3,_ **1i-3i, 1j-3j, 1k-3k, 1l-3l;** R_1_**=**C_2_H_5_, **1m-3m, 1n-3n, 1o-3o, 1p-3p;** R_1_=Cl, **1q-3q, 1r-3r, 1s-3s, 1t-3t;** R_1_=-C(CH_3_)_3_, **1a-3a, 1e-3e, 1i-3i, 1m-3m, 1q-3q;** R_2_=H, **1b-3b, 1f-3f, 1j-3j, 1n-3n, 1r-3r;** R_2_=F, **1c-3c, 1g-3g, 1k-3k, 1o-3o, 1s-3s;** R_2_=Cl, **1d-3d, 1h-3h, 1l- 3l, 1p-3p, 1t-3t;** R_2_=Br_._

Synthesis of target compounds (**3a-t)** was accomplished using a four-step procedure described in Scheme 1. Cyclisation of appropriate *γ*-keto-4-sulfamoylphenylethylamide (**2a-t)** in HCl yields (*E*)-1–(4-sulphamoylphenylethyl)-3-arylidene-5-aryl-1*H*-pyrrol-2(3H)-ones (**3a-t)**. Treatment of appropriate *E* form of 3-arylidene-5-(aryl)-furan-2(3H)-ones (**1a-t)** with equimolar quantity of 4–(2-aminoethyl)benzenesulphonamide in dry benzene yields compounds (**2a**-**t)**.The desired intermediates *i.e., E* form of 3-arylidene-5-(aryl)-furan-2(3H)-ones (**1a-t)** were synthesised by fusion of appropriate 1,4-ketoacids (*β*-aroylpropionic acids) and aromatic aldehyde using acetic anhydride in presence of triethylamine through reported method^34^^,^^35^. Compound purity was monitored using TLC plates (silica gel G), and was further detected by exposure to UV lamp and iodine vapours.

The structures of pyrrolone derivatives (**3a-t)** were confirmed by spectroscopic methods such as IR, ^1^H NMR, ^13^C NMR, MS and elemental analysis. Compounds were obtained in the good yield of around 70–95%. Characteristic stretching bands in the IR spectra for compounds **3a-t** were observed: two bands for NH_2_ (3747–3176 cm^−1^ and 3227–3286 cm^−1^), one band for carbonyl group of pyrrolone moiety (1676–1653 cm^−1^) and two bands for SO_2_N (1375–1323 cm^−1^ and 1178–1148 cm^−1^). ^1^H NMR spectrum showed two protons triplets at δ = 2.60–2.82 and δ = 3.84–3.93 which can be ascribed to -CH_2_-CH_2_- unit. One proton singlet which appeared at δ = 6.08–6.43 can be assigned to H-4 of pyrrolone ring. SO_2_NH_2_ protons appeared as singlet at δ = 4.81–7.43. Another proton singlet appeared at δ = 7.20–7.36 and is accountable for olefinic-H. In addition, Density Functional Theory (DFT) calculations of **3n-*E* and 3n-*Z*** were performed to gain more understanding about the stability of the *E* and *Z* isomers. The energy values clearly indicate the stability of *E*-isomer over the *Z*-isomer by −8.2 kJ mol^−1^ which was further supported by Frontier Molecular Orbital (FMO) analysis of these isomers. In the ^13^C NMR spectra, chemical shift value of carbon atoms, were noticed in the expected range with -CH_2_-CH_2_-N- unit carbon peaks appearing at (δ = 34.54–34.59) and (δ = 41.83–41.95) respectively while as C-4 of pyrrolone ring appeared in range of (δ =100.1–100.94) and the peak of C-2 appeared at (δ =170.06 − 170.24).

## Carbonic anhydrase inhibition activity

In the current study, Carbonic anhydrase inhibitory effect of 20 novel synthesised compounds (**3a-t)** was tested under *in vitro* condition against two cystolic (hCA I and II) and membrane bound tumour related isoforms i.e., hCA IX and XII, along with the standard inhibitor acetazolamide (AAZ) in a stopped-flow CO_2_ hydrase assay^36^. The inhibition data (K_i_) was determined with the help of Cheng-Prusoff equation as mentioned in the Materials and methods section. In selecting these four isoforms it was taken into account that hCA II is a target site for glaucoma^37^ and hCA IX and XII have been authenticated as hypoxic malignancy targets^38^^,^^39^. However, for anti-glaucoma and anticancer CAIs therapeutic applications^6^^,^^40^ hCA I is considered as the essential off-target isoform.

Based on Table 1 of the CA inhibition assay, following assumptions were made:

**Table 1.** Carbonic anhydrase inhibition data of compounds (**3a-t)**.

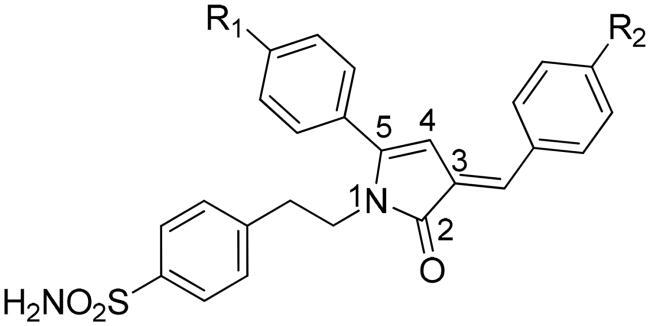						
Compoud Code	R_1_	R_2_	Ki(nM)			
			hCA I	hCA II	hCA IX	hCA XII
**3a**	-H	-H	634.1	368.5	93.8	62.1
**3b**	-H	-F	368.7	81.4	72.7	45.6
**3c**	-H	-Cl	3250	718.0	108.3	76.5
**3d**	-H	-Br	4245	1426	111.6	89.8
**3e**	-CH_3_	-H	3204	471.4	116.7	58.4
**3f**	-CH_3_	-F	2526	146.9	103.2	42.7
**3g**	-CH_3_	-Cl	4385	941.7	95.5	73.4
**3h**	-CH_3_	-Br	4187	1871	84.2	94.0
**3i**	-CH_2_CH_3_	-H	1788	901.5	78.3	83.9
**3j**	-CH_2_CH_3_	-F	836.5	295.7	71.4	66.2
**3k**	-CH_2_CH_3_	-Cl	5932	1624	101.6	91.1
**3l**	-CH_2_CH_3_	-Br	6764	3710	77.5	56.3
**3m**	-Cl	-H	2263	780.0	54.6	61.7
**3n**	-Cl	-F	1416	465.6	41.3	39.1
**3o**	-Cl	-Cl	5088	1892	68.9	78.9
**3p**	-Cl	-Br	5866	3848	74.3	85.4
**3q**	-C(CH_3_)_3_	-H	8495	1647	468.5	396.2
**3r**	-C(CH_3_)_3_	-F	5997	1263	417.7	355.7
**3s**	-C(CH_3_)_3_	-Cl	7932	4355	432.4	341.5
**3t**	-C(CH_3_)_3_	-Br	9272	4687	391.2	308.0
**AAZ**	–	–	250.0	12.0	25.0	5.7

Mean from 3 different assays by a stopped flow technique (errors between 5–10% of the reported values)

- The inhibition profile of the compounds against the four CA isoforms was affected by the substitution of halogen groups at the *para* position of the arylidene ring (C-3) directly attached to pyrrolone nucleus. Compound **3b** was found to be most active inhibitor against membrane bound cytosolic hCAI and II isoform having K_I_ values of 368.7 and 81.4 nM respectively while as **3n** was found to be more effective against the tumour**-**associated hCA IX and XII isoforms having K_i_ value of 41.3 and 39.1 nM respectively. Hydrophobicity of the pyrrolone moiety may facilitate the inhibition of hCA I, II, IX and XII isozymes. It was further noted that by introducing a strong electron withdrawing group, that is, fluoro at the *para* position of arylidene ring (C-3) the inhibitory activity of such compounds increased because of strong binding affinity to the active site of enzyme thus making such compounds efficient and tight binding inhibitors than the rest of the compounds of the series.^41–43^ Alternatively other functionalisations also adversely effect the inhibition potency, *i.e.* incorporation of less hindered groups at the *para* position of aryl ring (C-5) of pyrrolone nucleus, maintained certain degree of activity than more hindered groups. Overall, the SAR analysis reveals that hydrophobic and bulky substituents are detrimental to all isoforms; however, the presence of a fluorine atom always improves Ki values except for the simultaneous presence of tertiary butyl substituent.
- All pyrrolone substituted ethyl benzene sulphonamides showed K_is_ between 368.7- 9272nM for the cytosolic isoform hCAI. Compound **3b** had effective inhibition for this isoform **(**K_i_ = 368.7**),** while as **3a** and **3j** had moderate inhibition activity with (K_iS_ = 634.1 and 836.5 nM) and **3q** and **3t** lead to weaker inhibition in hCAI isoform (K_iS=_ 8.5 and 9.3µM) respectively.
- hCAII isoform was inhibited more efficiently by sulphonamide derivatives (K_iS_=81.4- 4687nM) compared to hCAI. Compound **3b** was more effective inhibitor for this isoform (K_i_=81.4nM). However, compounds **3a, 3c, 3e, 3f, 3g, 3i, 3j, 3m, 3n** had medium nanomolar inhibition activity (K_iS_=368.5, 718, 471.4, 146.9, 941.7, 901.5, 295.7, 780, 465.6nM) respectively, whereas compounds **3s** and **3t** (K_iS_=4.3 and 4.6µM) had weak inhibition activity.
- All these primary sulphonamides exhibited significant potency towards the tumour associated isoform hCA IX among all the isoforms (K_is_ spanning between 41.3 – 468.5nM). Compound **3n** was the most potent inhibitor for this isoform (K_i_ = 41.3 nM) relative to AAZ (K_i_ = 25.7nM). However, compounds **3a, 3b, 3g, 3h, 3i, 3j, 3l, 3m, 3o, 3p** having (K_is_=93.8, 72.7, 95.5, 84.2, 78.3, 71.4, 77.5, 54.6, 68.9, 74.3 nM) moderately inhibited the hCA IX isoform, whereas other compounds **3c, 3d, 3e, 3f, 3k, 3q, 3r, 3s, 3t** (K_is_ = 108.3, 111.6, 116.7, 103.2, 101.6, 468.5, 417.7, 432.4, 391.2 nM) showed lower binding affinity, in comparison to other compounds of the series.
- The other tumour-associated isoform, that is, hCA XII showed similar tendencies as described above for hCA IX, with K_is_ ranging from 39.1 to 396.2 nM. Compound **3n** was the most potent inhibitor for this isoform (K_i_ = 39.1nM).Compounds **3a-m** (K_is_=62.1, 45.6, 76.5, 89.8, 58.4, 42.7, 73.4, 94.0, 83.9, 66.2, 91.1, 56.3, 61.7 nM), **3o-p** (K_is_ = 78.9 and 85.4 nM) moderately inhibited hCA XII, whereas **3q-t** (K_iS_=0.39, 0.35, 0.34, 0.30µM**)** showed lower binding affinity in comparison to other compounds of the series.
- From the brief examination of the data, it is evident that the compounds that have been synthesised and investigated for the activity showed low to high nanomolar efficacy against cytosolic hCA II isoform while the efficiency towards hCA I decreased dramatically. Also the inhibitory efficacy towards tumour associated isoform *i.e.* hCA IX and hCA XII settled in the low nanomolar range, therefore, making this distinct series of compounds worthwhile, thus indicating new class of possible CAIs of medicinal interest.

## ADMET Analysis

The ADMET analysis of **3a-t** were carried with the Qikprop tool of Schrodinger. Molecular descriptors were calculated that impact the physicochemical characteristics of a compound. In the present ADMET analysis, drug-likeness properties and in silico ADME evaluation for all synthesised compounds were analysed. The different parameters predicted were; molecular weight (M. Wt.), number of hydrogen bond donor (HBD), number of hydrogen bond acceptor (HBA), octanol/water partition coefficient (log P) and Percentage of human oral absorption. Lipinski’s rule of five values are within their acceptable range, except for the molecular weight. Most of the titled compounds were following the parameters that were followed by majority of the approved drugs. Partition coefficient predicted values were within the acceptable range. Despite of all parameter, human oral absorption were found to be higher for all compounds and within the limit while as **3k** and **3q** had highest human oral absorption. The results are presented in Table 2.

**Table 2.** ADMET analysis of the compounds against hCAI, hCAII, hCAIX and hCAXII isozymes.

Compound code	ADMET Analysis				
	QPlogP o/w^a^	Rule of five^b^	Donor HB^c^	Acceptor HB^d^	% HOA^e^
**3a**	3.6	0	2	7.5	90.20
**3b**	3.85	0	2	7.5	91.63
**3c**	4.172	0	2	7.5	91.70
**3d**	4.157	1	2	7.5	80.45
**3e**	3.945	0	2	7.5	90.74
**3f**	4.206	0	2	7.5	92.26
**3g**	4.458	0	2	7.5	93.73
**3h**	4.547	1	2	7.5	81.41
**3i**	4.296	0	2	7.5	95.02
**3j**	4.456	0	2	7.5	95.57
**3k**	4.777	0	2	7.5	100
**3l**	4.789	1	2	7.5	84.56
**3m**	4.121	0	2	7.5	93.41
**3n**	4.311	0	2	7.5	94.28
**3o**	4.568	0	2	7.5	95.79
**3p**	4.647	1	2	7.5	83.31
**3q**	4.534	0	2	7.5	100
**3r**	4.741	1	2	7.5	88.09
**3s**	4.996	1	2	7.5	89.58
**3t**	5.07	2	2	7.5	77.06

Abbreviations. ADMET: absorption, distribution, metabolism, excretion and toxicity; HOA: human oral absorption; ^a^Predicted octanol/water partition coefficient; ^b^Lipinski’s violations (≤1); ^c^Donor HB (0–6); ^d^Acceptor HB (2–20); ^e^%HOA >80% is high, 25% is low.

## DFT study for *E* and *Z* isomers of compound 3n

**Figure 3.**
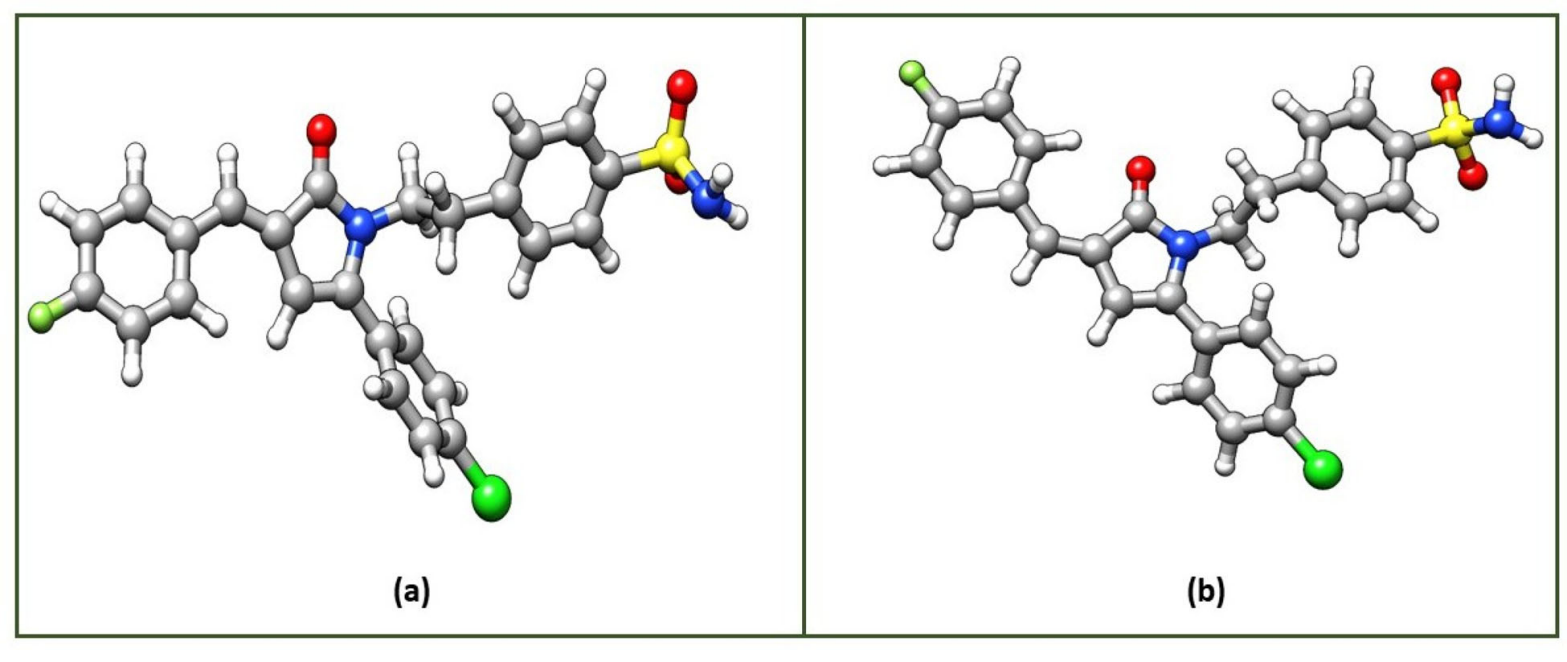
Optimized structures of (a) (3n-*E*) and (b) (3n-*Z*) at B3LYP/def2-TZVP D3BJ level of theory.

**Figure 4.**
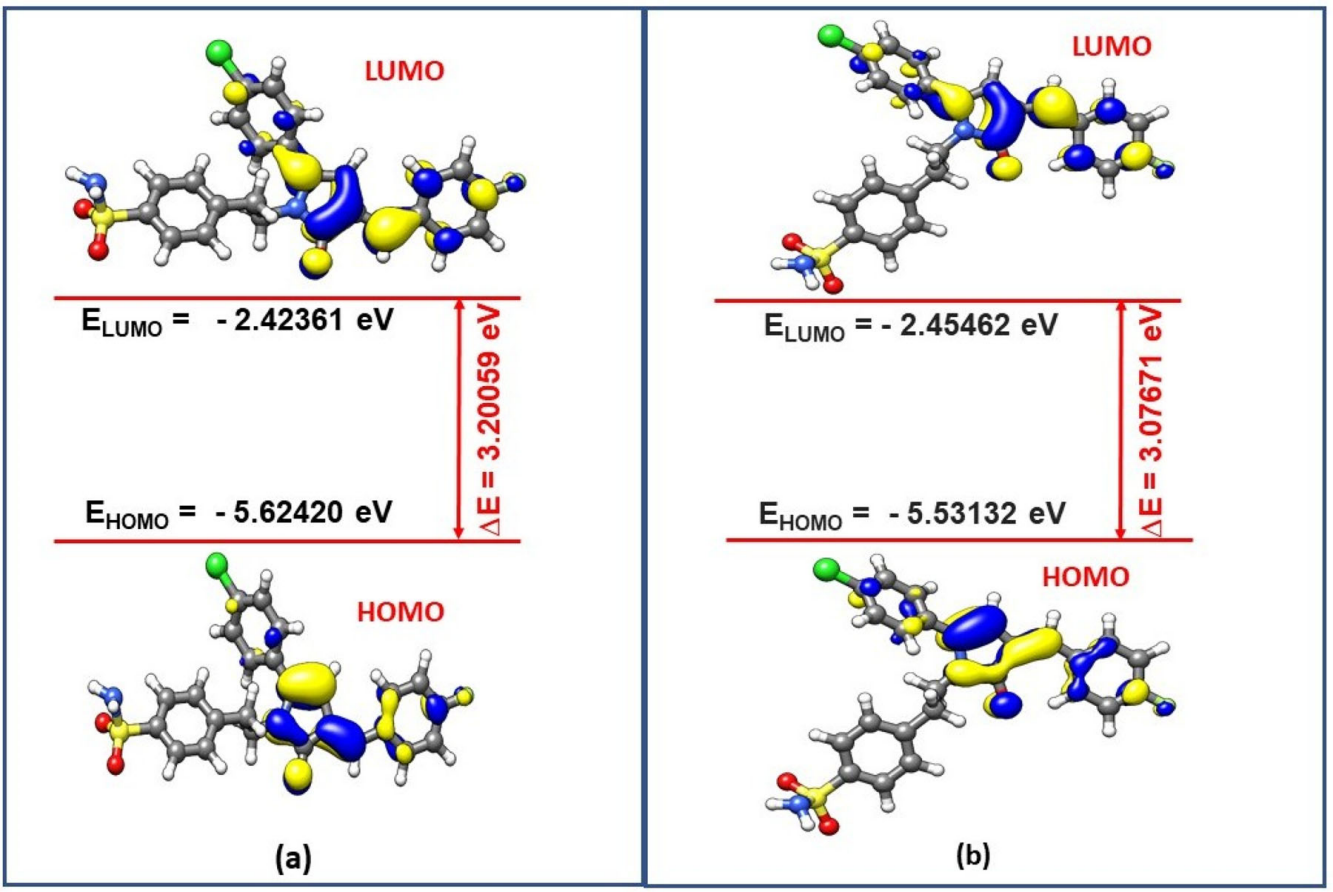
Frontier Molecular Orbitals (FMOs) of (a) Compound (3n-*E*) and (b) compound (3n-*Z*) optimized at B3LYP/def2-TZVP D3BJ level of theory.

Density Functional Theory (DFT) calculations were carried to gain more understanding about the stability of the **3n-*E*** and **3n-*Z*** isomers. Initial structures of both the isomers were visualised through UCSF Chimaera 1.12 Visualisation Package^44^ and then optimized using ORCA Program Package version 4.2^26^ at B3LYP/def2-TZVP D3BJ level of theory.^27–29^ The optimized structures and the corresponding optimized coordinates of both the isomers **3n-*E*** and **3n-*Z*** are shown in Figure 3(a,b) and Tables SX and SY of the Supporting Information (SI), respectively. After successful optimizations of both the isomers, the total energy of the **3n-*E*** isomer is −2255.653129872 Ha, while as total energy of the **3n-*Z*** isomer is found to be −2255.650017417Ha. The energy values clearly indicate that the stability of *E*-isomer over *Z*-isomer by −8.2 kJ mol^−1^. To get more insight in the stability of both the isomers, we also carried Frontier Molecular Orbital (FMO) analysis of these isomers at the same level of theory. FMO analysis has been found a useful tool in explaining reactivity and stability of various organic systems.^45–47^ In the FMO Analysis, the energy gap (ΔE) is calculated from the difference between the Energy of Lowest Unoccupied Molecular Orbital (E_LUMO_) and the Energy of Highest Occupied Molecular Orbital (E_HOMO_) according to the Koopmans’ relation ($\Delta E=\;E_{\mathrm{LUMO}}-E_{\mathrm{HOMO}}$). In Conceptual DFT terminology, this energy gap (ΔE) is also called as chemical hardness (η)^48^^,^^49^. Figure 4(a,b) presents the FMO plots of the optimised structures (**3n-*E*** and **3n-*Z*** isomers). As shown in Figure 4(a,b), the E_HOMO_ and E_LUMO_ of the *E*-isomer is found to be −5.62420 eV and −2.42361 eV respectively, whereas the E_HOMO_ and E_LUMO_ of the *Z*-isomer is found to be −5.53132 eV and −2.45462 eV. The computed energy gap (ΔE) or chemical hardness (η) for **3n-*E*** and **3n-*Z*** isomers is 3.20059 eV and 3.07671 eV respectively. Maximum hardness principle^50^ states that, “the more the value of ΔE or η, more stable is the system and less is the reactivity”. Therefore, it may be concluded that *E*-isomer is more stable *i.e.,* chemically more hard and hence less susceptible to attack by different chemical species in the reaction mixture, while as the *Z*-isomer is comparatively chemically less hard or more soft. It suggests that soft chemical species are more susceptible to electrophilic or nucleophilic attack, hence possibility of isolating *Z*-isomer is less as it might have been dissociated in the reaction mixture because of its soft nature and less stability as discussed.

## Conclusions

In the present study, 20 new hybrid molecules incorporating pyrrolone nucleus with ethyl benzenesulphonamide moiety **3a**-**t** were synthesised. Spectroscopic data and elemental analysis support the structures proposed for the synthesised compounds. These compounds along with acetazolamide, were tested as inhibitors of the physiologically relevant hCA isoforms, that is, hCAI, II, IX and XII. The potency of all compounds ranged from low nanomolar to high nanomolar. The compound **3b** behaved as more effective inhibitor against the human cytosolic isoforms hCA I and II having K_i_ values (368.7 and 81.4 nM) respectively, while **3n** behaved as more effective inhibitor against the membrane bound cancer related isozyme hCA IX and XII, having K_i_ values (41.3 and 39.1 nM) respectively. The results of ADMET studies provided a clear picture that all compounds have acceptable pharmacokinetic range and physicochemical characteristics. Density Functional Theory (DFT) calculations of **3n** were carried to gain more understanding about the stability of the *E* and *Z* isomers. The energy values clearly indicate the stability of *E-*isomer over *Z*-isomer by −8.2 kJ mol^−1^ which is further supported by Frontier Molecular Orbital (FMO) analysis of these isomers. Results of our investigation suggest another possible class of carbonic anhydrase inhibitors of medicinal importance, hence making these molecules as promising candidates for the discovery of new and effective inhibitor of carbonic anhydrase.

## Supplementary Material

Supplemental Material
