# Supplementary material for: Synthesis, biological evaluation and theoretical studies of (E)-1-(4-sulfamoyl-phenylethyl)-3-arylidene-5-aryl-1H-pyrrol-2(3H)-ones as human carbonic anhydrase inhibitors
Source: J Enzyme Inhib Med Chem. 2023 Mar 23;38(1):2189126. doi: 10.1080/14756366.2023.2189126 (PMC10038056; doi:10.1080/14756366.2023.2189126)
Supplement: Supplemental Material [file IENZ_A_2189126_SM7181.pdf]

Supporting Information

Synthesis, biological evaluation and theoretical studies of (*E*)-1-(4-sulfamoyl-phenylethyl)-3-arylidene-5-aryl-1H-pyrrol-2(3H)-ones as human carbonic anhydrase inhibitors.

Farhat Ramzan[1], Syed Ayaz Nabi[1], Mehak Saba Lone[1], Alessandro Bonardi[2], Aabid Hamid[3], Sameena Bano[4], Kalicharan Sharma[5], Syed Shafi[1], Mohammed Samim[1], Kalim Javed[1]*, Claudiu T.Supran[2]*.

1. Department of Chemistry, School of Chemical and Life Sciences, Jamia Hamdard (Hamdard University), New Delhi, 110062, India.

2. UniversitàdegliStudidiFirenze,Neurofarba Dept., Section of Pharmaceutical Chemistry, ViaU.Schiff6,50019SestoFiorentino(Florence),Italy.

3. Theoretical Chemistry Section, Chemistry Division, Bhabha Atomic Research Centre, Mumbai 400085, India.

4. Department of Computer Science and Engineering, School of Engineering Sciences and Technology, Jamia Hamdard, New Delhi, India-110062.

5. Department of Pharmaceutical Chemistry, Delhi Pharmaceutical Sciences and Research University Pushpvihar-110017, New Delhi, India,

Table of Contents

Copies of 1H NMR spectra of all compounds **(3a-t)**

2D-NMR (NOE of **3K**) (S1 and S2)

C13 NMR spectra **(3a, 3b, 3d, 3e, 3i, 3k, 3m, 3n)** of compounds

Mass spectra of all compounds **(3a-t)**

IR spectra of compounds **(3b,3h,3k,3m,3n,3s)**

DFT Data of compound **3n** (Table SX and SY)

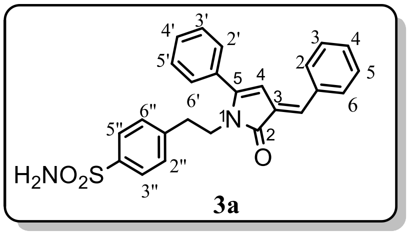

3a

SNS FARHAT R PKJ BBD

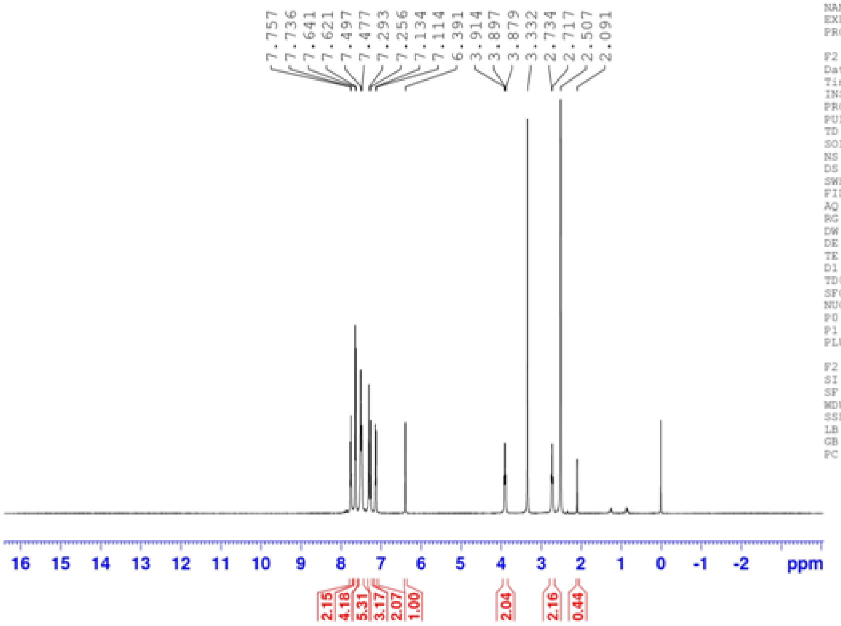


```
Current Data Parameters
NAME       SNS FARHAT R PKJ BBD
EXPNO                     5
PROCNO                    1

F2 - Acquisition Parameters
Date_              20220726
Time                  18.10 h
INSTRUM              Avance
PROBHD   Z8247_0054 (PH
PULPROG                zg30
TD                    65536
SOLVENT                DMSO
NS                       16
DS                        2
SWH                8196.722 Hz
FIDRES             0.250144 Hz
AQ                3.9976959 sec
RG                      101
DW                   61.000 usec
DE                    14.06 usec
TE                    298.1 K
D1           1.00000000 sec
TD0                       1
SFO1         400.1324708 MHz
NUC1                     1H
P0                     2.33 usec
P1                     7.00 usec
PLW1        14.19099998 W

F2 - Processing parameters
SI                    65536
SF           400.1300000 MHz
WDW                      EM
SSB                       0
LB                     0.30 Hz
GB                        0
PC                     1.00
```

SNS FARHAT R PKJ BBD

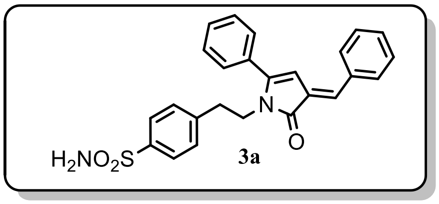

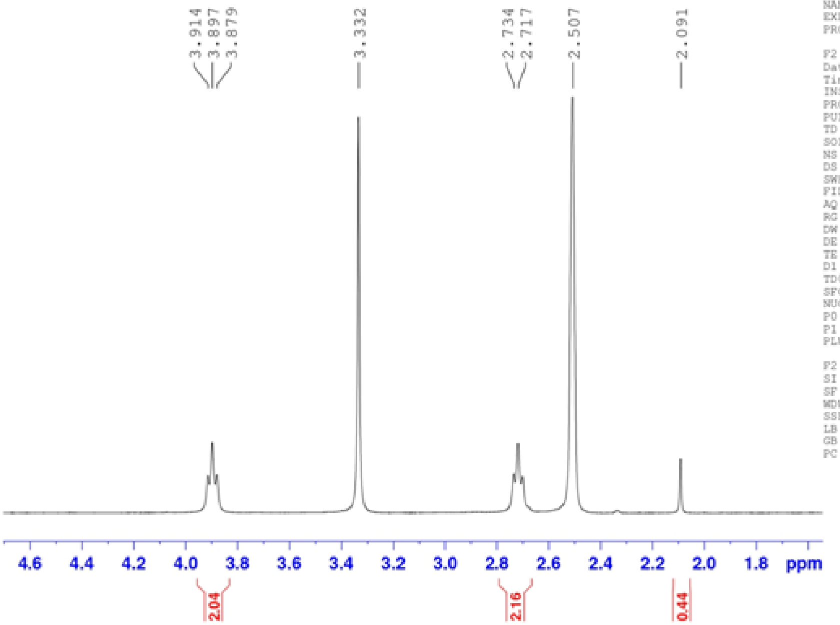


```
Current Data Parameters
NAME      SNS FARHAT R PKJ BBD
EXPNO                    5
PROCNO                   1

F2 - Acquisition Parameters
Date_             20220726
Time                 18.10 h
INSTRUM             Avance
PROBHD    Z8247_0054 (PH
PULPROG               zg30
TD                   65536
SOLVENT               DMSO
NS                      16
DS                       2
SWH               8196.722 Hz
FIDRES            0.250144 Hz
AQ               3.9976959 sec
RG                     101
DW                  61.000 usec
DE                   14.06 usec
TE                   298.1 K
D1              1.00000000 sec
TD0                      1
SFO1           400.1324708 MHz
NUC1                    1H
P0                    2.33 usec
P1                    7.00 usec
PLW1           14.19099998 W

F2 - Processing parameters
SI                   65536
SF             400.1300000 MHz
WDW                     EM
SSB                      0
LB                    0.30 Hz
GB                       0
PC                    1.00
```

SNS FARHAT R PKJ BBD

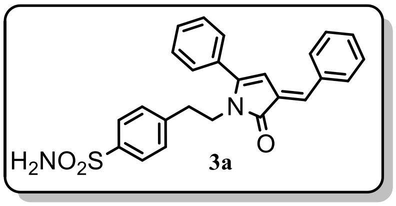

3a

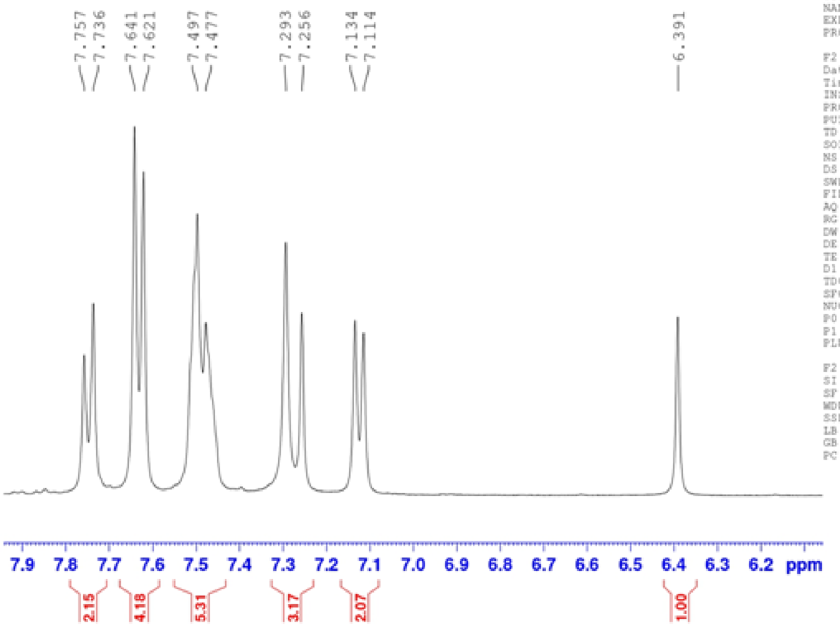

Current Data Parameters
NAME SNS FARHAT R PKJ BBD
EXPNO 5
PROCNO 1

F2 - Acquisition Parameters
Date_ 20220726
Time 18.10 h
INSTRUM Avance
PROBHD Z8247_0054 (PH
PULPROG zg30
TD 65536
SOLVENT DMSO
NS 16
DS 2
SWH 8196.722 Hz
FIDRES 0.250144 Hz
AQ 3.9976959 sec
RG 101
DW 61.000 usec
DE 14.06 usec
TE 298.1 K
D1 1.00000000 sec
TD0 1
SFO1 400.1324708 MHz
NUC1 1H
P0 2.33 usec
P1 7.00 usec
PLW1 14.19099998 W

F2 - Processing parameters
SI 65536
SF 400.1300000 MHz
WDW EM
SSB 0
LB 0.30 Hz
GB 0
PC 1.00

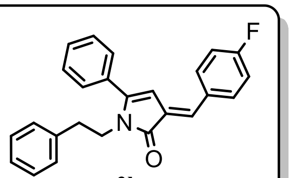

3b

SNS FARHAT R PKJ BF

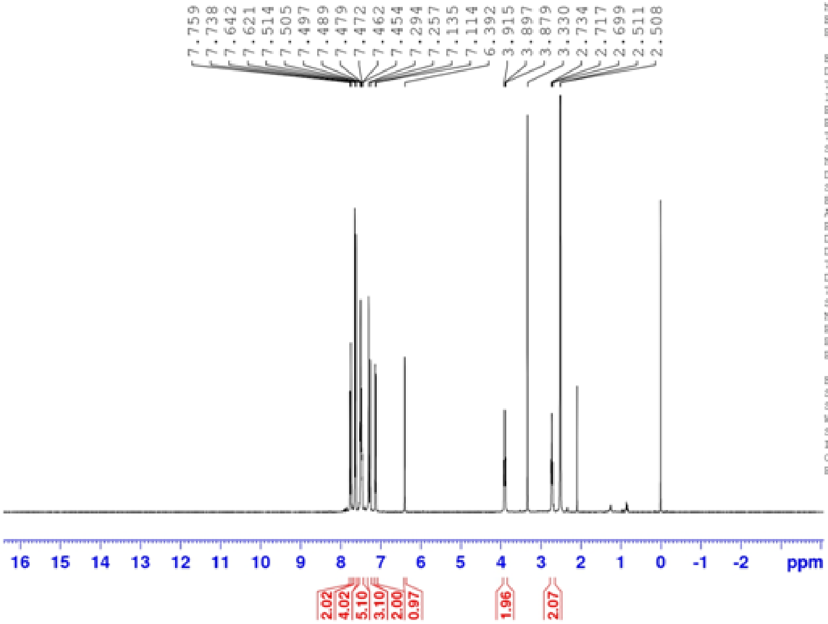

BRUKER

Current Data Parameters
NAME SNS FARHAT R PKJ BF
EXPNO 5
PROCNO 1

F2 - Acquisition Parameters
Date_ 20220708
Time 15.45 h
INSTRUM Avance
PROBHD Z8247_0054 (PH
PULPROG zg30
TD 65536
SOLVENT DMSO
NS 16
DS 2
SWH 8196.722 Hz
FIDRES 0.250144 Hz
AQ 3.9976959 sec
RG 101
DW 61.000 usec
DE 14.06 usec
TE 298.3 K
D1 1.00000000 sec
TD0 1
SFO1 400.1324708 MHz
NUC1 1H
P0 2.33 usec
P1 7.00 usec
PLW1 14.19099998 W

F2 - Processing parameters
SI 65536
SF 400.1300000 MHz
WDW EM
SSB 0
LB 0.30 Hz
GB 0
PC 1.00

SNS FARHAT R PKJ BF

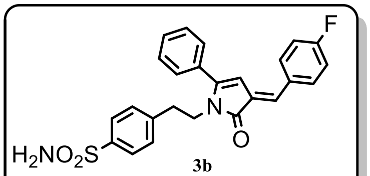

3b

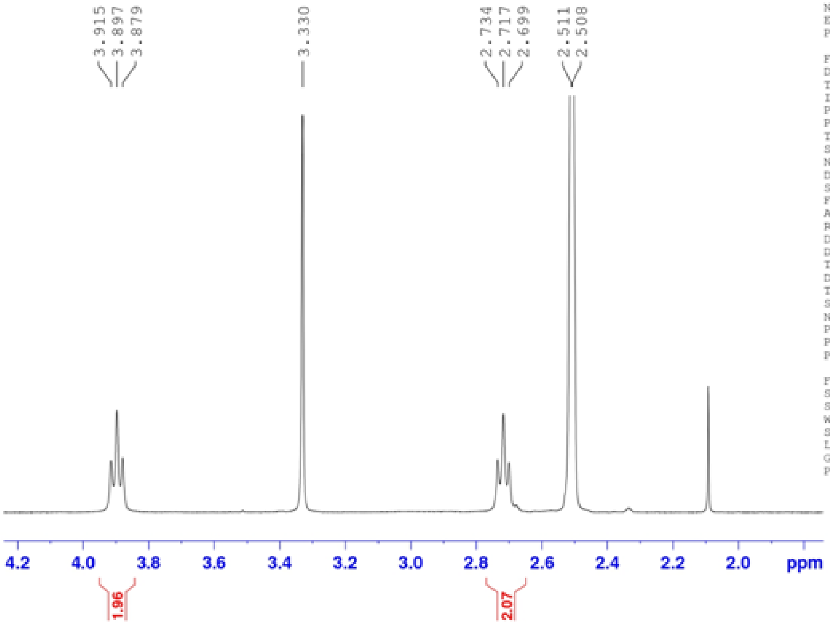

BRUKER

Current Data Parameters
NAME SNS FARHAT R PKJ BF
EXPNO 5
PROCNO 1

F2 - Acquisition Parameters
Date_ 20220708
Time 15.45 h
INSTRUM Avance
PROBHD Z8247_0054 (PH
PULPROG zg30
TD 65536
SOLVENT DMSO
NS 16
DS 2
SWH 8196.722 Hz
FIDRES 0.250144 Hz
AQ 3.9976959 sec
RG 101
DW 61.000 usec
DE 14.06 usec
TE 298.3 K
D1 1.00000000 sec
TD0 1
SFO1 400.1324708 MHz
NUC1 1H
P0 2.33 usec
P1 7.00 usec
PLW1 14.19099998 W

F2 - Processing parameters
SI 65536
SF 400.1300000 MHz
WDW EM
SSB 0
LB 0.30 Hz
GB 0
PC 1.00

SNS FARHAT R PKJ BF

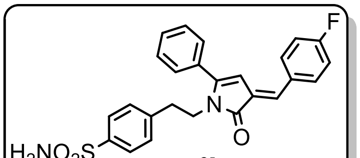

3b

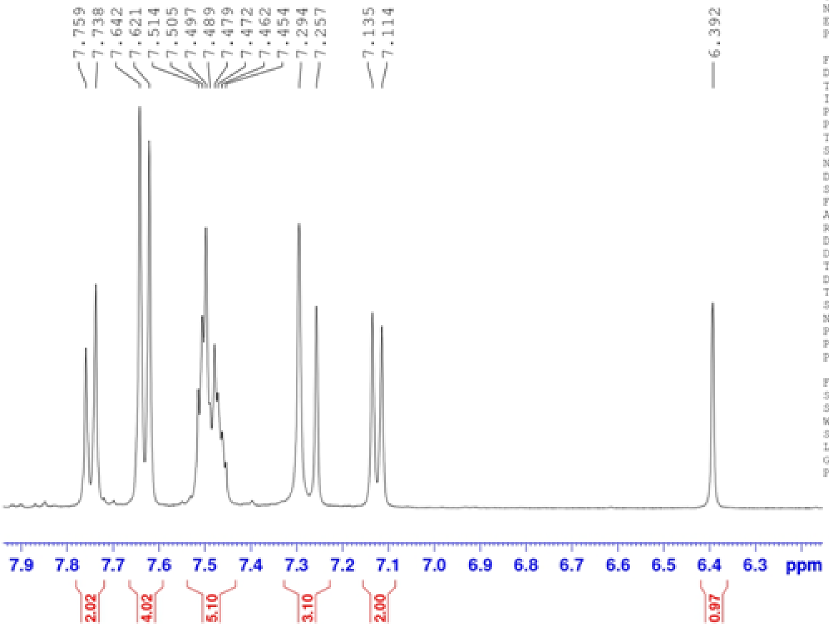

Current Data Parameters
NAME SNS FARHAT R PKJ BF
EXPNO 5
PROCNO 1

F2 - Acquisition Parameters
Date_ 20220708
Time 15.45 h
INSTRUM Avance
PROBHD Z8247_0054 (PH
PULPROG zg30
TD 65536
SOLVENT DMSO
NS 16
DS 2
SWH 8196.722 Hz
FIDRES 0.250144 Hz
AQ 3.9976959 sec
RG 101
DW 61.000 usec
DE 14.06 usec
TE 298.3 K
D1 1.00000000 sec
TD0 1
SFO1 400.1324708 MHz
NUC1 1H
P0 2.33 usec
P1 7.00 usec
PLW1 14.19099998 W

F2 - Processing parameters
SI 65536
SF 400.1300000 MHz
WDW EM
SSB 0
LB 0.30 Hz
GB 0
PC 1.00

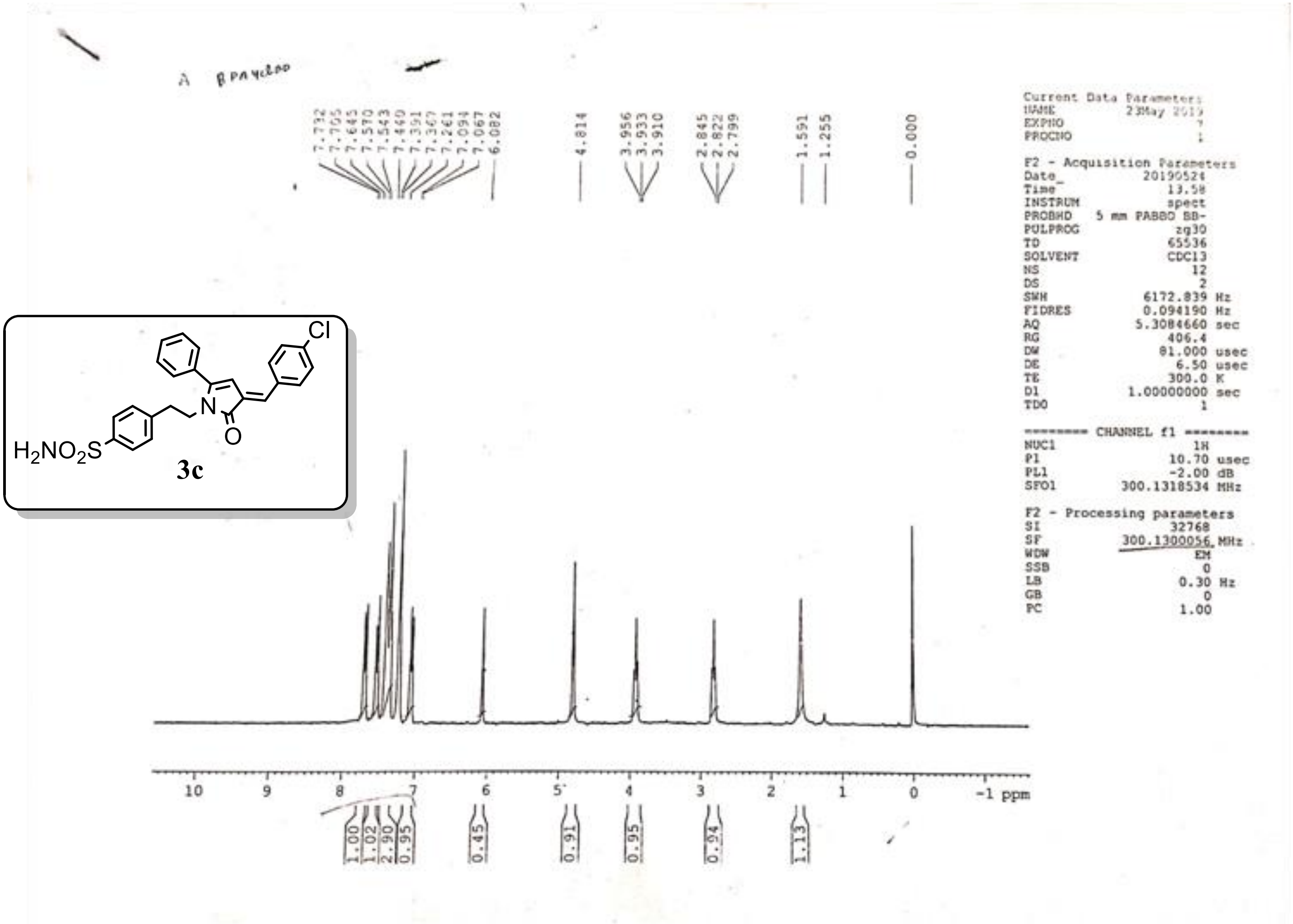

Scanned with CamScanner

SNS FARHAT R PKJ BBr

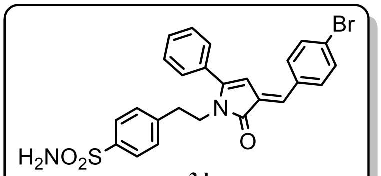

3d

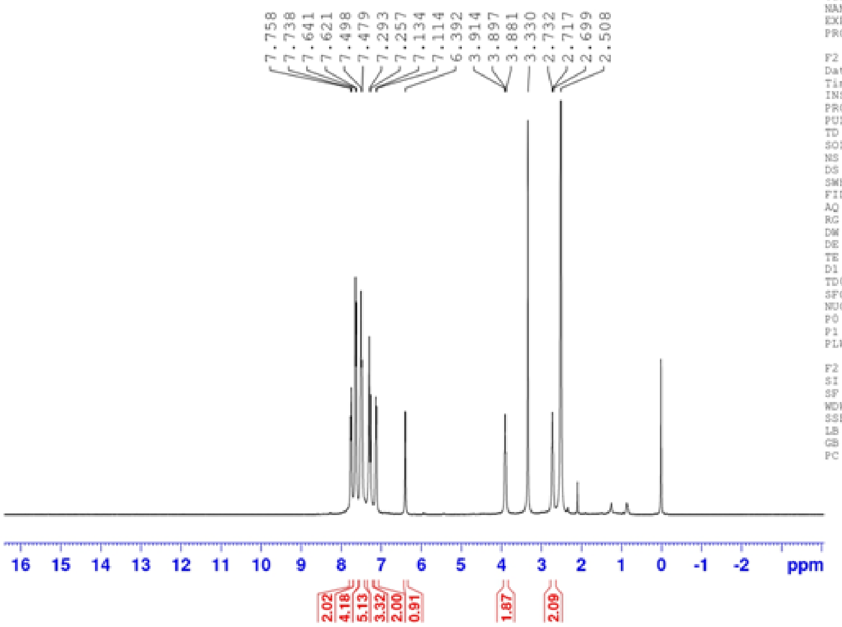

Current Data Parameters
NAME SNS FARHAT R PKJ BBr
EXPNO 5
PROCNO 1

F2 - Acquisition Parameters
Date_ 20220723
Time 17.24 h
INSTRUM Avance
PROBHD Z8247_0054 (PH
PULPROG zg30
TD 65536
SOLVENT DMSO
NS 16
DS 2
SWH 8196.722 Hz
FIDRES 0.250144 Hz
AQ 3.9976959 sec
RG 101
DW 61.000 usec
DE 14.06 usec
TE 298.0 K
D1 1.00000000 sec
TD0 1
SFO1 400.1324708 MHz
NUC1 1H
P0 2.33 usec
P1 7.00 usec
PLW1 14.19099998 W

F2 - Processing parameters
SI 65536
SF 400.1300000 MHz
WDW EM
SSB 0
LB 0.30 Hz
GB 0
PC 1.00

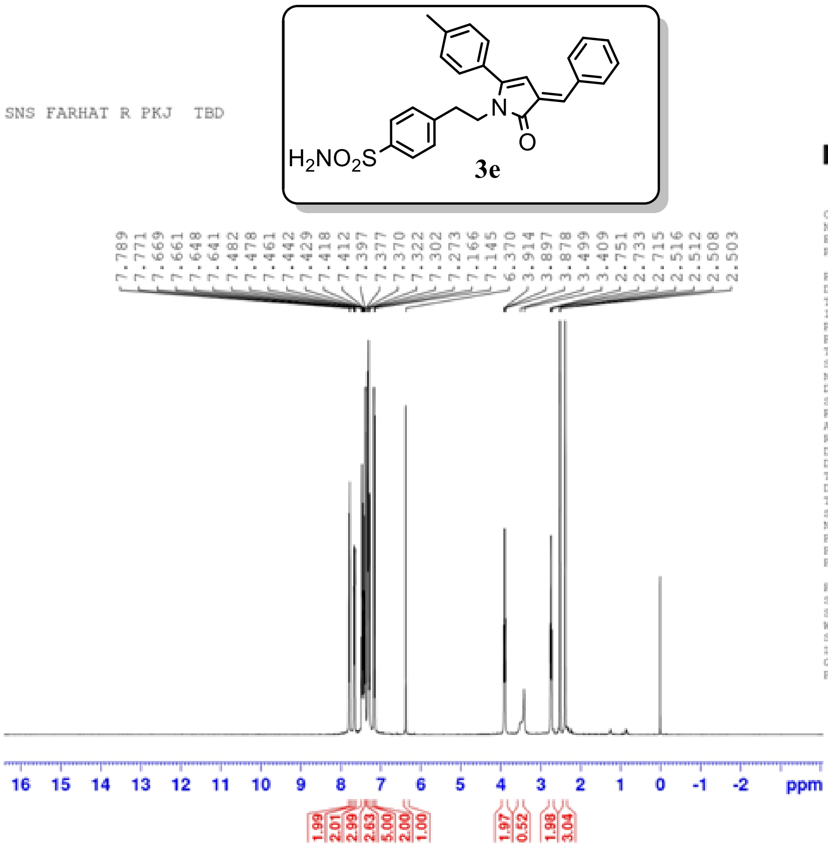

Current Data Parameters
NAME SNS FARHAT R PKJ TBD
EXPNO 1
PROCNO 1

F2 - Acquisition Parameters
Date_ 20220723
Time 18.59 h
INSTRUM Avance
PROBHD Z8247_0054 (PH
PULPROG zg30
TD 65536
SOLVENT DMSO
NS 16
DS 2
SWH 8196.722 Hz
FIDRES 0.250144 Hz
AQ 3.9976959 sec
RG 101
DW 61.000 usec
DE 14.06 usec
TE 298.1 K
D1 1.00000000 sec
TD0 1
SFO1 400.1324708 MHz
NUC1 1H
P0 2.33 usec
P1 7.00 usec
PLW1 14.19099998 W

F2 - Processing parameters
SI 65536
SF 400.1300000 MHz
WDW EM
SSB 0
LB 0.30 Hz
GB 0
PC 1.00

SNS FARHAT R PKJ TBD

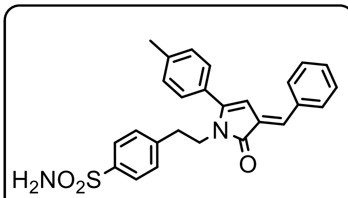

3e

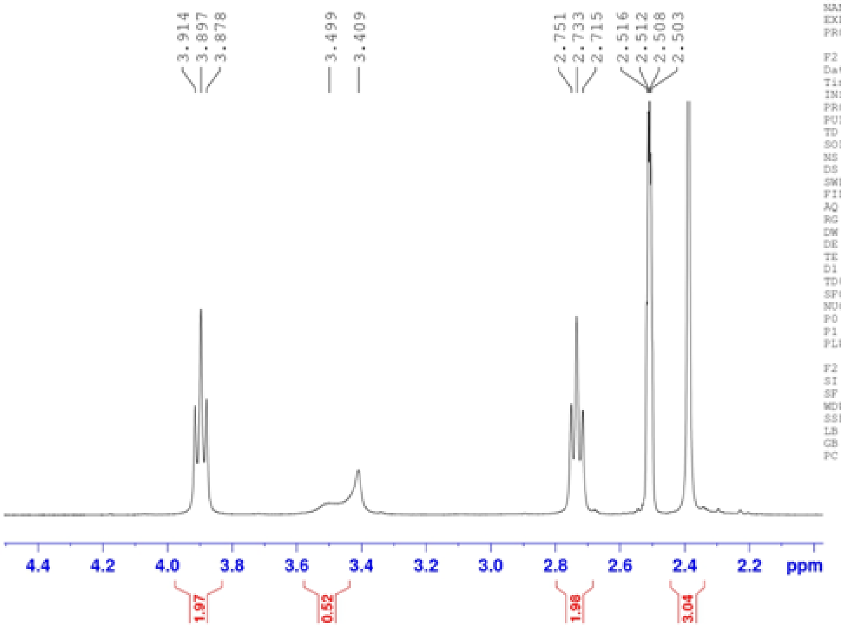

BRUKER

```
Current Data Parameters
NAME      SNS FARHAT R PKJ TBD
EXPNO                    1
PROCNO                   1

F2 - Acquisition Parameters
Date_             20220723
Time                 18.59 h
INSTRUM             Avance
PROBHD   Z8247_0054 (PH
PULPROG               zg30
TD                   65536
SOLVENT               DMSO
NS                      16
DS                       2
SWH               8196.722 Hz
FIDRES            0.250144 Hz
AQ               3.9976959 sec
RG                     101
DW                  61.000 usec
DE                   14.06 usec
TE                   298.1 K
D1              1.00000000 sec
TD0                      1
SFO1           400.1324708 MHz
NUC1                    1H
P0                    2.33 usec
P1                    7.00 usec
PLW1           14.19099998 W

F2 - Processing parameters
SI                   65536
SF             400.1300000 MHz
WDW                     EM
SSB                      0
LB                    0.30 Hz
GB                       0
PC                    1.00
```

SNS FARHAT R PKJ TBD

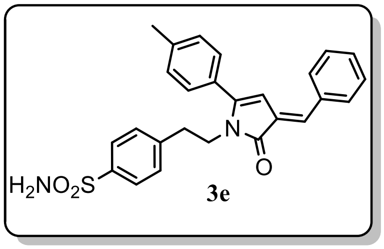

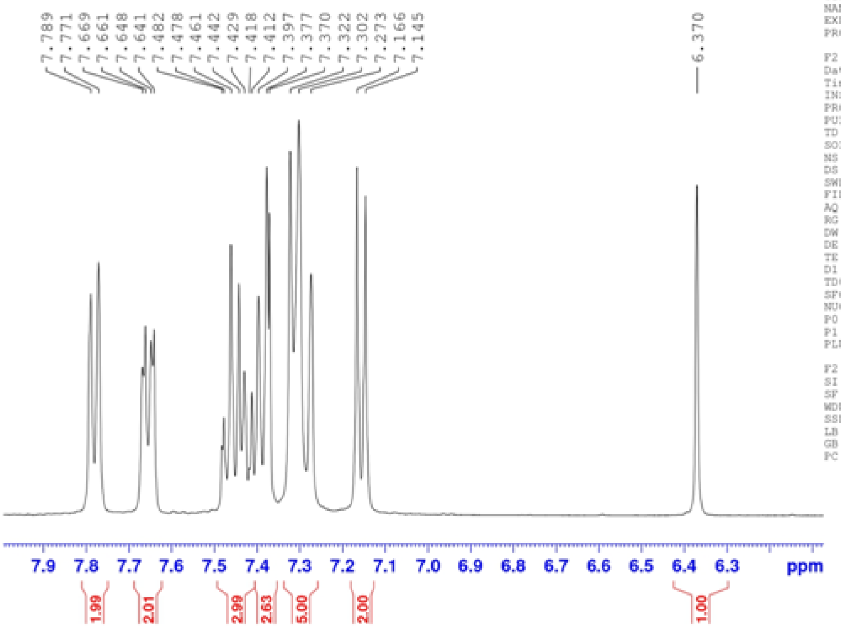


```
Current Data Parameters
NAME      SNS FARHAT R PKJ TBD
EXPNO                     1
PROCNO                    1

F2 - Acquisition Parameters
Date_              20220723
Time                  18.59 h
INSTRUM              Avance
PROBHD   Z8247_0054 (PH
PULPROG                zg30
TD                    65536
SOLVENT                DMSO
NS                       16
DS                        2
SWH                8196.722 Hz
FIDRES             0.250144 Hz
AQ                3.9976959 sec
RG                      101
DW                   61.000 usec
DE                    14.06 usec
TE                    298.1 K
D1               1.00000000 sec
TD0                       1
SFO1            400.1324708 MHz
NUC1                     1H
P0                     2.33 usec
P1                     7.00 usec
PLW1            14.19099998 W

F2 - Processing parameters
SI                    65536
SF              400.1300000 MHz
WDW                      EM
SSB                       0
LB                     0.30 Hz
GB                        0
PC                     1.00
```

p6

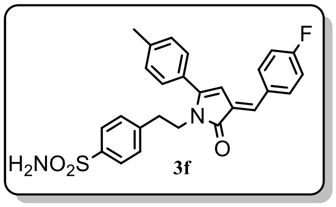

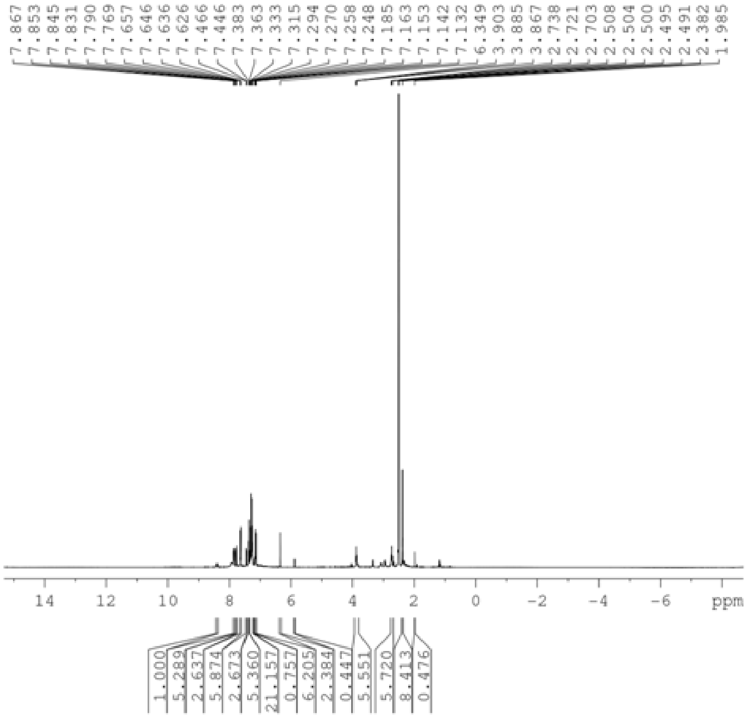

Current Data Parameters
NAME 03092021-mehgul1
EXPNO 9
PROCNO 1

F2 - Acquisition Parameters
Date_ 20210903
Time 7.58
INSTRUM spect
PROBHD 5 mm PABBO BB-
PULPROG zgpr
TD 28844
SOLVENT DMSO
NS 128
DS 0
SWH 9615.385 Hz
FIDRES 0.333358 Hz
AQ 1.4998879 sec
RG 203
DW 52.000 usec
DE 6.50 usec
TE 298.0 K
D1 2.00000000 sec
D12 0.00002000 sec
TD0 1

======== CHANNEL f1 ========
NUC1 1H
P1 13.50 usec
PL1 -3.00 dB
PL9 48.37 dB
PL1W 13.42244530 W
PL9W 0.00009791 W
SFO1 400.2313328 MHz

F2 - Processing parameters
SI 32768
SF 400.2300061 MHz
WDW EM
SSB 0
LB 0.30 Hz

p6

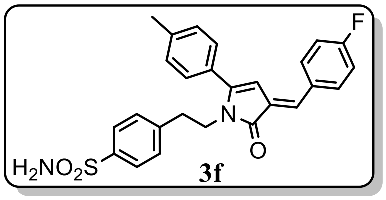

3f

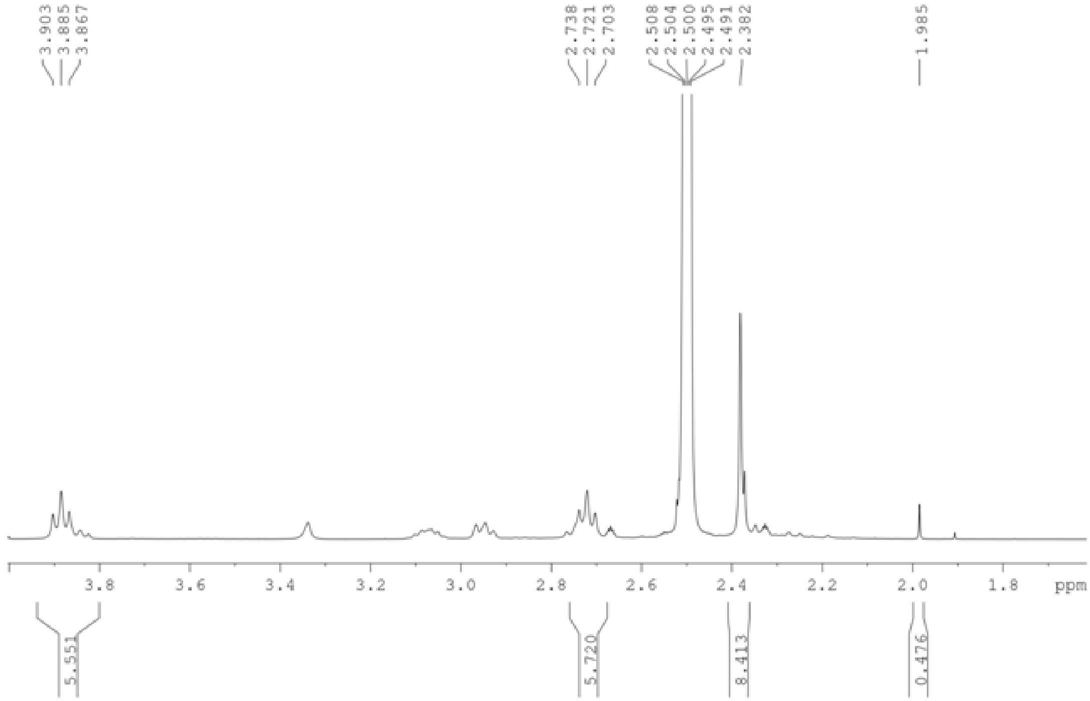

3.903
3.885
3.867
2.738
2.721
2.703
2.508
2.504
2.500
2.495
2.491
2.382
1.985
3.8
3.6
3.4
3.2
3.0
2.8
2.6
2.4
2.2
2.0
1.8
ppm
5.551
5.720
8.413
0.476

p1

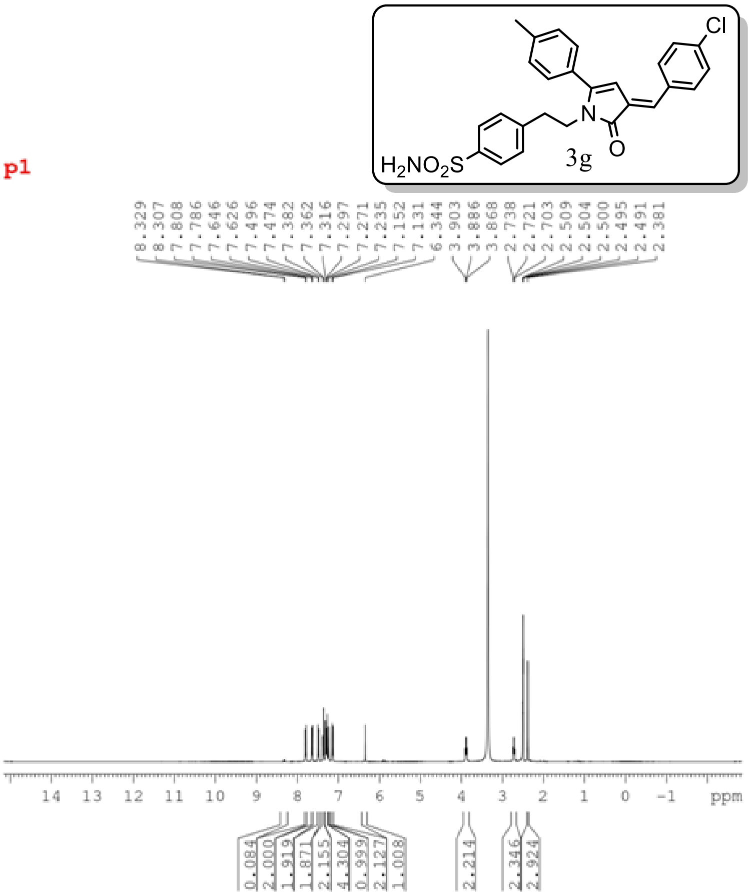


```
Current Data Parameters
NAME        03092021-mehgul1
EXPNO                      3
PROCNO                     1

F2 - Acquisition Parameters
Date_               20210903
Time                    6.47
INSTRUM                spect
PROBHD    5 mm PABBO BB-
PULPROG                   zg
TD                     28844
SOLVENT                 DMSO
NS                        32
DS                         0
SWH                 7211.539 Hz
FIDRES              0.250019 Hz
AQ                 1.9998506 sec
RG                       144
DW                    69.333 usec
DE                      6.50 usec
TE                     298.0 K
D1                2.00000000 sec
TD0                        1

======== CHANNEL f1 ========
NUC1                      1H
P1                     13.50 usec
PL1                    -3.00 dB
PL1W             13.42244530 W
SFO1             400.2324714 MHz

F2 - Processing parameters
SI                     32768
SF               400.2300061 MHz
WDW                       EM
SSB                        0
LB                      0.30 Hz
GB                         0
PC                      1.00
```

p1

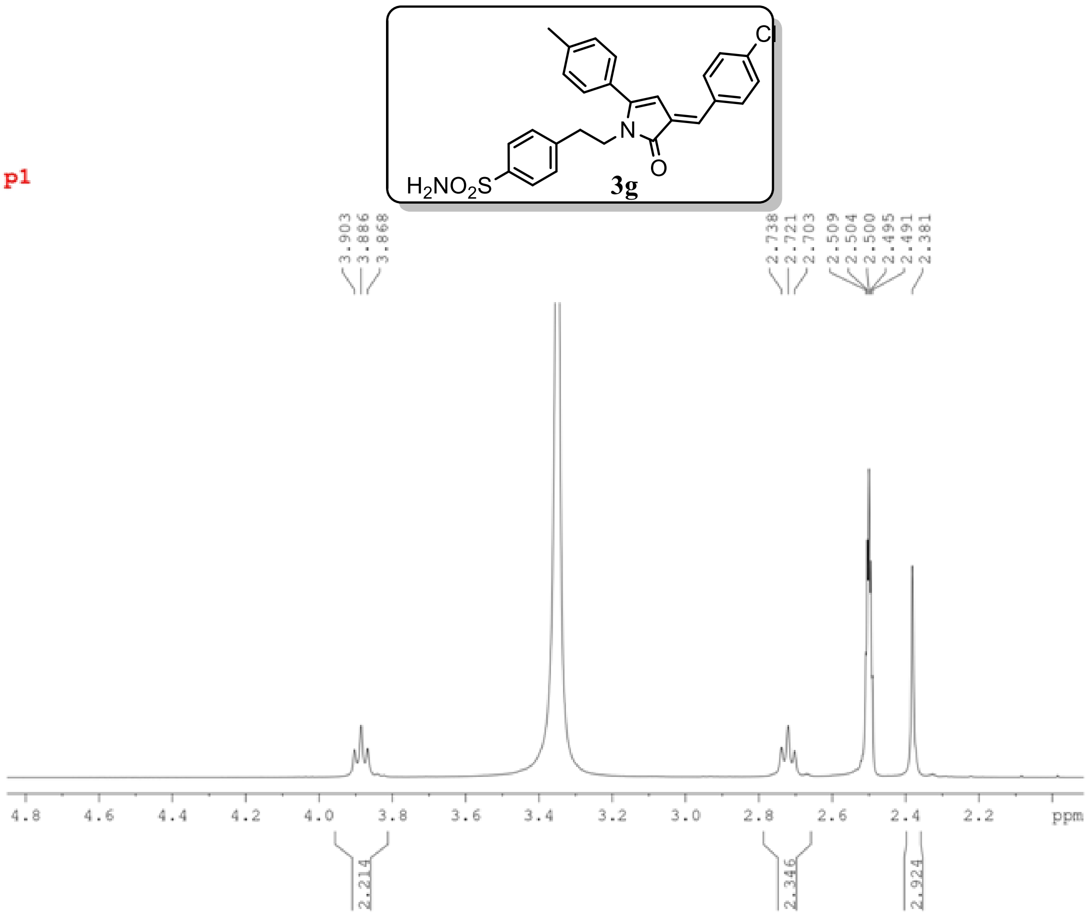

H2NO2S
N
O
Cl
3g
3.903
3.886
3.868
2.738
2.721
2.703
2.509
2.504
2.500
2.495
2.491
2.381
4.8
4.6
4.4
4.2
4.0
3.8
3.6
3.4
3.2
3.0
2.8
2.6
2.4
2.2
ppm
2.214
2.346
2.924

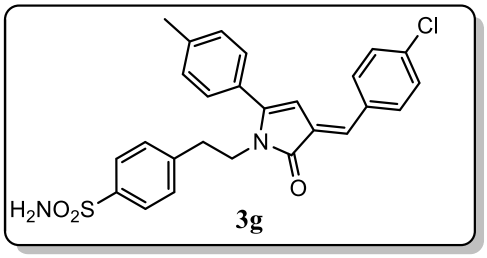
Cl
N
O
H2NO2S

3g

p1

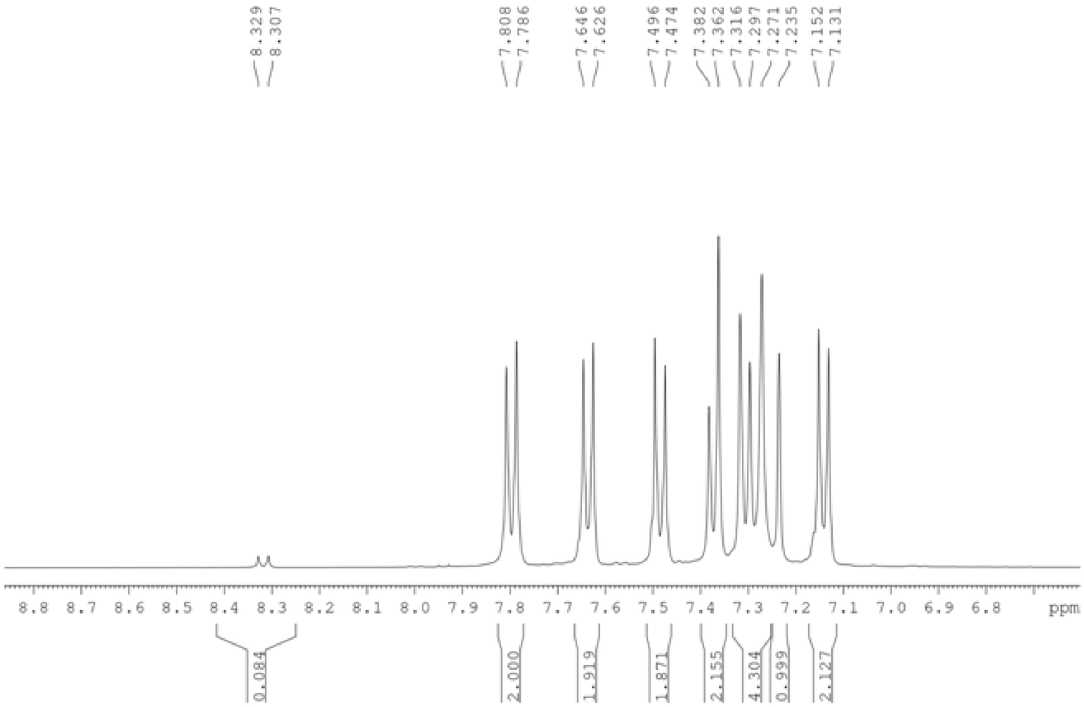
8.329
8.307
7.808
7.786
7.646
7.626
7.496
7.474
7.382
7.362
7.316
7.297
7.271
7.235
7.152
7.131
8.8 8.7 8.6 8.5 8.4 8.3 8.2 8.1 8.0 7.9 7.8 7.7 7.6 7.5 7.4 7.3 7.2 7.1 7.0 6.9 6.8 ppm
0.084
2.000
1.919
1.871
2.155
4.304
0.999
2.127

p2

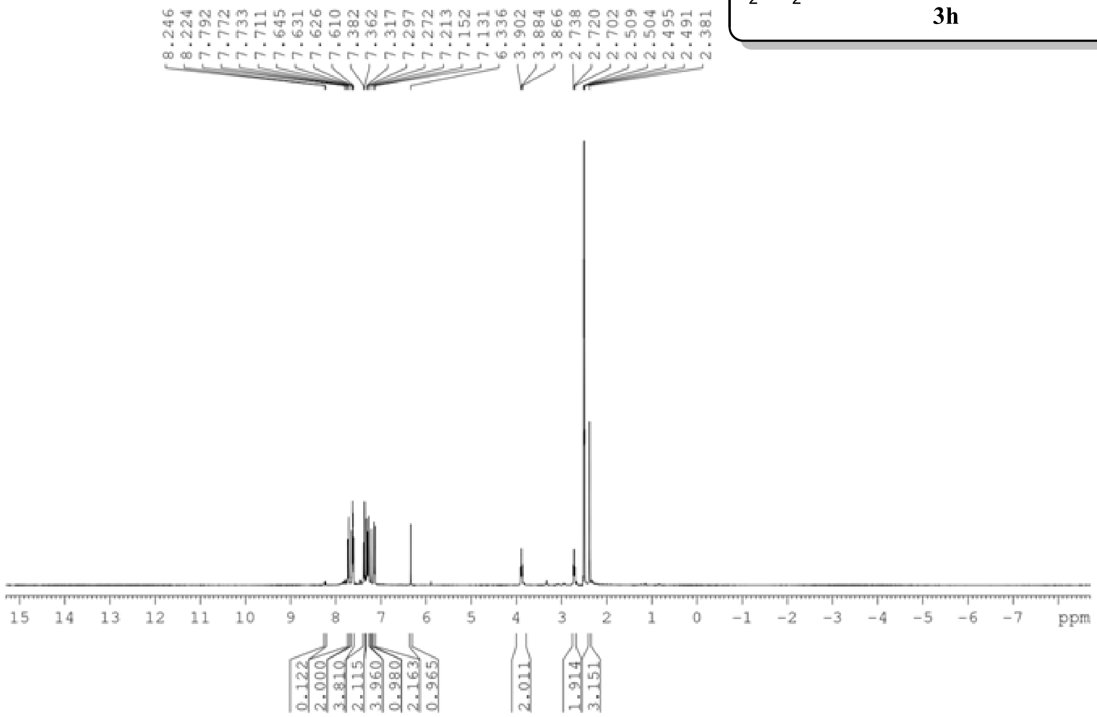
8.246
8.224
7.792
7.772
7.733
7.711
7.645
7.631
7.626
7.610
7.382
7.362
7.317
7.297
7.272
7.213
7.152
7.131
6.336
3.902
3.884
3.866
2.738
2.720
2.702
2.509
2.504
2.495
2.491
2.381
15 14 13 12 11 10 9 8 7 6 5 4 3 2 1 0 -1 -2 -3 -4 -5 -6 -7 ppm
0.122
2.000
3.810
2.115
3.960
0.980
2.163
0.965
2.011
1.914
3.151

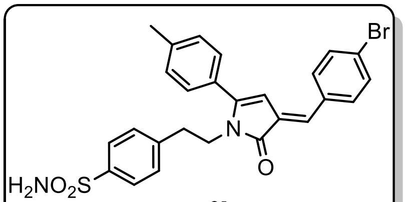
Br
N
O
H_2NO_2S

3h

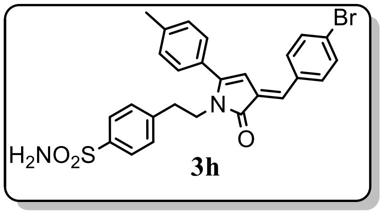
Br
H2NO2S
N
O

3h

p2

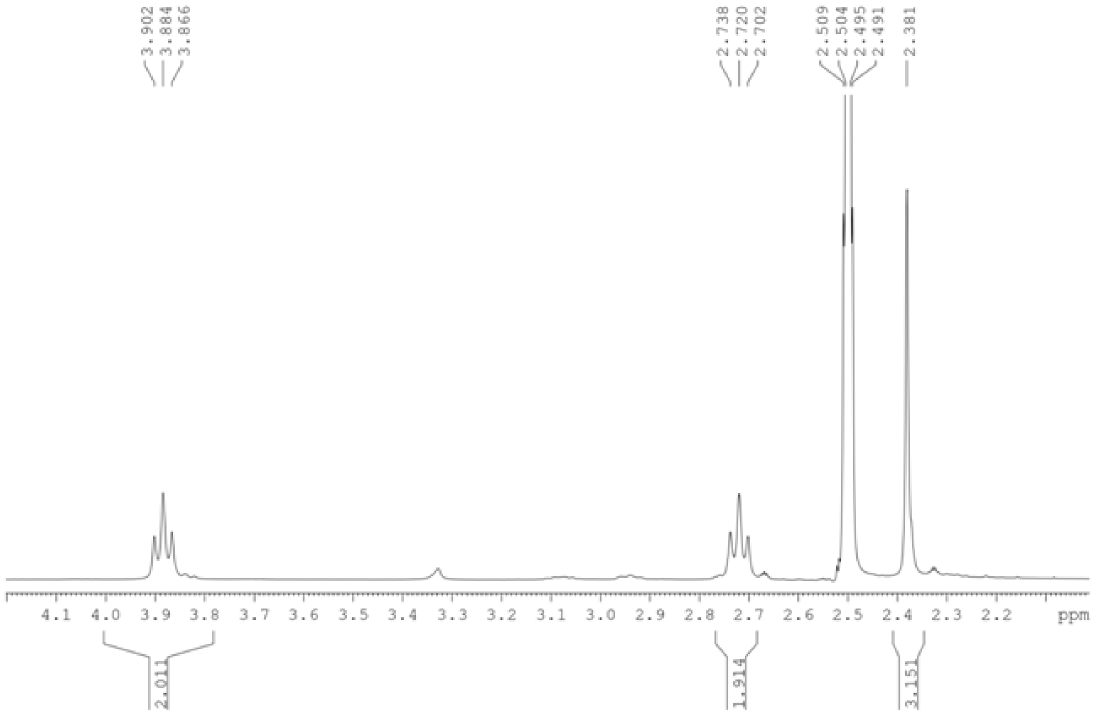
3.902
3.884
3.866
2.738
2.720
2.702
2.509
2.504
2.495
2.491
2.381
4.1 4.0 3.9 3.8 3.7 3.6 3.5 3.4 3.3 3.2 3.1 3.0 2.9 2.8 2.7 2.6 2.5 2.4 2.3 2.2 ppm
2.011
1.914
3.151

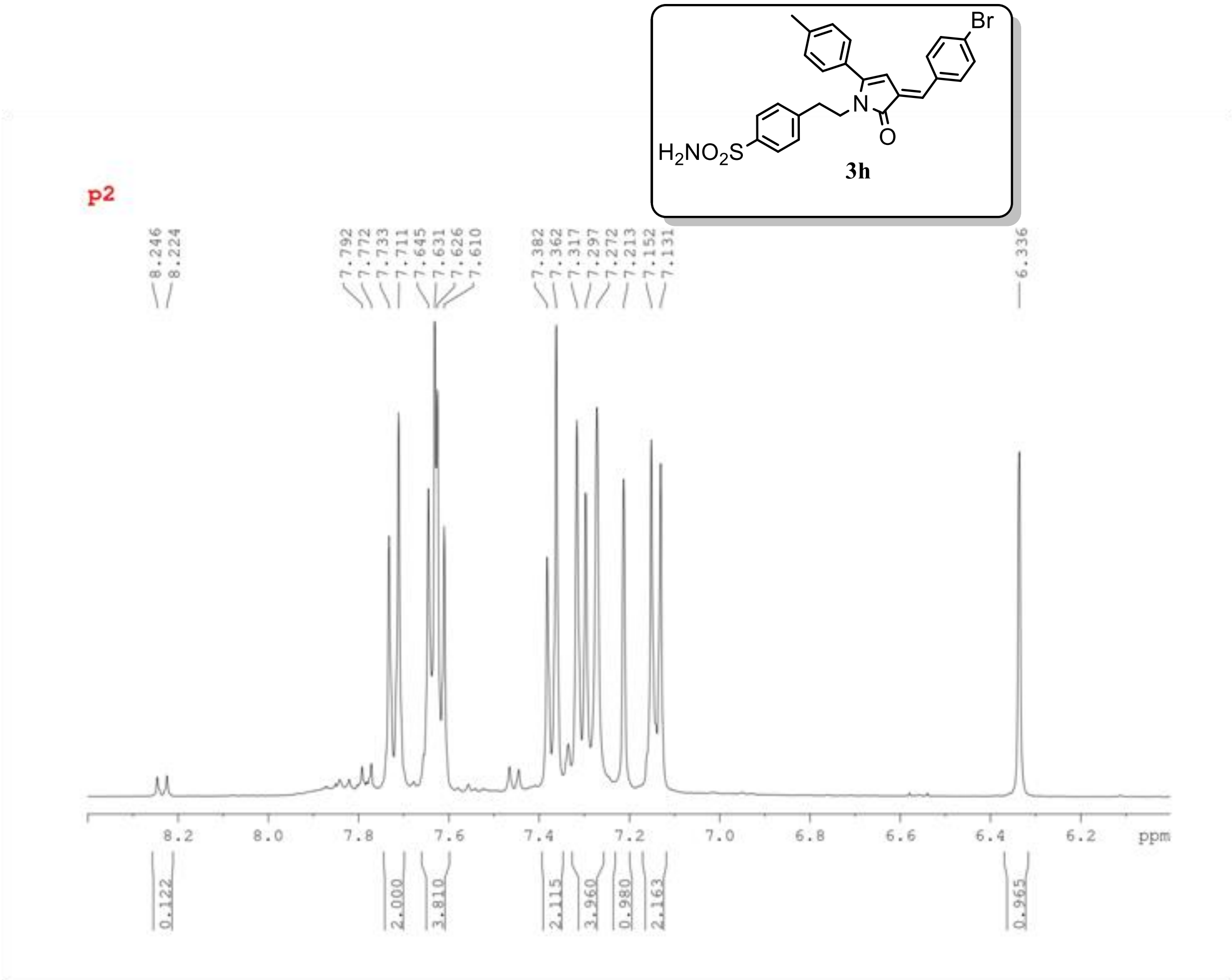

Br
H_2NO_2S
N
O
3h
p2
8.246
8.224
7.792
7.772
7.733
7.711
7.645
7.631
7.626
7.610
7.382
7.362
7.317
7.297
7.272
7.213
7.152
7.131
6.336
8.2
8.0
7.8
7.6
7.4
7.2
7.0
6.8
6.6
6.4
6.2
ppm
0.122
2.000
3.810
2.115
3.960
0.980
2.163
0.965

SNS FARHAT R PKJ BB

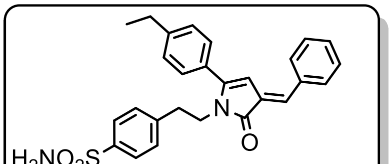

3i

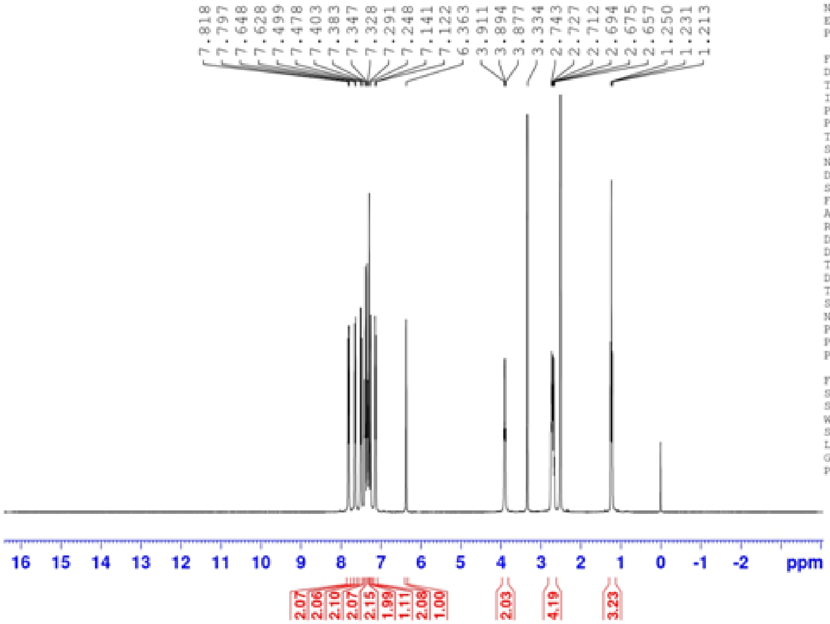


```
Current Data Parameters
NAME      SNS FARHAT R PKJ BB
EXPNO                      4
PROCNO                     1

F2 - Acquisition Parameters
Date_               20220628
Time                   16.29 h
INSTRUM               Avance
PROBHD   Z8247_0054 (PH
PULPROG                 zg30
TD                     65536
SOLVENT                 DMSO
NS                        16
DS                         2
SWH                 8196.722 Hz
FIDRES              0.250144 Hz
AQ                 3.9976959 sec
RG                       101
DW                    61.000 usec
DE                     14.06 usec
TE                     299.6 K
D1                1.00000000 sec
TD0                        1
SFO1             400.1324708 MHz
NUC1                      1H
P0                      2.33 usec
P1                      7.00 usec
PLW1             14.19099998 W

F2 - Processing parameters
SI                     65536
SF               400.1300000 MHz
WDW                       EM
SSB                        0
LB                      0.30 Hz
GB                         0
PC                      1.00
```

SNS FARHAT R PKJ BB

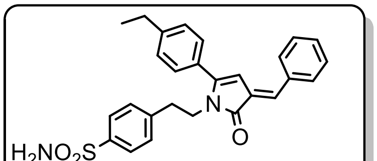

3i

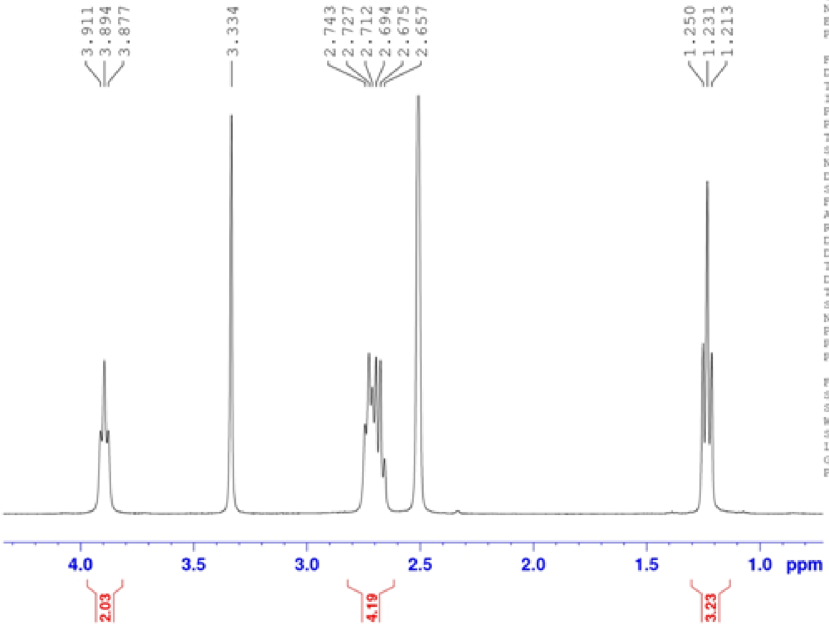


```
Current Data Parameters
NAME       SNS FARHAT R PKJ BB
EXPNO                   4
PROCNO                  1

F2 - Acquisition Parameters
Date_            20220628
Time                16.29 h
INSTRUM            Avance
PROBHD   Z8247_0054 (PH
PULPROG              zg30
TD                  65536
SOLVENT              DMSO
NS                     16
DS                      2
SWH              8196.722 Hz
FIDRES           0.250144 Hz
AQ              3.9976959 sec
RG                    101
DW                 61.000 usec
DE                  14.06 usec
TE                  299.6 K
D1             1.00000000 sec
TD0                     1
SFO1          400.1324708 MHz
NUC1                   1H
P0                   2.33 usec
P1                   7.00 usec
PLW1          14.19099998 W

F2 - Processing parameters
SI                  65536
SF            400.1300000 MHz
WDW                    EM
SSB                     0
LB                   0.30 Hz
GB                      0
PC                   1.00
```

SNS FARHAT R PKJ BB

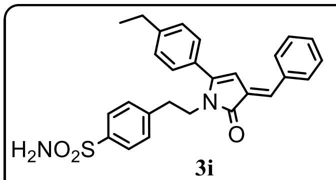

3i

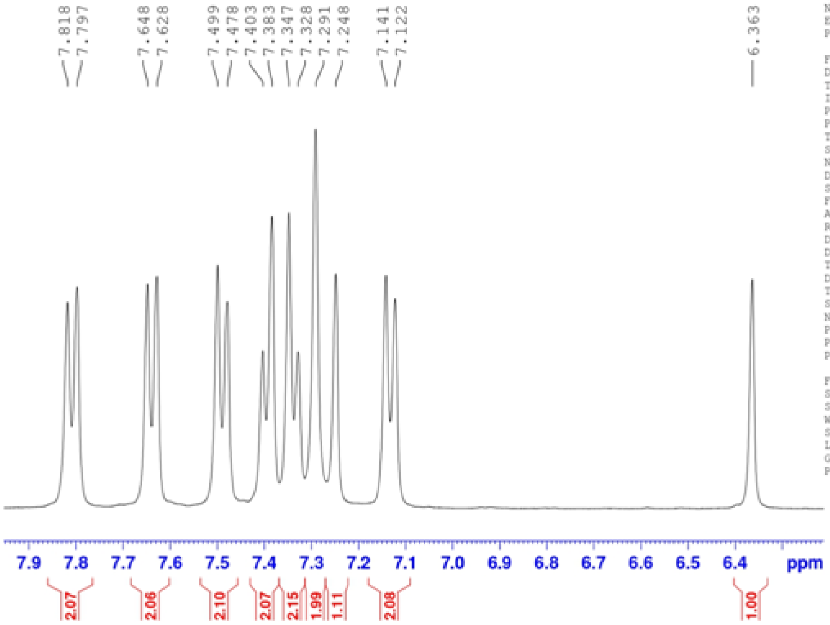


```
Current Data Parameters
NAME       SNS FARHAT R PKJ BB
EXPNO                 4
PROCNO                1

F2 - Acquisition Parameters
Date_           20220628
Time               16.29 h
INSTRUM           Avance
PROBHD   Z8247_0054 (PH
PULPROG             zg30
TD                 65536
SOLVENT             DMSO
NS                    16
DS                     2
SWH             8196.722 Hz
FIDRES          0.250144 Hz
AQ             3.9976959 sec
RG                   101
DW                61.000 usec
DE                 14.06 usec
TE                 299.6 K
D1            1.00000000 sec
TD0                    1
SFO1         400.1324708 MHz
NUC1                  1H
P0                  2.33 usec
P1                  7.00 usec
PLW1         14.19099998 W

F2 - Processing parameters
SI                 65536
SF           400.1300000 MHz
WDW                   EM
SSB                    0
LB                  0.30 Hz
GB                     0
PC                  1.00
```

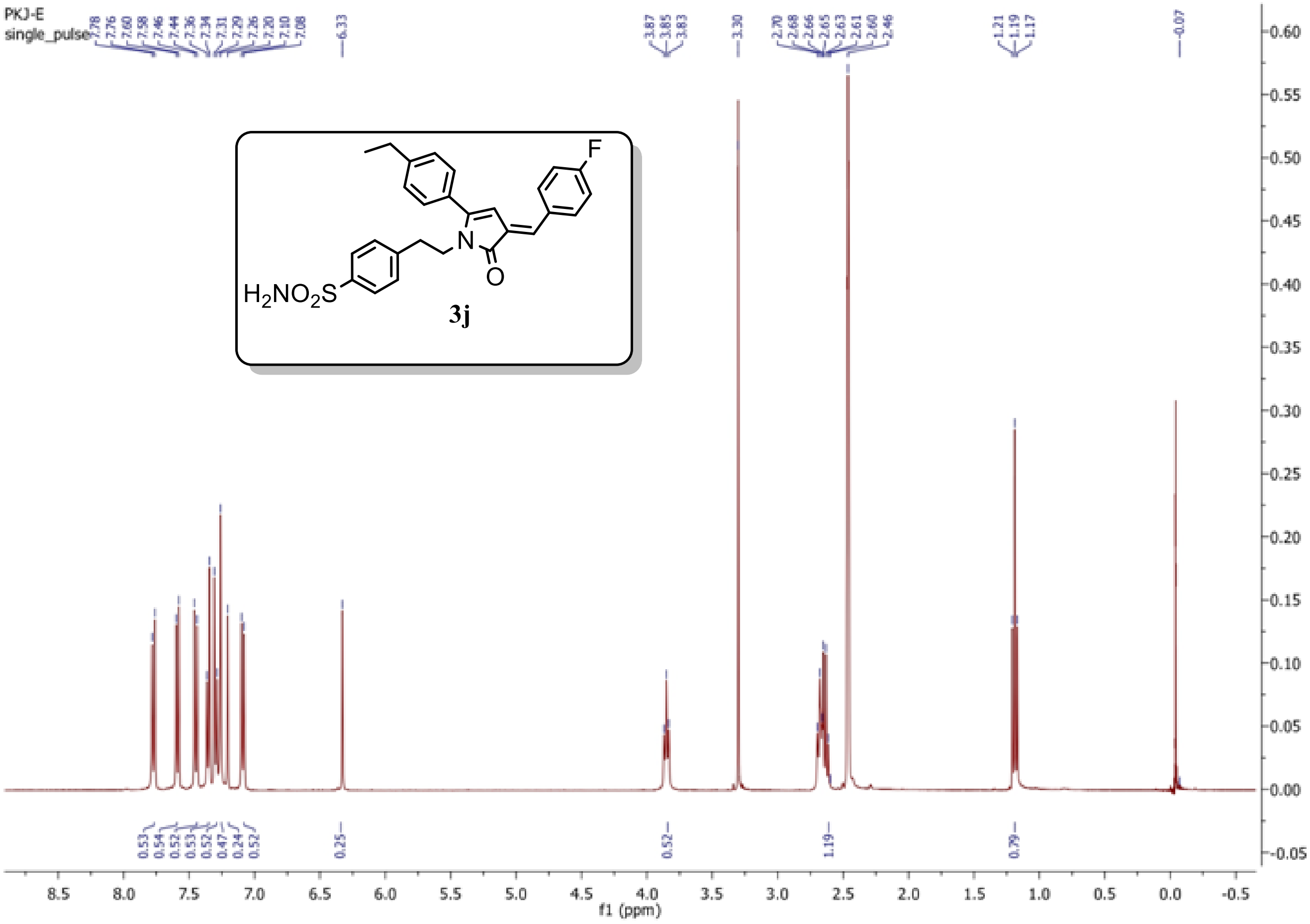
PKJ-E
single_pulse
7.78
7.76
7.60
7.58
7.46
7.44
7.36
7.34
7.31
7.29
7.26
7.20
7.10
7.08
6.33
3.87
3.85
3.83
3.30
2.70
2.68
2.66
2.65
2.63
2.61
2.60
2.46
1.21
1.19
1.17
-0.07
F
N
O
H_2NO_2S
3j
0.53
0.54
0.52
0.53
0.52
0.47
0.24
0.52
0.25
0.52
1.19
0.79
f1 (ppm)

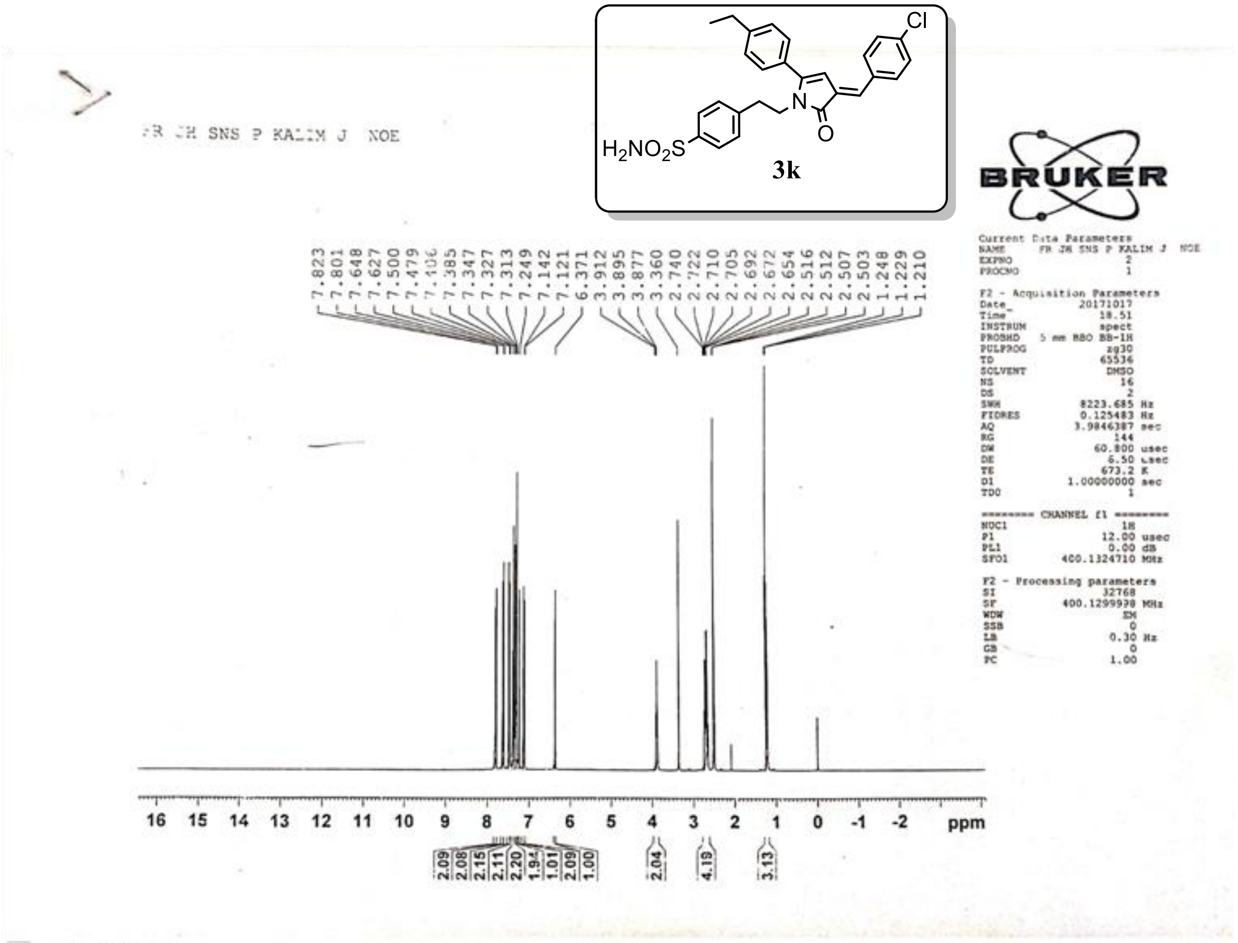

CS Scanned with CamScanner

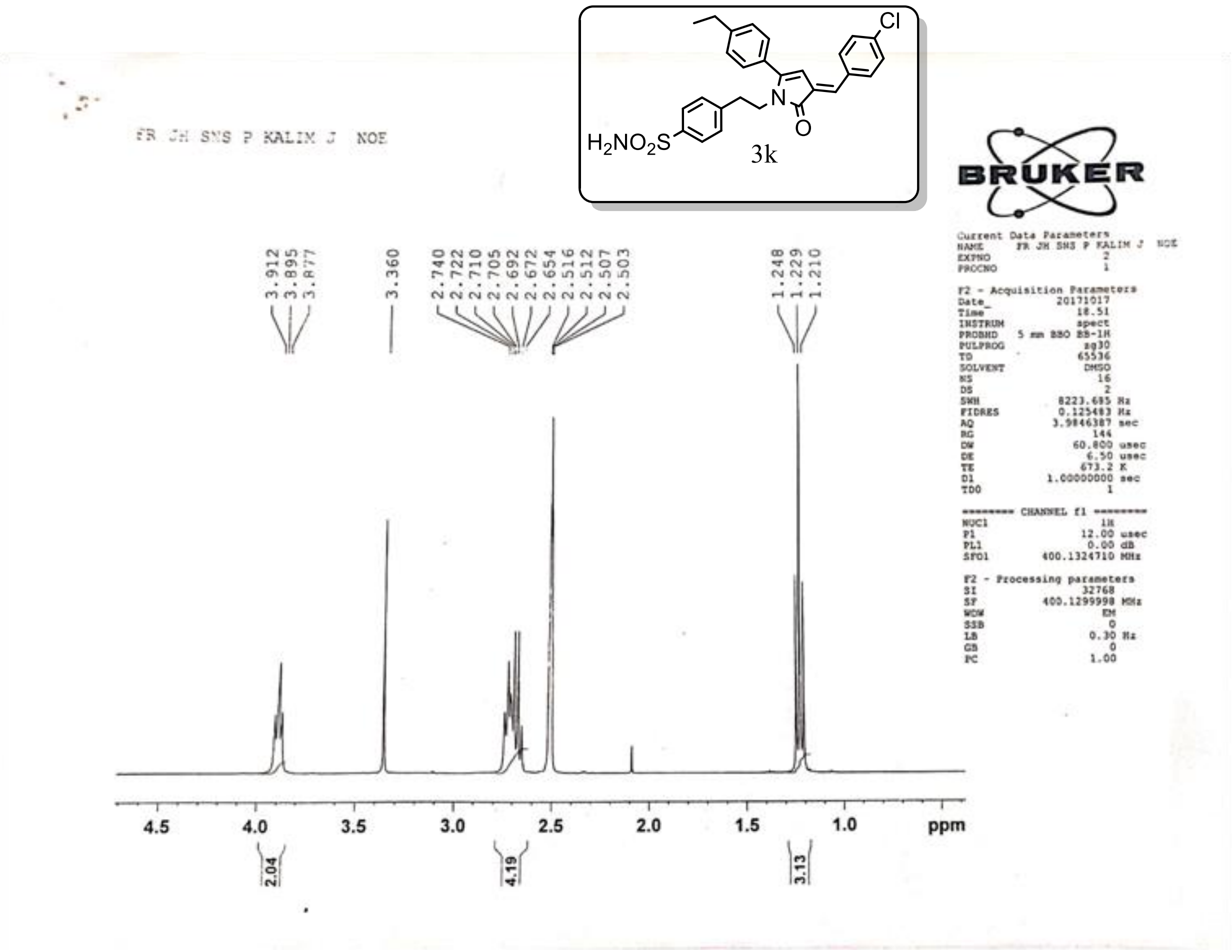

CS Scanned with CamScanner

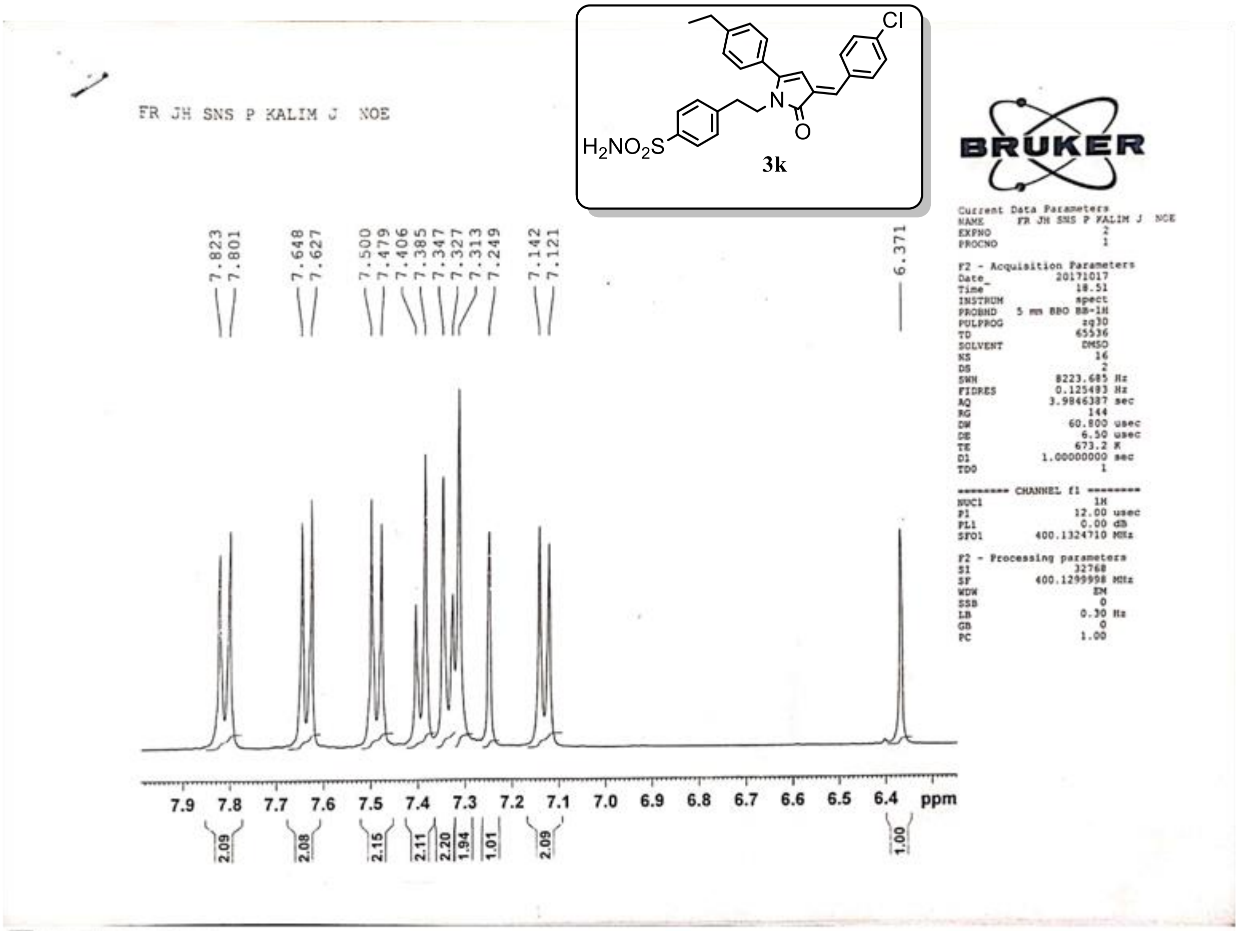

CS Scanned with CamScanner

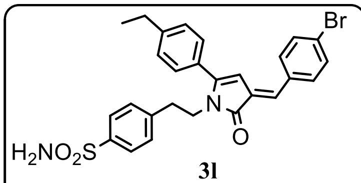
Br
N
O
H2NO2S

3l

p5

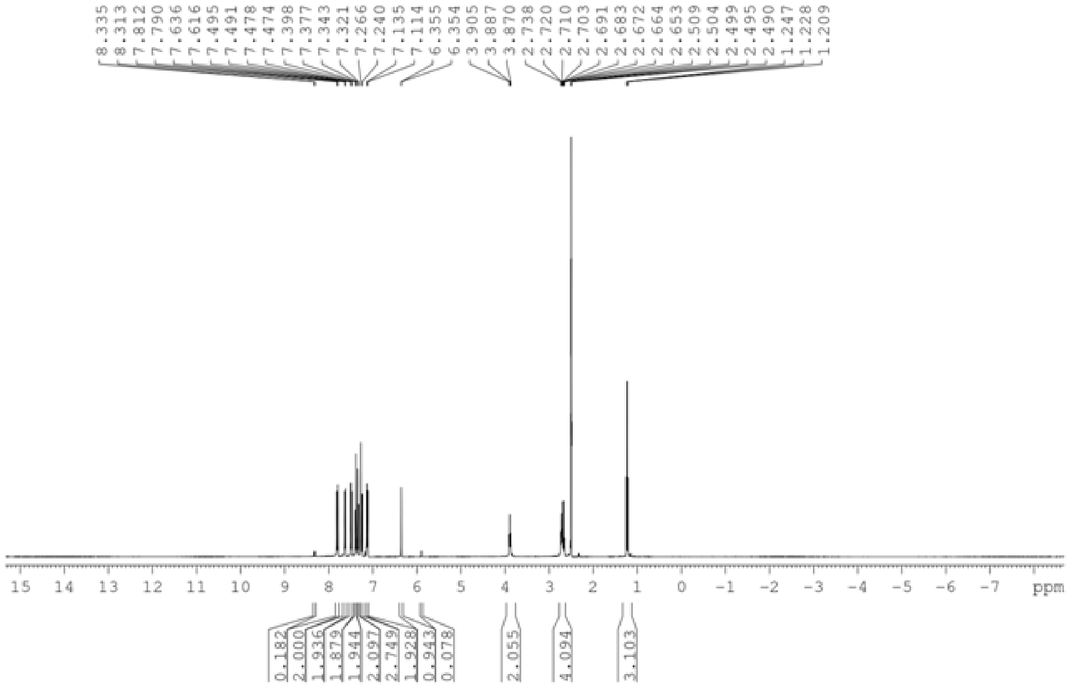
8.335
8.313
7.812
7.790
7.636
7.616
7.495
7.491
7.478
7.474
7.398
7.377
7.343
7.321
7.266
7.240
7.135
7.114
6.355
6.354
3.905
3.887
3.870
2.738
2.720
2.710
2.703
2.691
2.683
2.672
2.664
2.653
2.509
2.504
2.499
2.495
2.490
1.247
1.228
1.209
15 14 13 12 11 10 9 8 7 6 5 4 3 2 1 0 -1 -2 -3 -4 -5 -6 -7 ppm
0.182
2.000
1.936
1.879
1.944
2.097
2.749
1.928
0.943
0.078
2.055
4.094
3.103

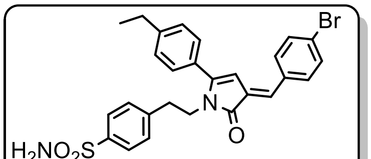
Br
N
O
H2NO2S

3l

p5

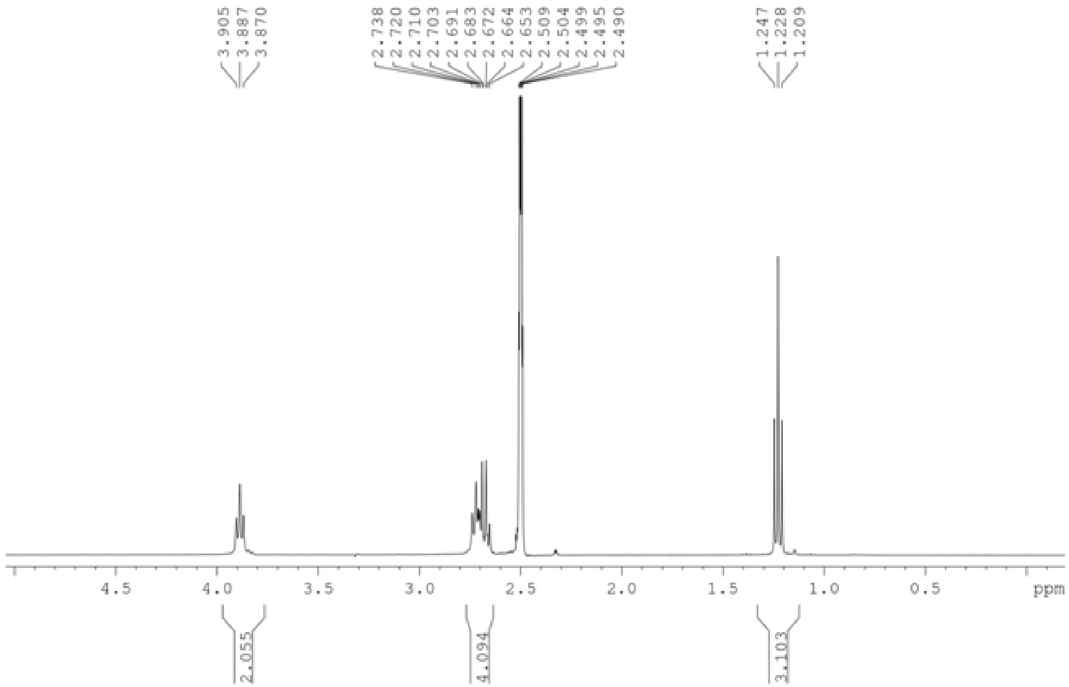
3.905
3.887
3.870
2.738
2.720
2.710
2.703
2.691
2.683
2.672
2.664
2.653
2.509
2.504
2.499
2.495
2.490
1.247
1.228
1.209
4.5
4.0
3.5
3.0
2.5
2.0
1.5
1.0
0.5
ppm
2.055
4.094
3.103

p5

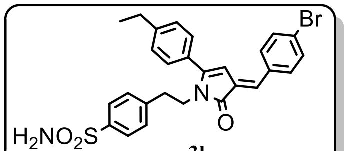

Br
N
O
H_2NO_2S

3l

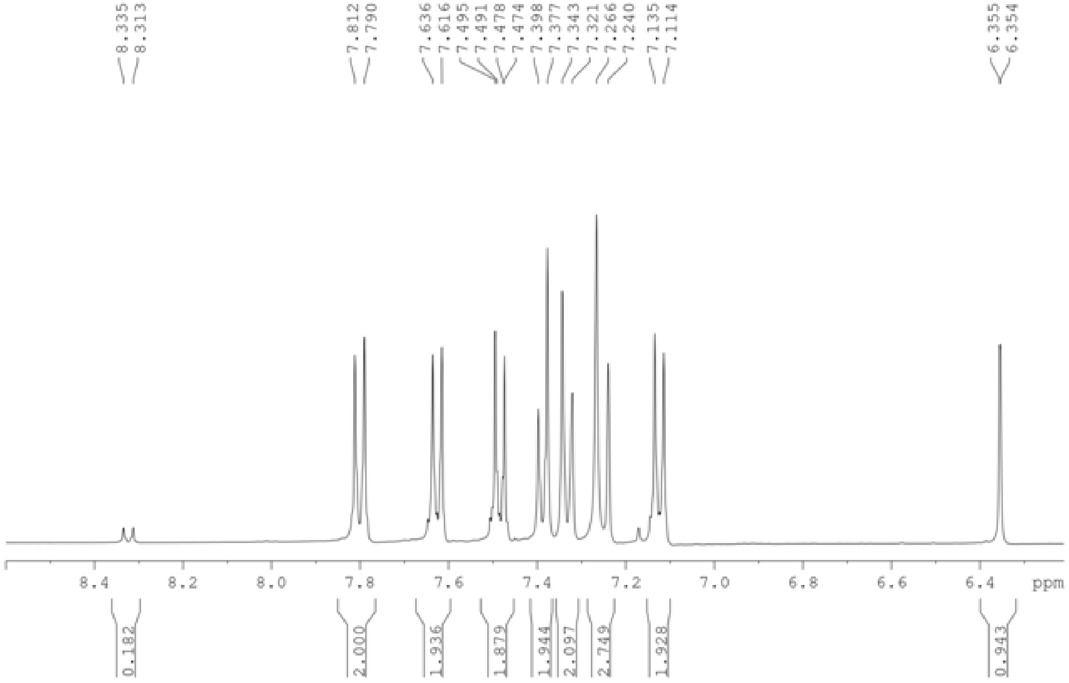

8.335
8.313
7.812
7.790
7.636
7.616
7.495
7.491
7.478
7.474
7.398
7.377
7.343
7.321
7.266
7.240
7.135
7.114
6.355
6.354
8.4
8.2
8.0
7.8
7.6
7.4
7.2
7.0
6.8
6.6
6.4
ppm
0.182
2.000
1.936
1.879
1.944
2.097
2.749
1.928
0.943

SNS FARHAT R PKJ Cl BD

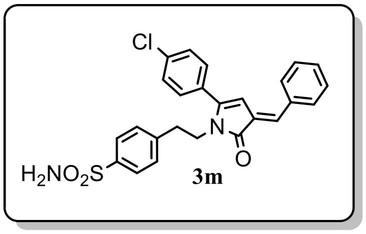

3m

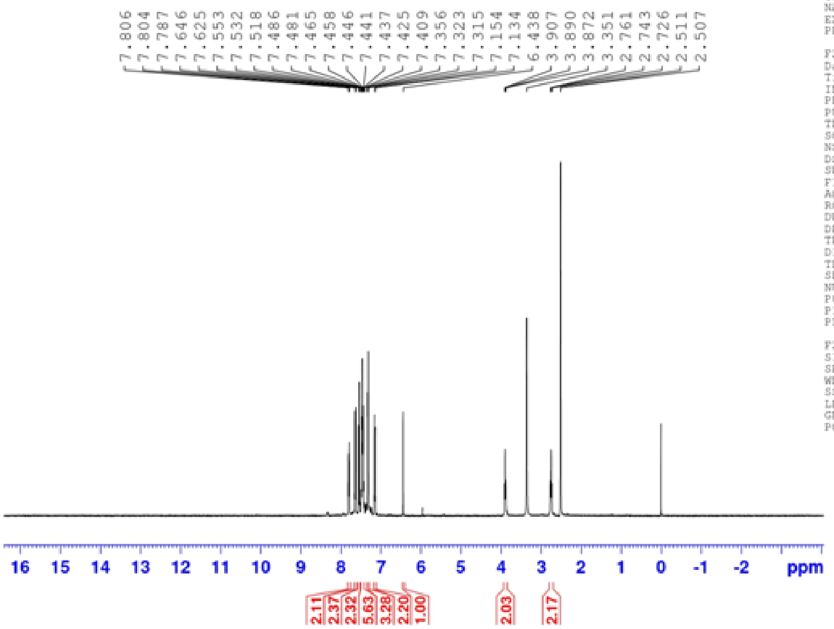


```
Current Data Parameters
NAME       SNS FARHAT R PKJ Cl BD
EXPNO                    5
PROCNO                   1

F2 - Acquisition Parameters
Date_             20220707
Time                 16.09 h
INSTRUM             Avance
PROBHD     Z8247_0054 (PH
PULPROG               zg30
TD                   65536
SOLVENT               DMSO
NS                      16
DS                       2
SWH               8196.722 Hz
FIDRES            0.250144 Hz
AQ               3.9976959 sec
RG                     101
DW                  61.000 usec
DE                   14.06 usec
TE                   298.2 K
D1              1.00000000 sec
TD0                      1
SFO1           400.1324708 MHz
NUC1                    1H
P0                    2.33 usec
P1                    7.00 usec
PLW1           14.19099998 W

F2 - Processing parameters
SI                   65536
SF             400.1300000 MHz
WDW                     EM
SSB                      0
LB                    0.30 Hz
GB                       0
PC                    1.00
```

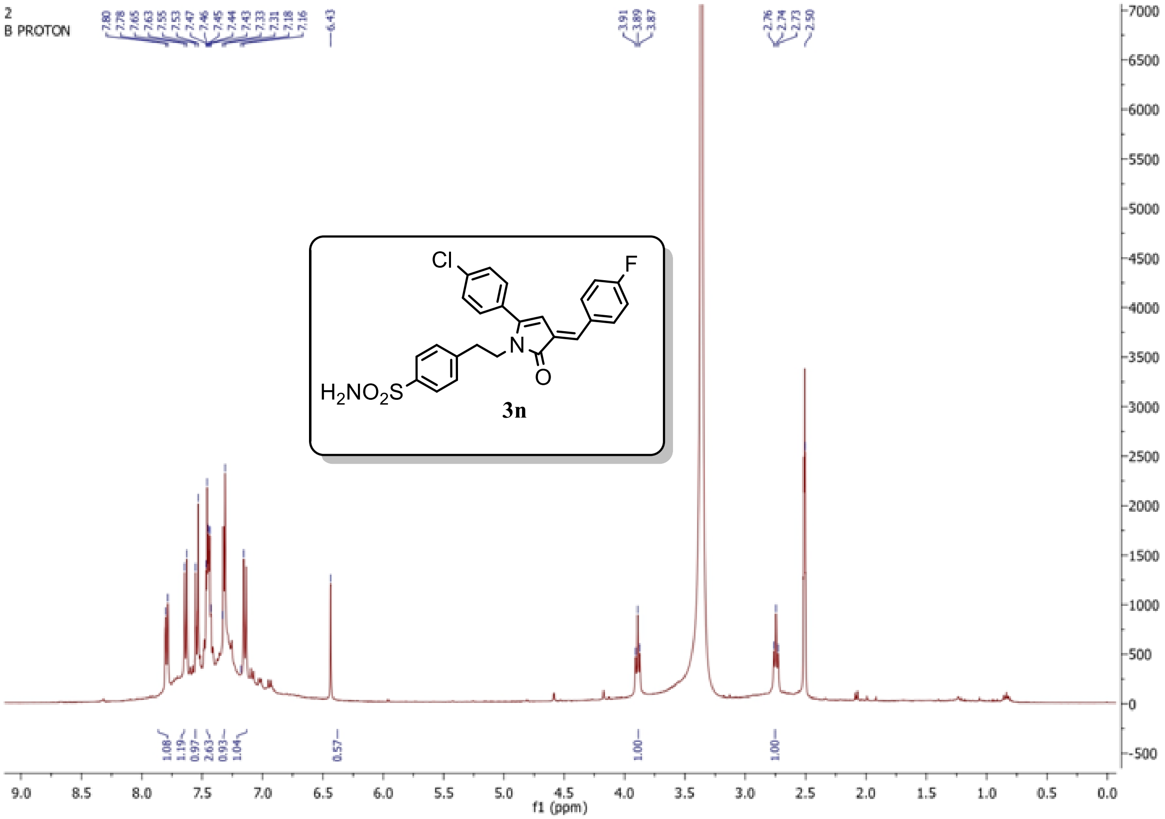
2
B PROTON
Cl
F
N
O
H_2NO_2S
3n
f1 (ppm)

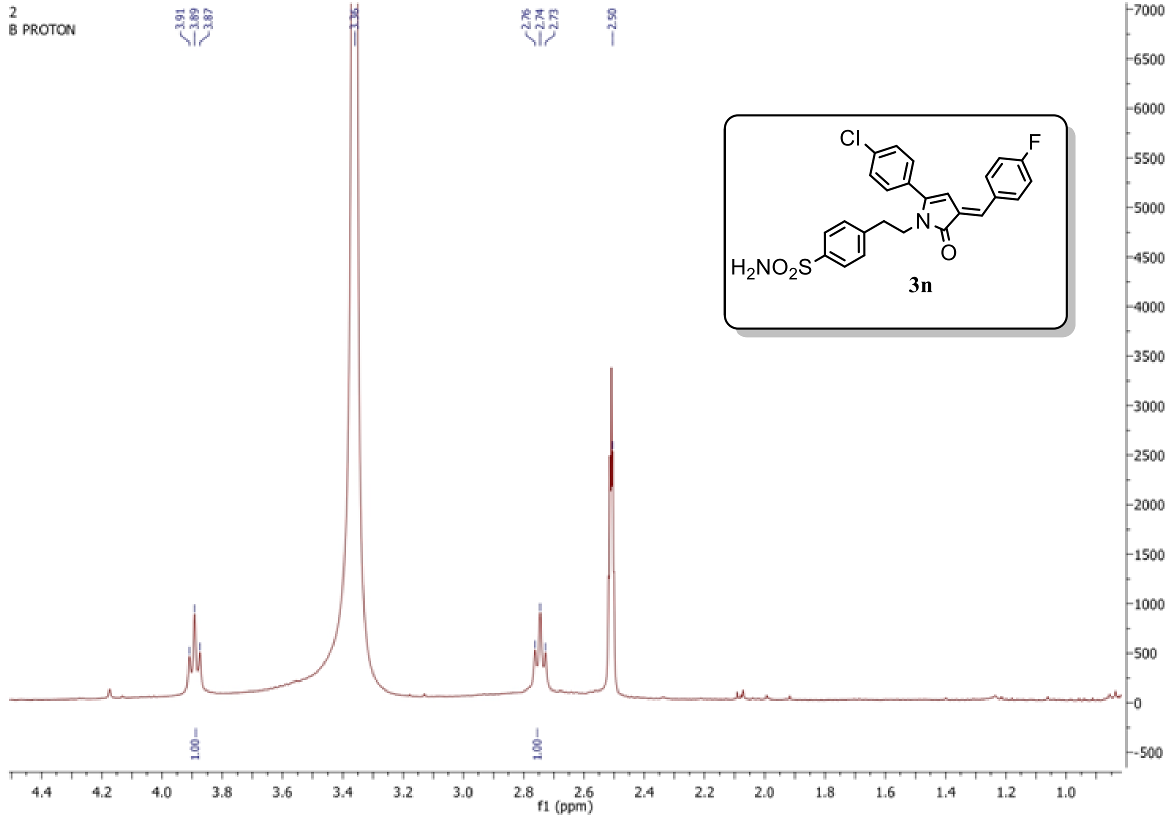
2
B PROTON
3.91
3.89
3.87
3.36
2.76
2.74
2.73
2.50
Cl
F
N
O
H_2NO_2S
3n
1.00
1.00
4.4 4.2 4.0 3.8 3.6 3.4 3.2 3.0 2.8 2.6 2.4 2.2 2.0 1.8 1.6 1.4 1.2 1.0
f1 (ppm)
7000 6500 6000 5500 5000 4500 4000 3500 3000 2500 2000 1500 1000 500 0 -500

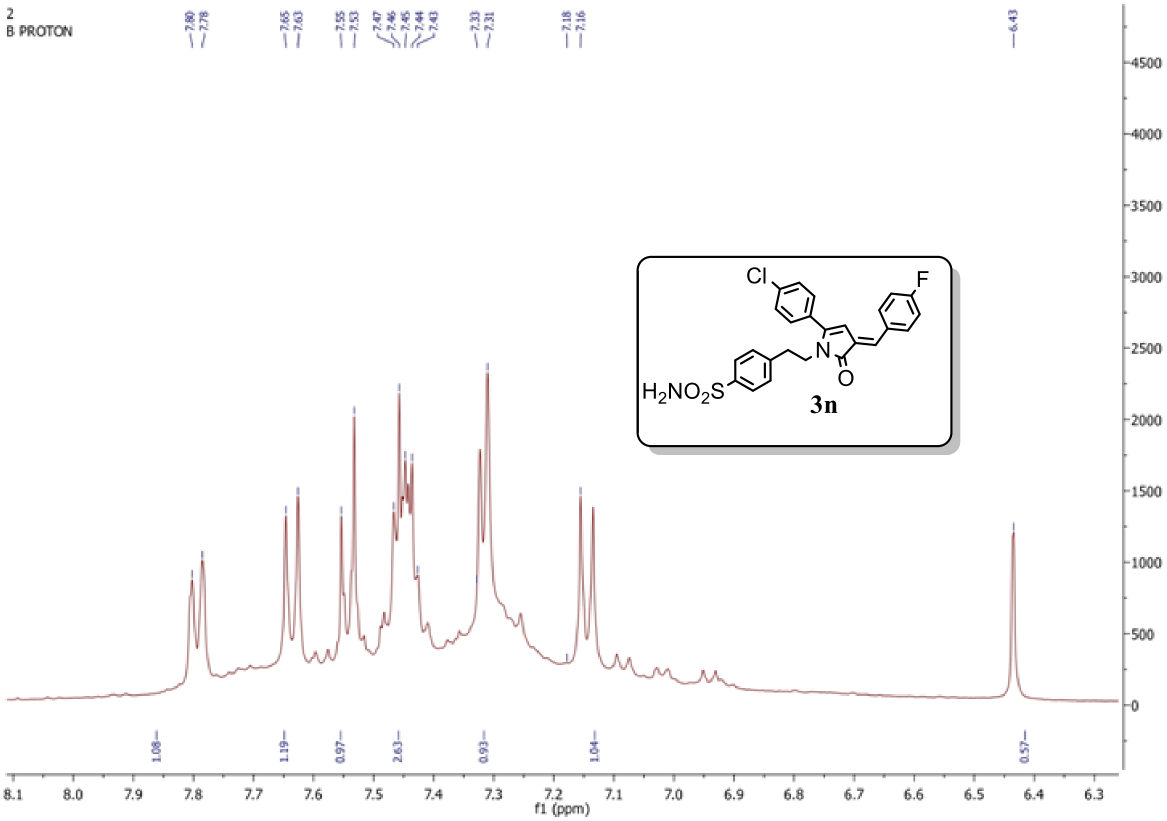

2
B PROTON
7.80
7.78
7.65
7.63
7.55
7.53
7.47
7.46
7.45
7.44
7.43
7.33
7.31
7.18
7.16
6.43
Cl
F
N
O
H_2NO_2S
3n
1.08
1.19
0.97
2.63
0.93
1.04
0.57
f1 (ppm)

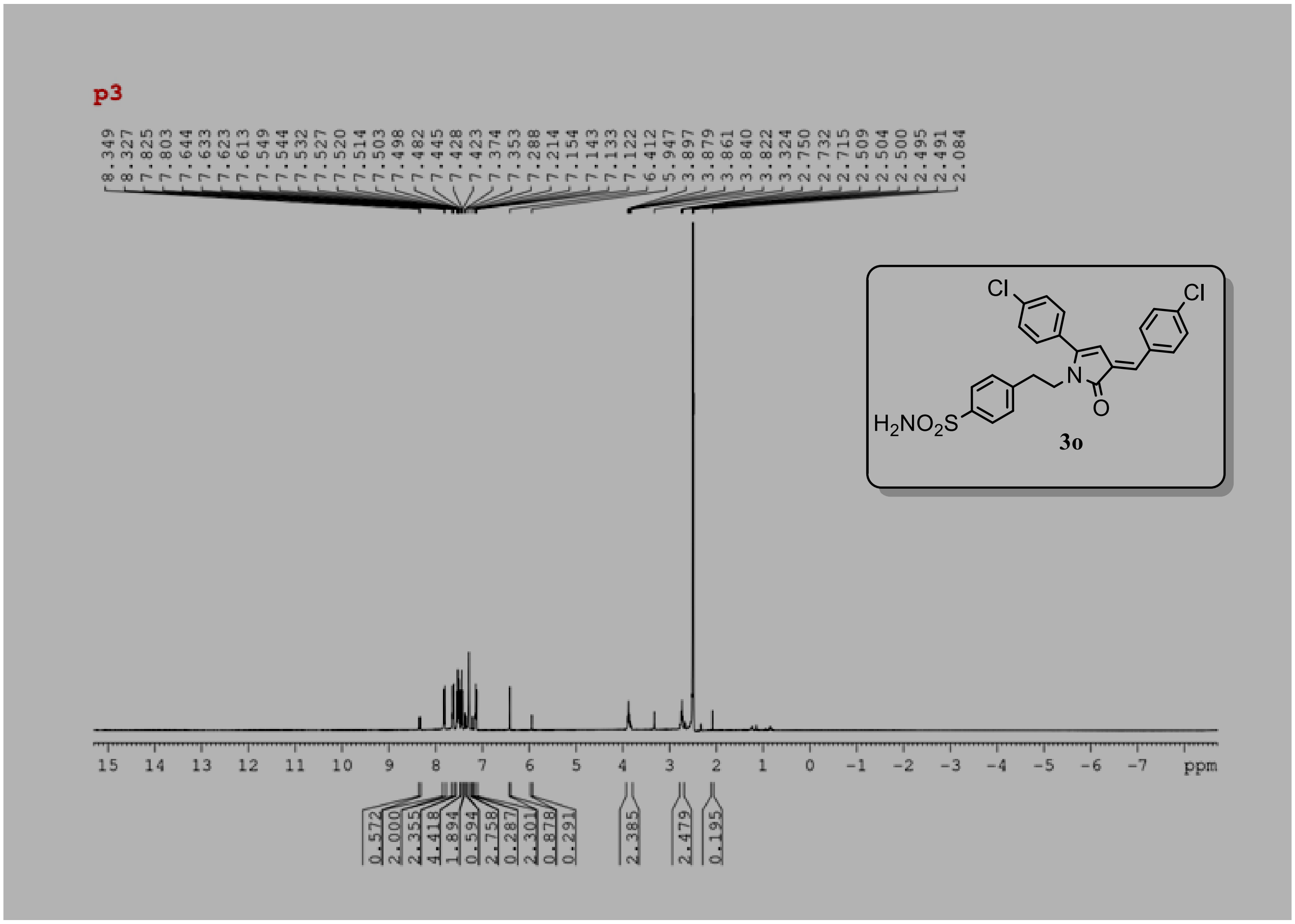
p3
8.349 8.327 7.825 7.803 7.644 7.633 7.623 7.613 7.549 7.544 7.532 7.527 7.520 7.514 7.503 7.498 7.482 7.445 7.428 7.423 7.374 7.353 7.288 7.214 7.154 7.143 7.133 7.122 6.412 5.947 3.897 3.879 3.861 3.840 3.822 3.324 2.750 2.732 2.715 2.509 2.504 2.500 2.495 2.491 2.084
Cl
Cl
N
O
H_2NO_2S
3o
15 14 13 12 11 10 9 8 7 6 5 4 3 2 1 0 -1 -2 -3 -4 -5 -6 -7 ppm
0.572 2.000 2.355 4.418 1.894 0.594 2.758 0.287 2.301 0.878 0.291 2.385 2.479 0.195

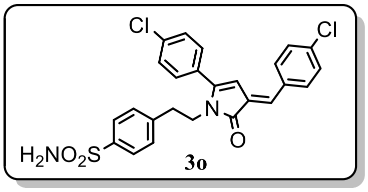
Cl
Cl
H_2NO_2S
N
O
3o

p3

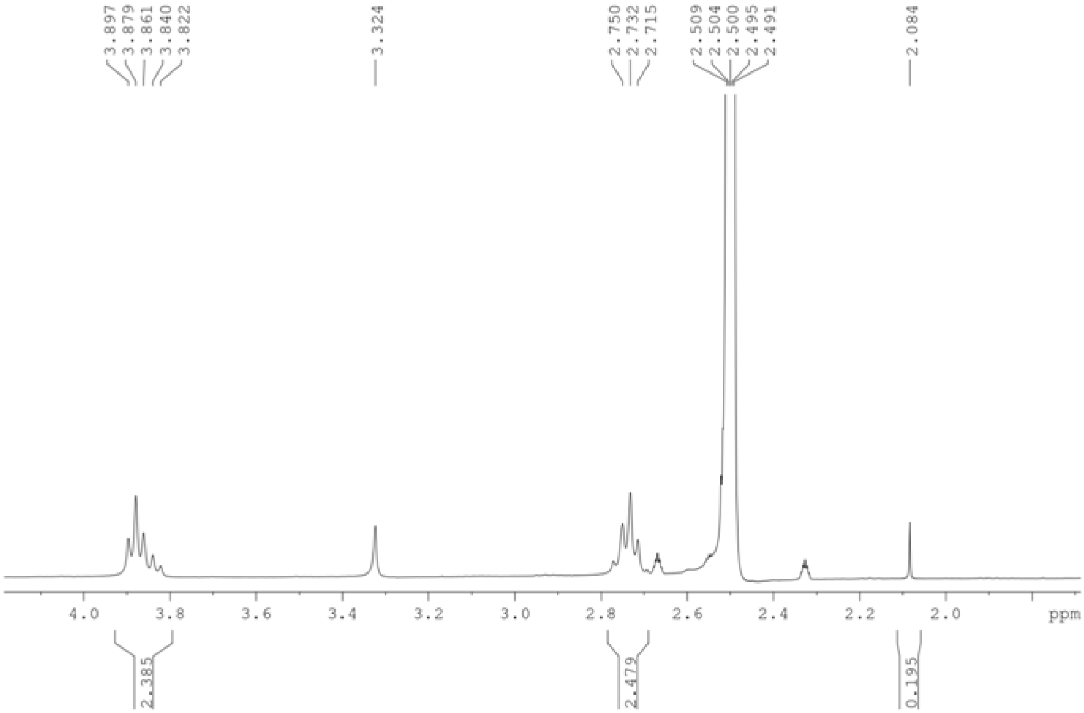
3.897
3.879
3.861
3.840
3.822
3.324
2.750
2.732
2.715
2.509
2.504
2.500
2.495
2.491
2.084
4.0
3.8
3.6
3.4
3.2
3.0
2.8
2.6
2.4
2.2
2.0
ppm
2.385
2.479
0.195

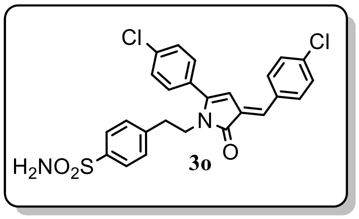
Cl
Cl
N
O
H_2NO_2S

3o

p3

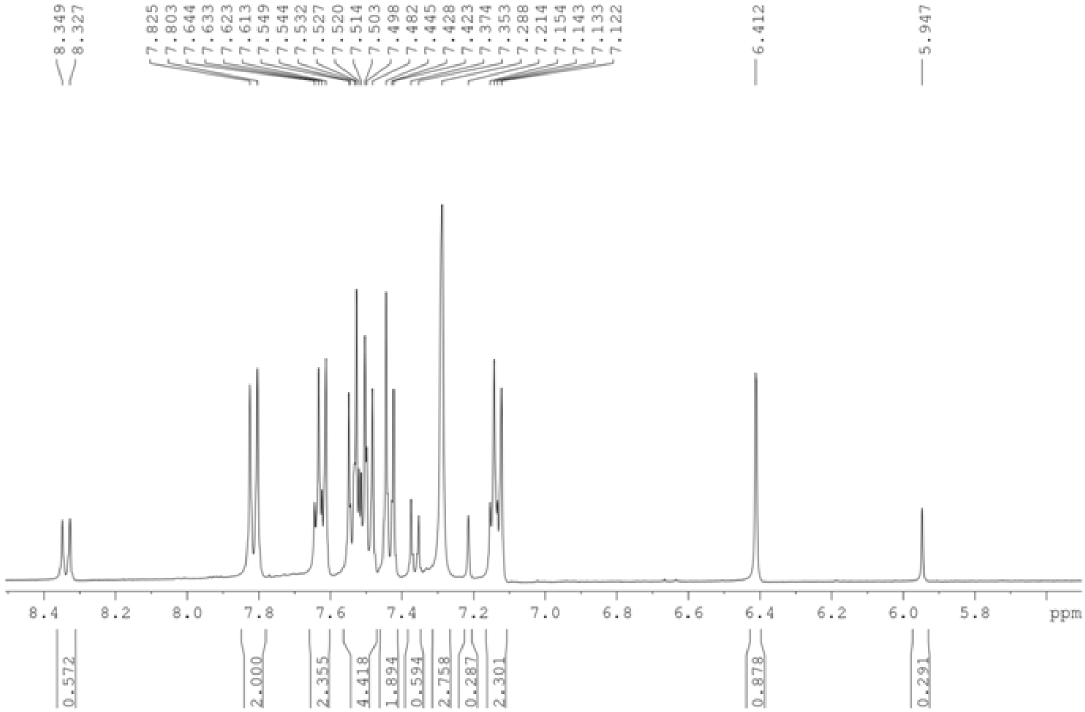
8.349
8.327
7.825
7.803
7.644
7.633
7.623
7.613
7.549
7.544
7.532
7.527
7.520
7.514
7.503
7.498
7.482
7.445
7.428
7.423
7.374
7.353
7.288
7.214
7.154
7.143
7.133
7.122
6.412
5.947
8.4
8.2
8.0
7.8
7.6
7.4
7.2
7.0
6.8
6.6
6.4
6.2
6.0
5.8
ppm
0.572
2.000
2.355
4.418
1.894
0.594
2.758
0.287
2.301
0.878
0.291

p4

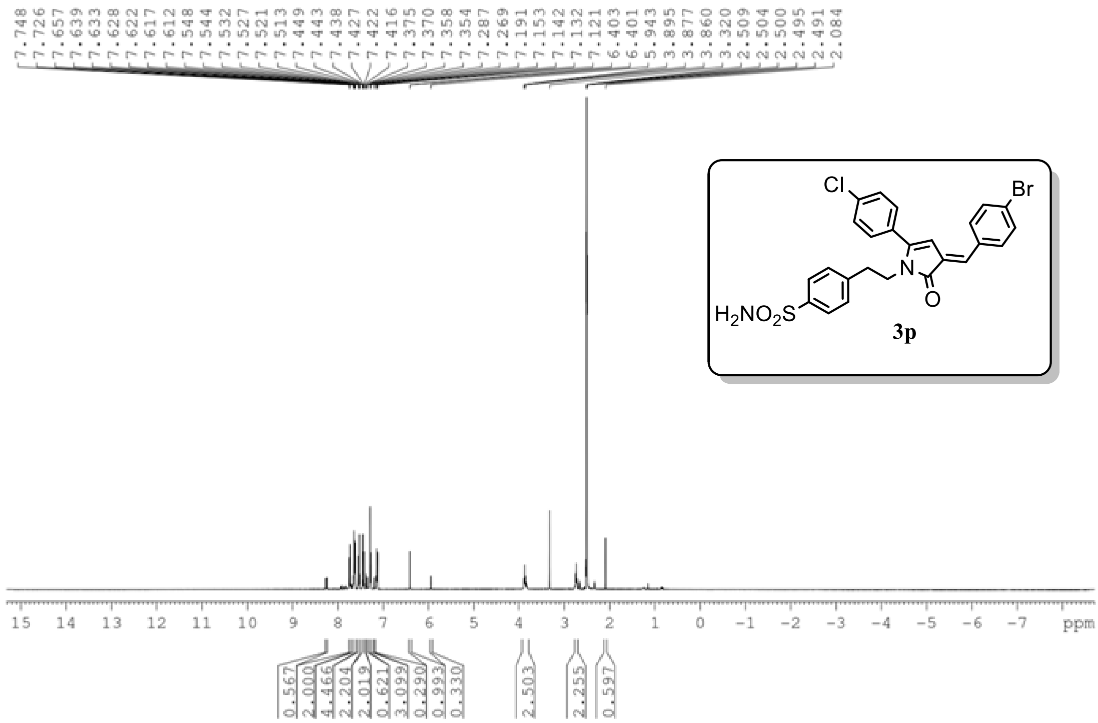
7.748
7.726
7.657
7.639
7.633
7.628
7.622
7.617
7.612
7.548
7.544
7.532
7.527
7.521
7.513
7.449
7.443
7.438
7.427
7.422
7.416
7.375
7.370
7.358
7.354
7.287
7.269
7.191
7.153
7.142
7.132
7.121
6.403
6.401
5.943
3.895
3.877
3.860
3.320
2.509
2.504
2.500
2.495
2.491
2.084
Cl
Br
N
O
H2NO2S
3p
15 14 13 12 11 10 9 8 7 6 5 4 3 2 1 0 -1 -2 -3 -4 -5 -6 -7 ppm
0.567
2.000
4.466
2.204
2.019
0.621
3.099
0.290
0.993
0.330
2.503
2.255
0.597

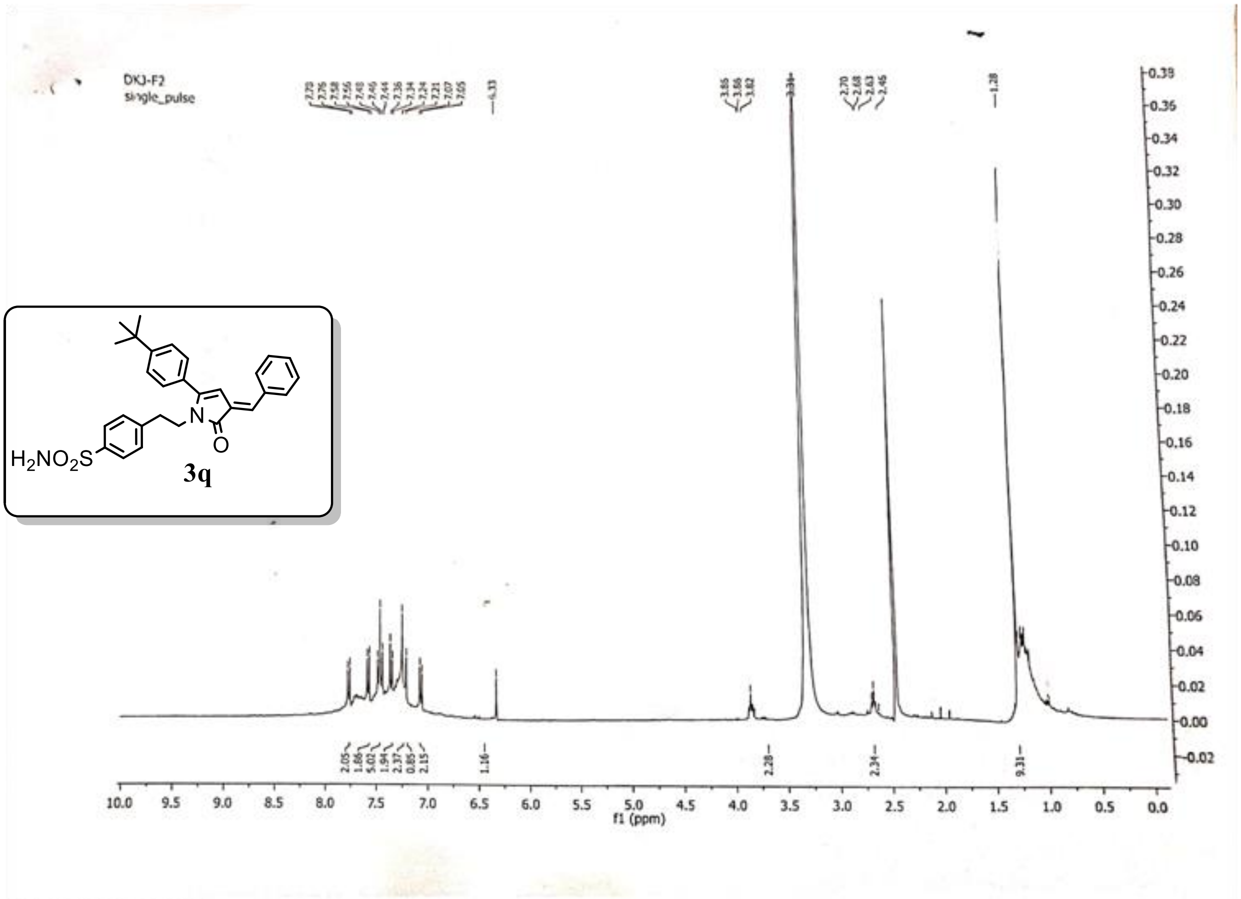

CS Scanned with CamScanner

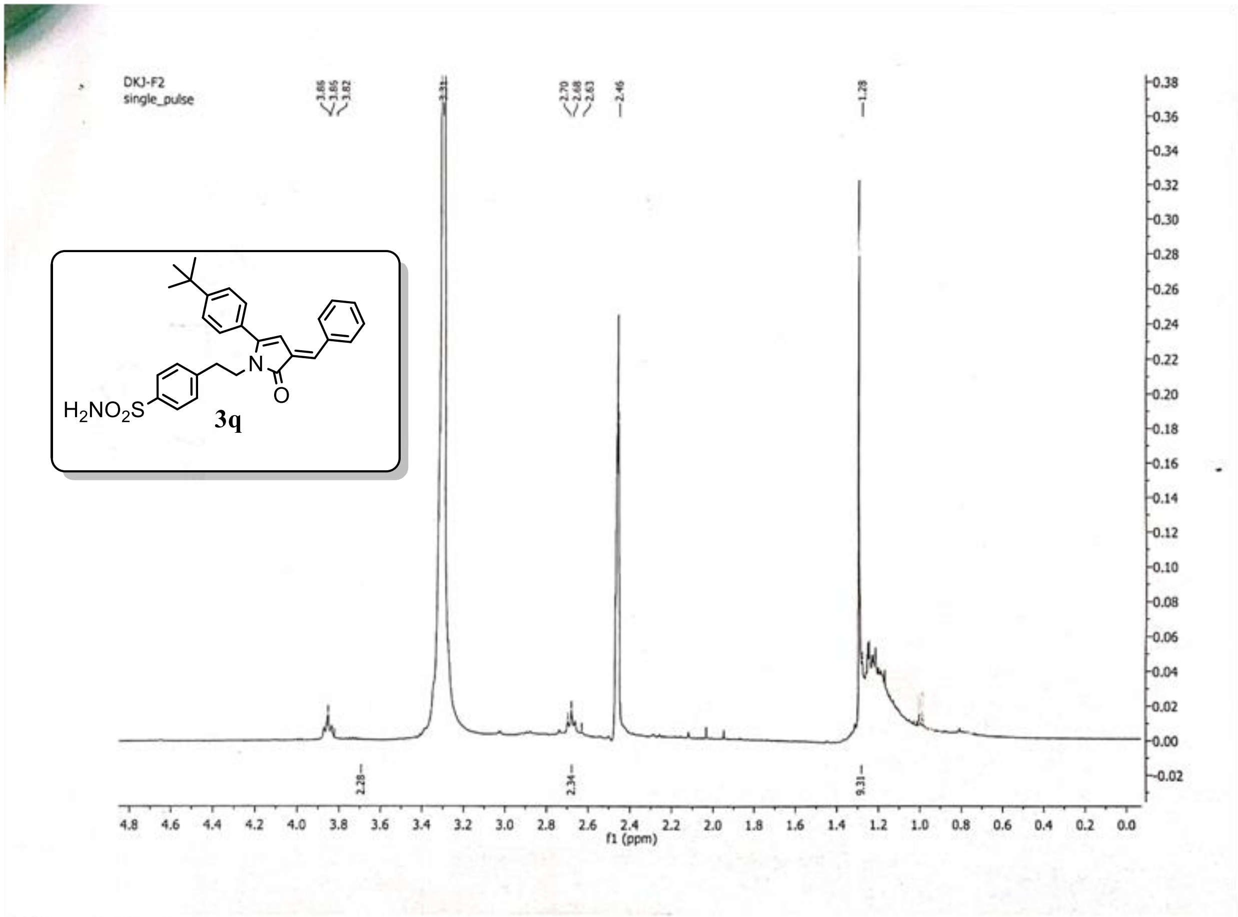

Scanned with CamScanner

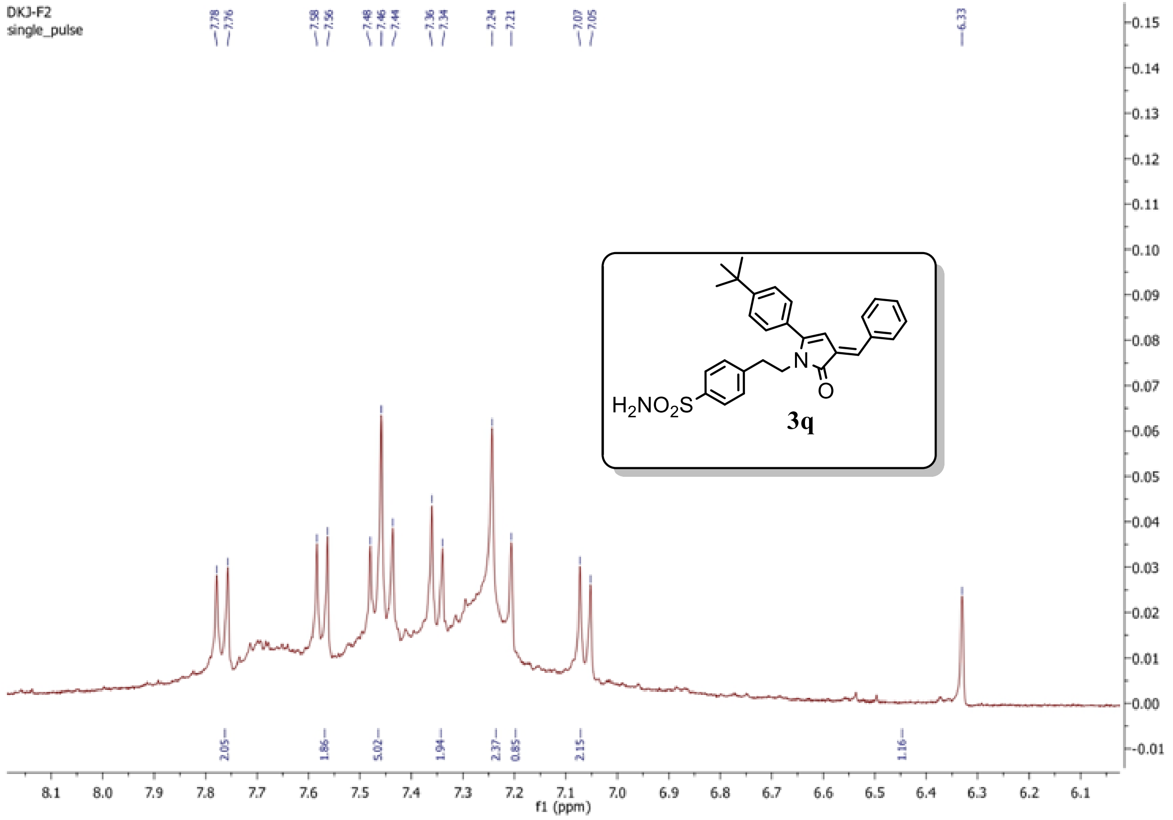
DKJ-F2
single_pulse
7.78
7.76
7.58
7.56
7.48
7.46
7.44
7.36
7.34
7.24
7.21
7.07
7.05
6.33
2.05
1.86
5.02
1.94
2.37
0.85
2.15
1.16
H2NO2S
N
O
3q
f1 (ppm)

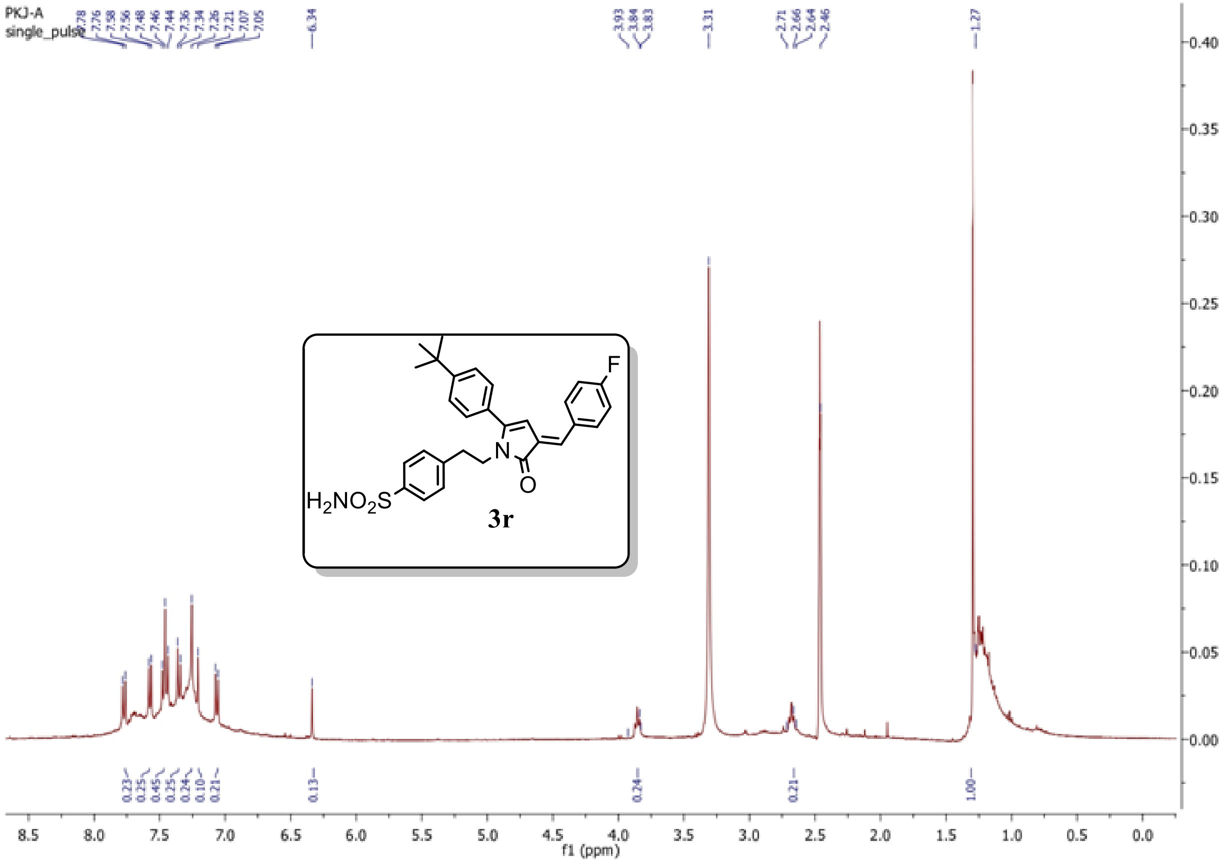
PKJ-A
single_pulse
3r
H2NO2S
f1 (ppm)

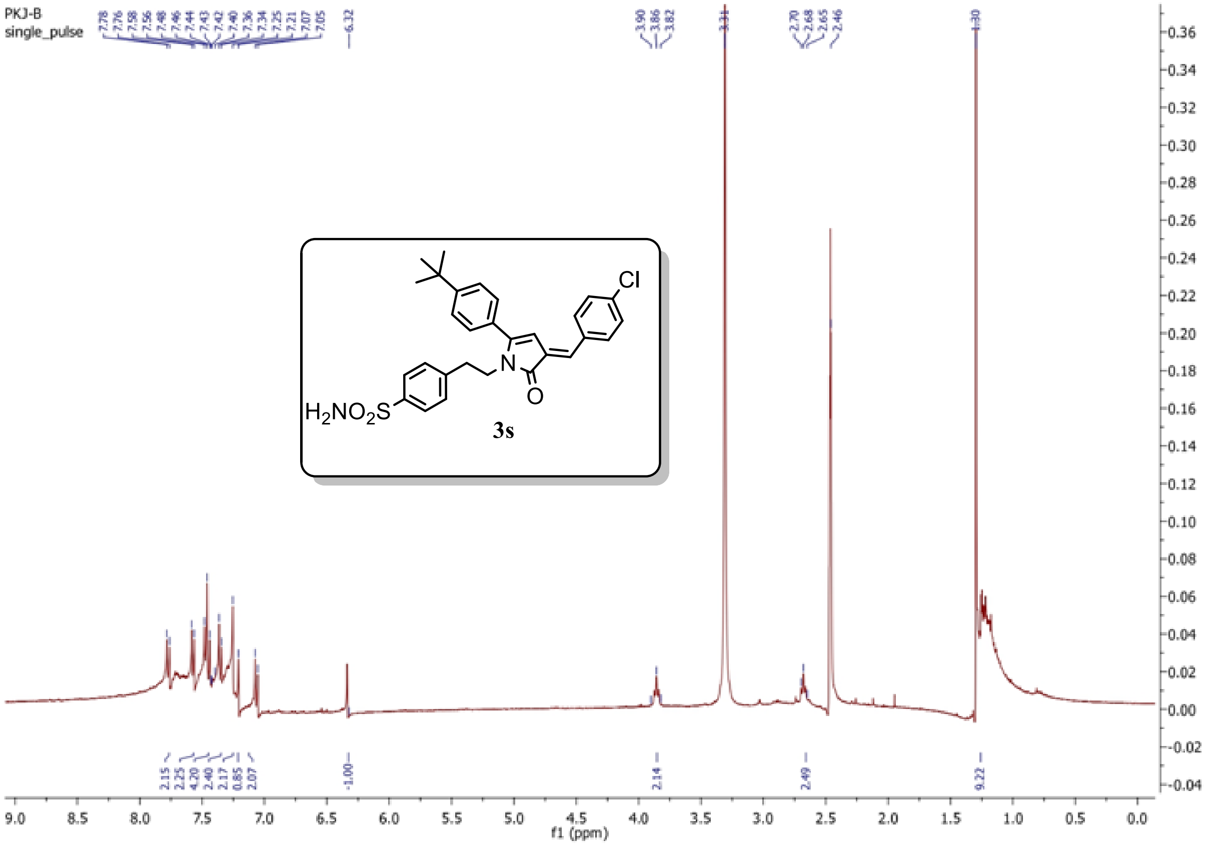
PKJ-B
single_pulse
3s
f1 (ppm)

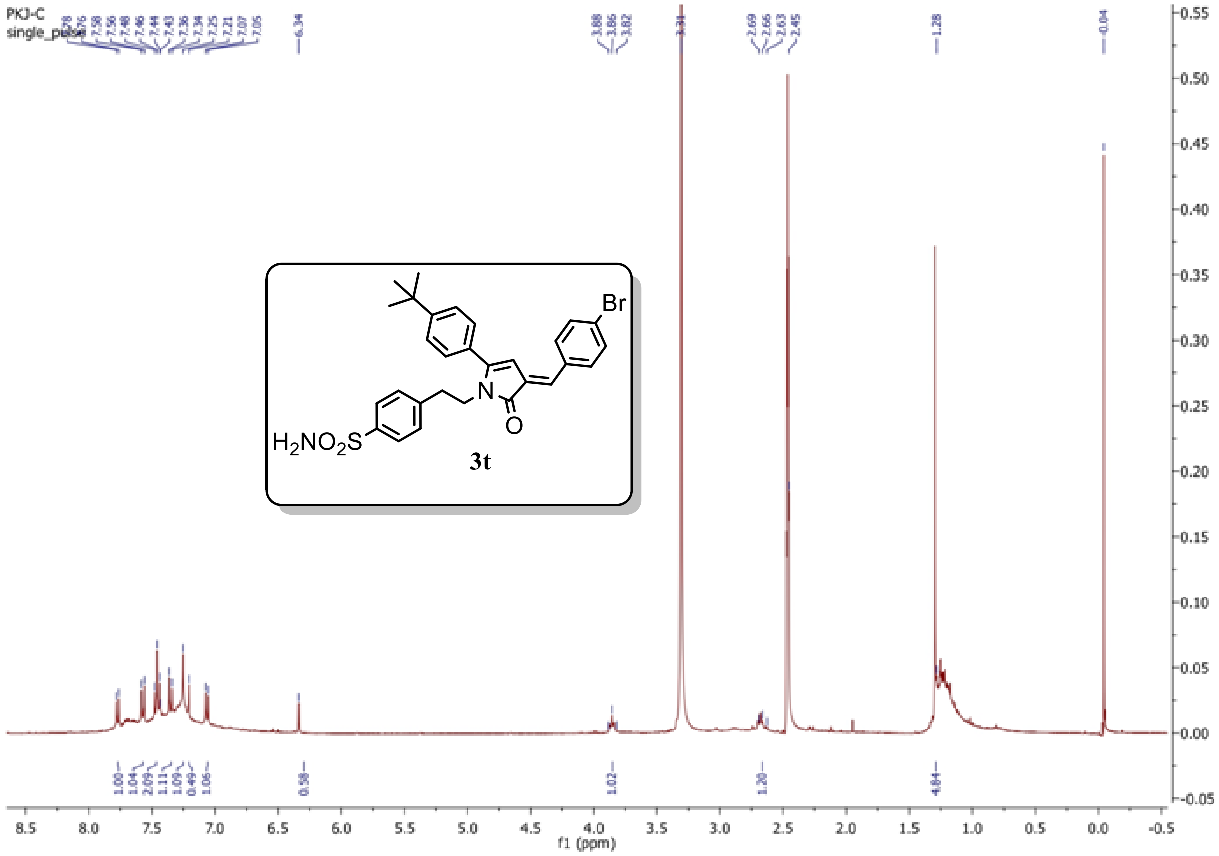

2D NMR OF 3K (NOE)

Fig S1

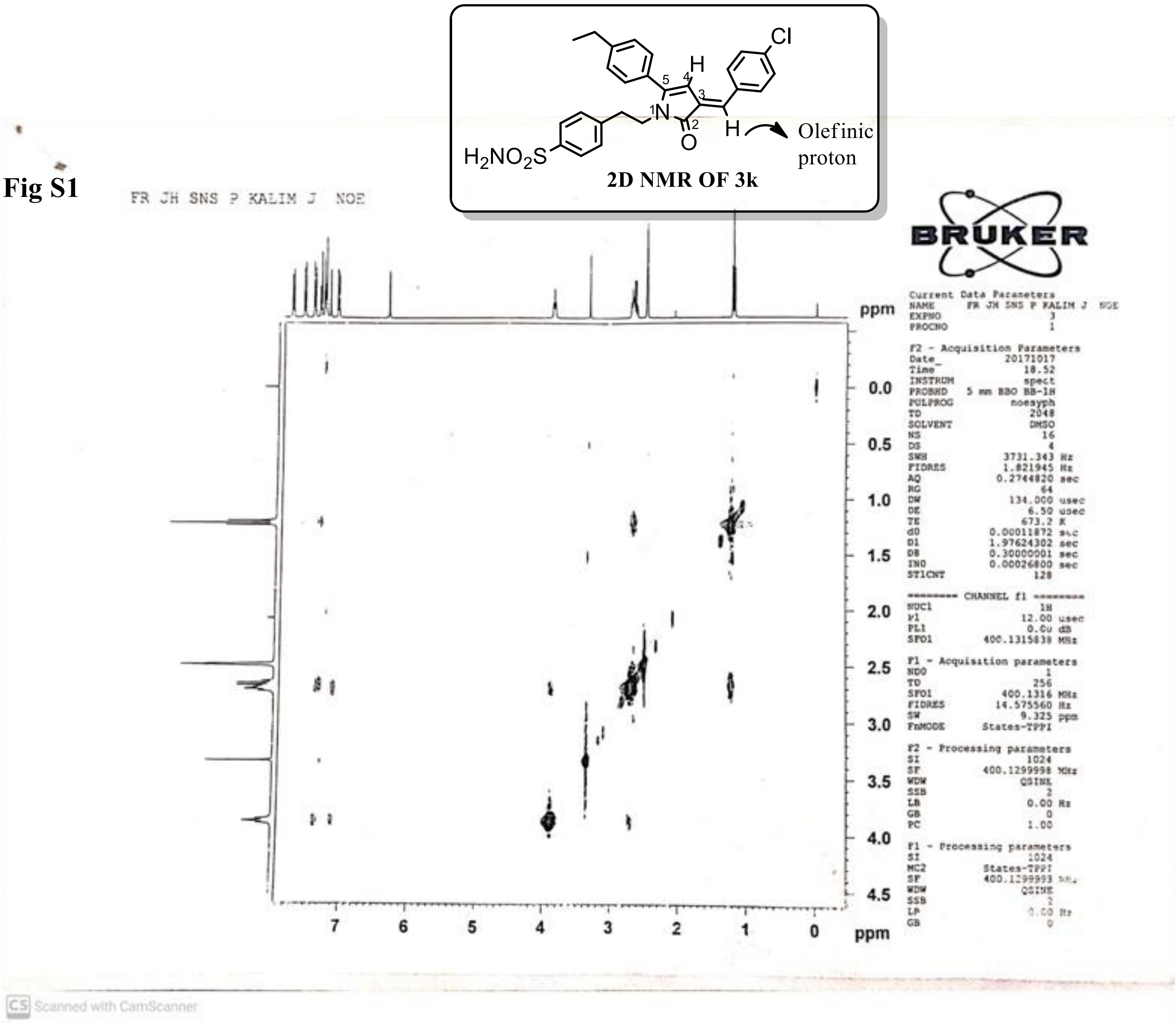

2D NMR OF 3k
Olefinic proton
H_2NO_2S
Cl
FR JH SNS P KALIM J NOE
BRUKER
ppm
Scanned with CamScanner

Fig S2

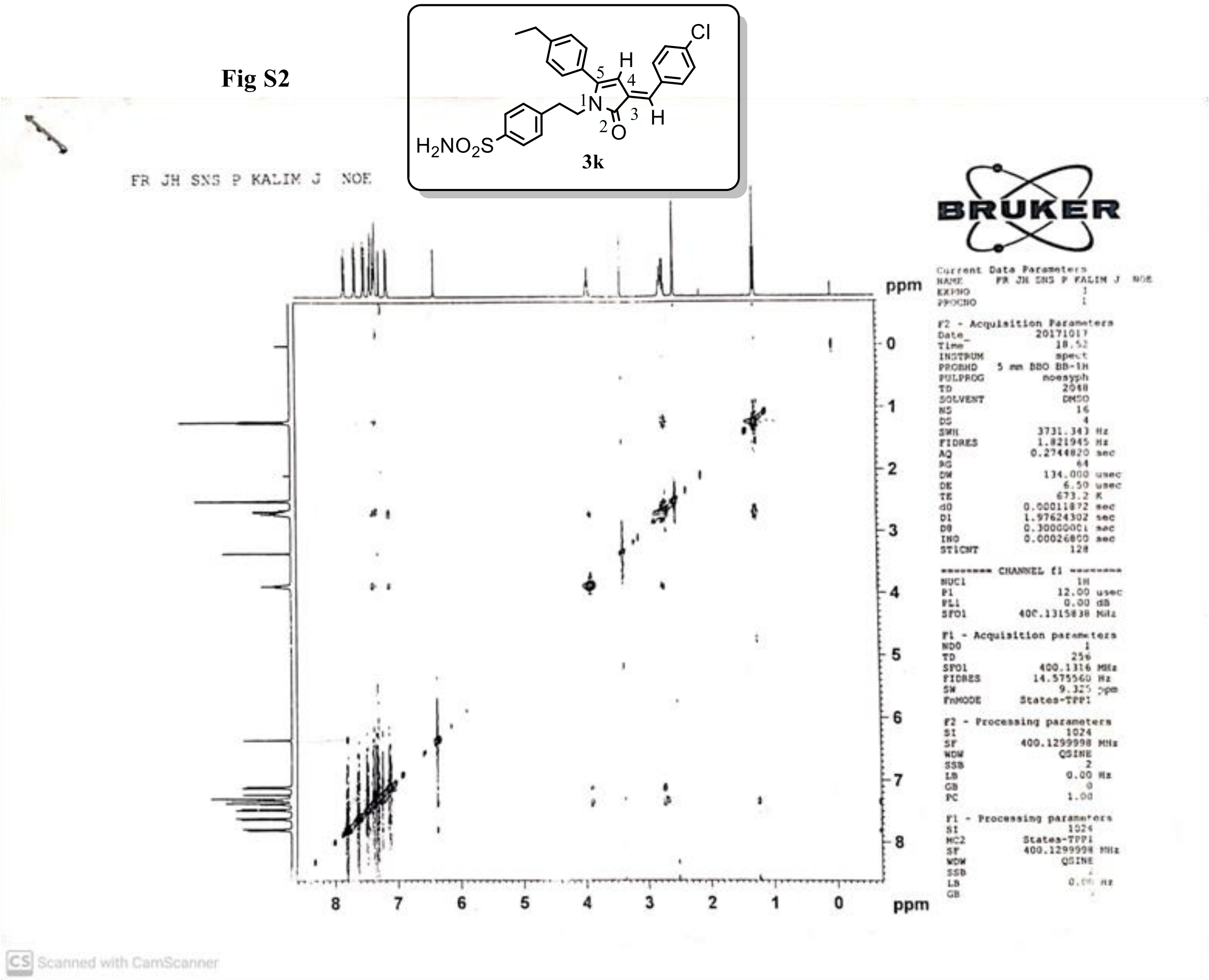

C13 NMR Spectra

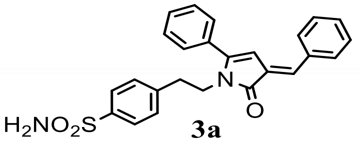
H2NO2S
N
O

3a

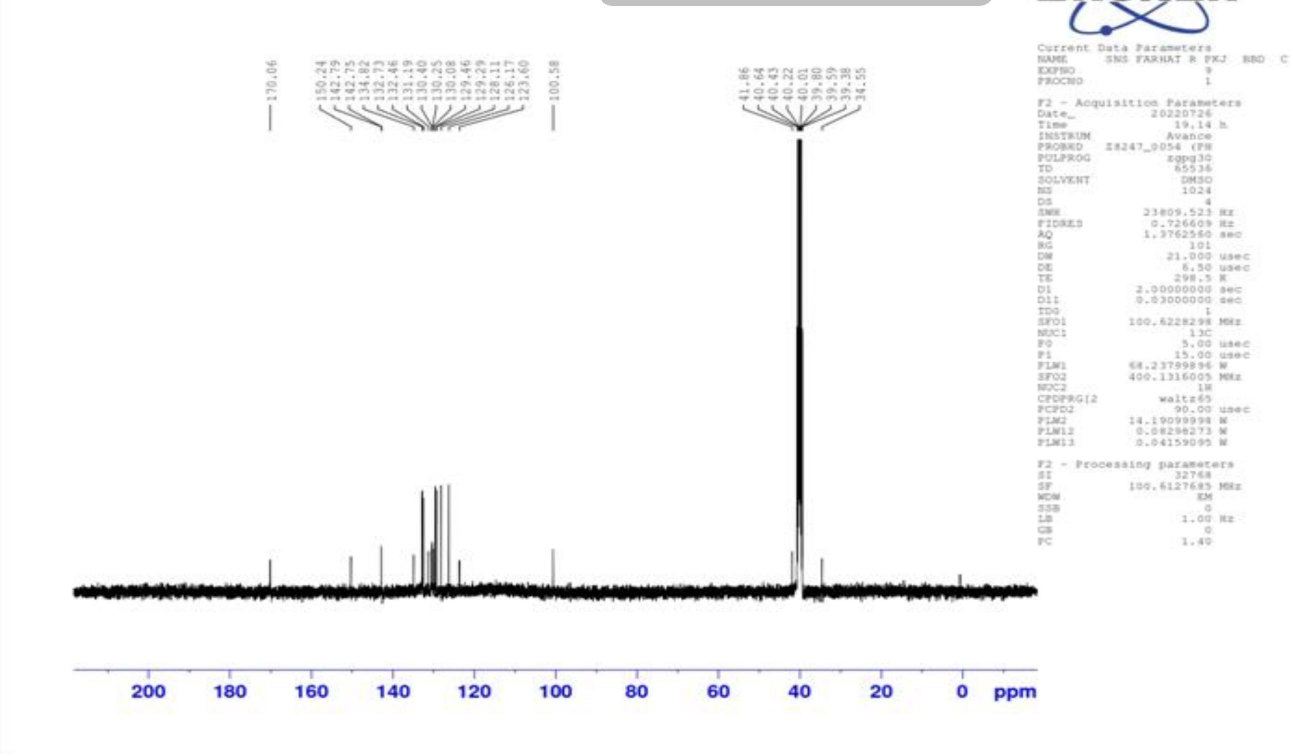
SNS FARHAT R PKJ BBD
170.06
150.24
142.79
142.75
134.82
132.73
132.46
131.19
130.40
130.25
130.08
129.46
129.29
128.11
126.17
123.60
100.58
41.86
40.64
40.43
40.22
40.01
39.80
39.59
39.38
34.55
BRUKER
Current Data Parameters
NAME SNS FARHAT R PKJ BBD C
EXPNO 9
PROCNO 1
F2 - Acquisition Parameters
Date_ 20220726
Time 19.14 h
INSTRUM Avance
PROBHD Z8247_0054 (PH
PULPROG zgpg30
TD 65536
SOLVENT DMSO
NS 1024
DS 4
SWH 23809.523 Hz
FIDRES 0.726609 Hz
AQ 1.3762560 sec
RG 101
DW 21.000 usec
DE 6.50 usec
TE 298.5 K
D1 2.00000000 sec
D11 0.03000000 sec
TD0 1
SFO1 100.6228298 MHz
NUC1 13C
P0 5.00 usec
P1 15.00 usec
PLW1 68.23799896 W
SFO2 400.1316005 MHz
NUC2 1H
CPDPRG[2 waltz65
PCPD2 90.00 usec
PLW2 14.19099998 W
PLW12 0.08298273 W
PLW13 0.04159095 W
F2 - Processing parameters
SI 32768
SF 100.6127685 MHz
WDW EM
SSB 0
LB 1.00 Hz
GB 0
PC 1.40
200
180
160
140
120
100
80
60
40
20
0
ppm

SNS FARHAT R PKJ BF

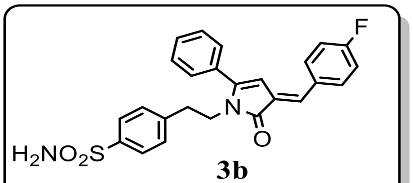

3b

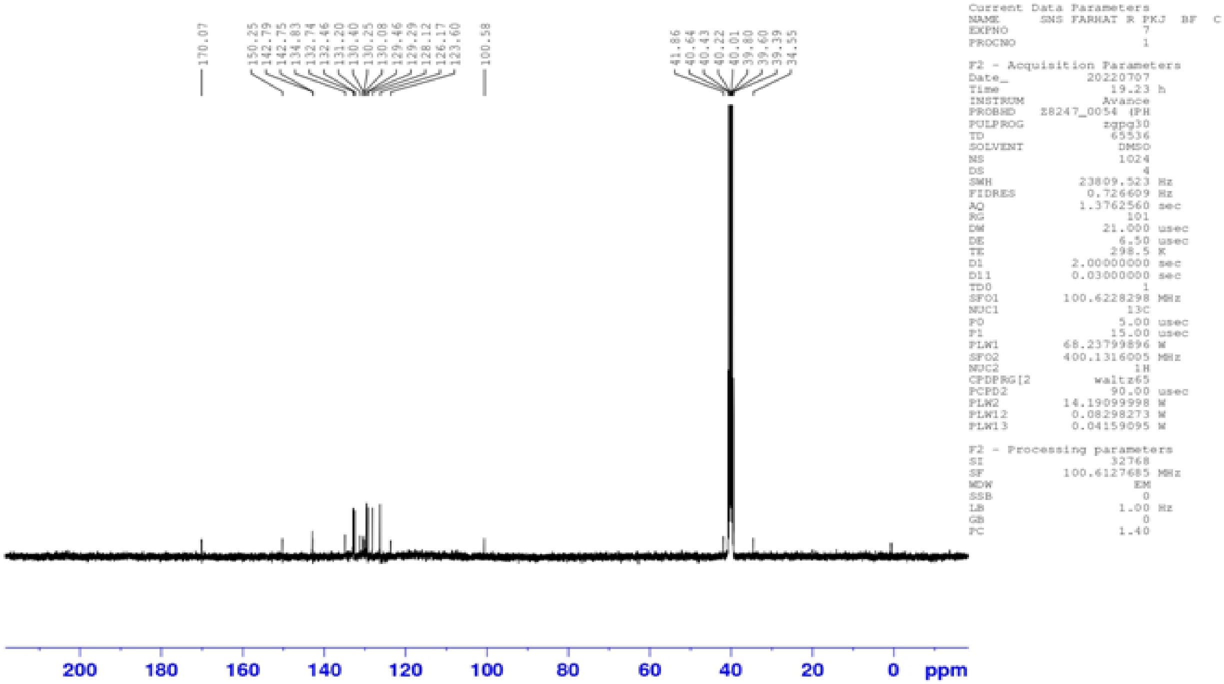

SNS FARHAT R PKJ BF

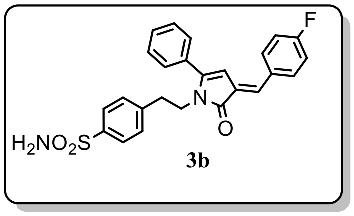

3b

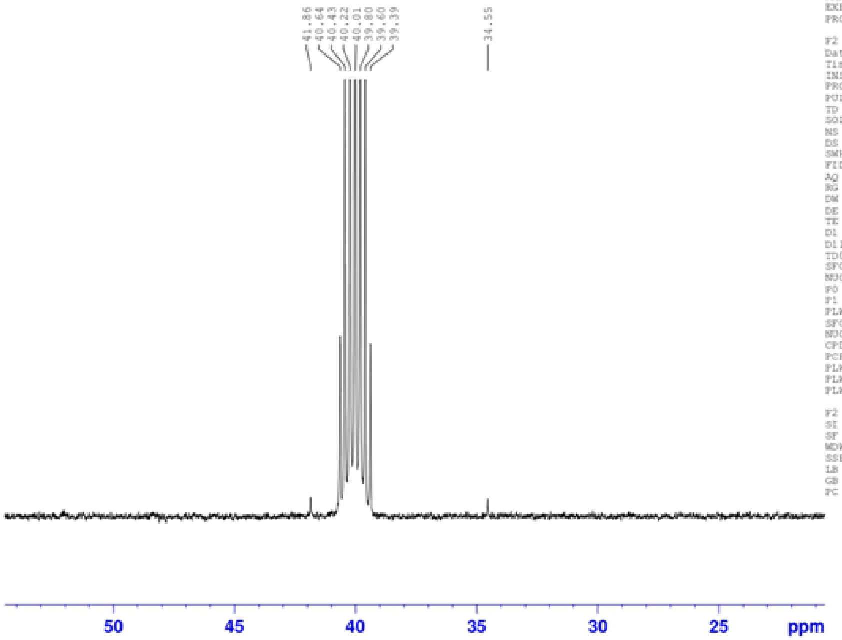

BRUKER

Current Data Parameters		
NAME	SNS FARHAT R PKJ BF C	
EXPNO	7	
PROCNO	1	
F2 - Acquisition Parameters		
Date_	20220707	
Time	19.23	h
INSTRUM	Avance	
PROBHD	Z8247_0054 (PH	
PULPROG	zgpg30	
TD	65536	
SOLVENT	DMSO	
NS	1024	
DS	4	
SWH	23809.523	Hz
FIDRES	0.726609	Hz
AQ	1.3762560	sec
RG	101	
DW	21.000	usec
DE	6.50	usec
TE	298.5	K
D1	2.00000000	sec
D11	0.03000000	sec
TD0	1	
SFO1	100.6228298	MHz
NUC1	13C	
P0	5.00	usec
P1	15.00	usec
PLW1	68.23799896	W
SFO2	400.1316005	MHz
NUC2	1H	
CPDPRG[2	waltz65	
PCPD2	90.00	usec
PLW2	14.19099998	W
PLW12	0.08298273	W
PLW13	0.04159095	W
F2 - Processing parameters		
SI	32768	
SF	100.6127685	MHz
WDW	EM	
SSB	0	
LB	1.00	Hz
GB	0	
PC	1.40	

SNS FARHAT R PKJ BF

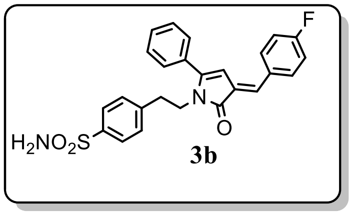

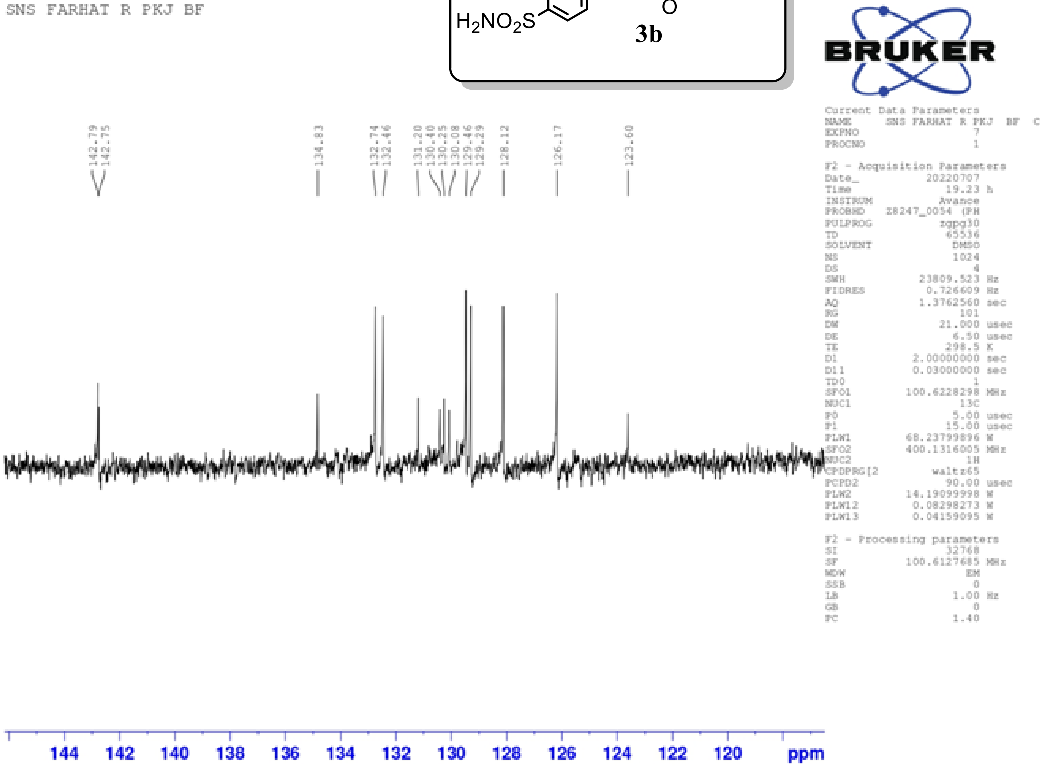

SNS FARHAT R PKJ BBr

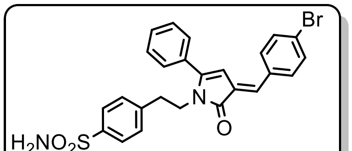

3d

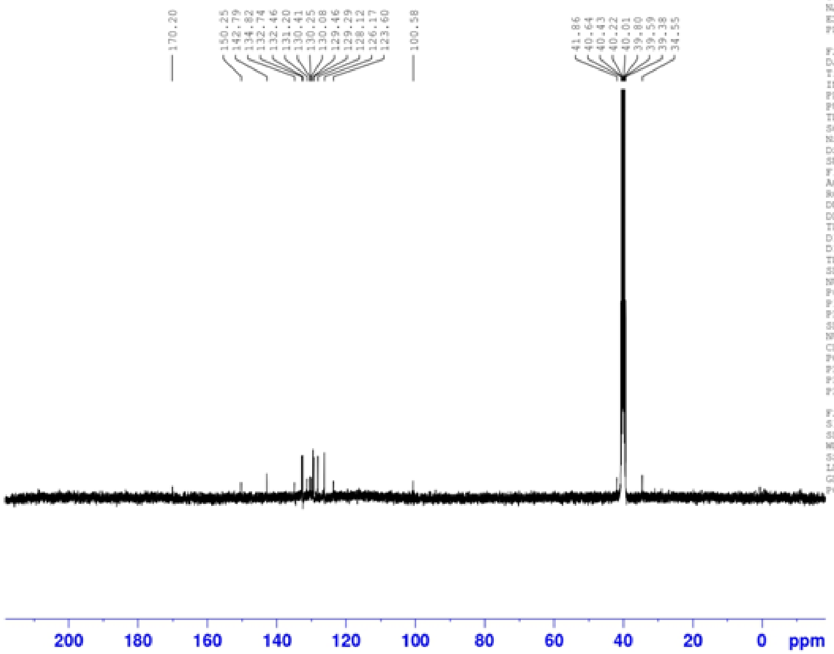

Current Data Parameters
NAME SNS FARHAT R PKJ BBr C
EXPNO 8
PROCNO 1

F2 - Acquisition Parameters
Date_ 20220723
Time 18.28 h
INSTRUM Avance
PROBHD Z8247_0054 (PH
PULPROG zgpg30
TD 65536
SOLVENT DMSO
NS 1024
DS 4
SWH 23809.523 Hz
FIDRES 0.726609 Hz
AQ 1.3762560 sec
RG 101
DW 21.000 usec
DE 6.50 usec
TE 298.2 K
D1 2.00000000 sec
D11 0.03000000 sec
TD0 1
SFO1 100.6228298 MHz
NUC1 13C
P0 5.00 usec
P1 15.00 usec
PLW1 68.23799896 W
SFO2 400.1316005 MHz
NUC2 1H
CPDPRG[2 waltz65
PCPD2 90.00 usec
PLW2 14.19099998 W
PLW12 0.08298273 W
PLW13 0.04159095 W

F2 - Processing parameters
SI 32768
SF 100.6127685 MHz
WDW EM
SSB 0
LB 1.00 Hz
GB 0
PC 1.40

SNS FARHAT R PKJ TBD

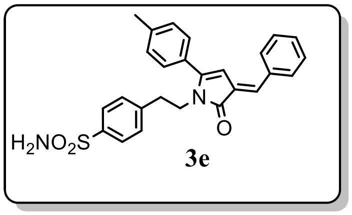

3e

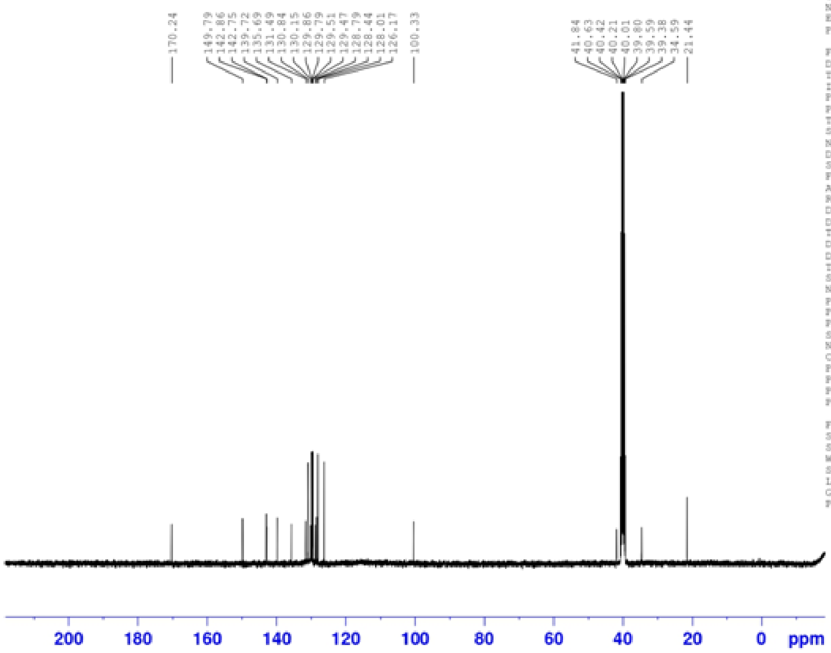


```
Current Data Parameters
NAME      SNS FARHAT R PKJ TBD  C
EXPNO                  1
PROCNO                 1

F2 - Acquisition Parameters
Date_           20220723
Time               19.44 h
INSTRUM           Avance
PROBHD   Z8247_0054 (PH
PULPROG           zgpg30
TD                 65536
SOLVENT             DMSO
NS                   717
DS                     4
SWH            23809.523 Hz
FIDRES          0.726609 Hz
AQ             1.3762560 sec
RG                   101
DW                21.000 usec
DE                  6.50 usec
TE                 298.2 K
D1            2.00000000 sec
D11           0.03000000 sec
TD0                    1
SFO1         100.6228298 MHz
NUC1                 13C
P0                  5.00 usec
P1                 15.00 usec
PLW1         68.23799896 W
SFO2         400.1316005 MHz
NUC2                  1H
CPDPRG[2         waltz65
PCPD2              90.00 usec
PLW2         14.19099998 W
PLW12         0.08298273 W
PLW13         0.04159095 W

F2 - Processing parameters
SI                 32768
SF           100.6127685 MHz
WDW                   EM
SSB                    0
LB                  1.00 Hz
GB                     0
PC                  1.40
```

SNS FARHAT R PKJ TBD

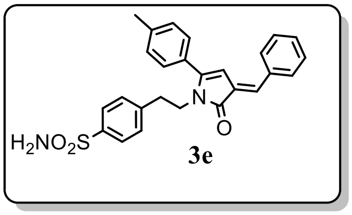

3e

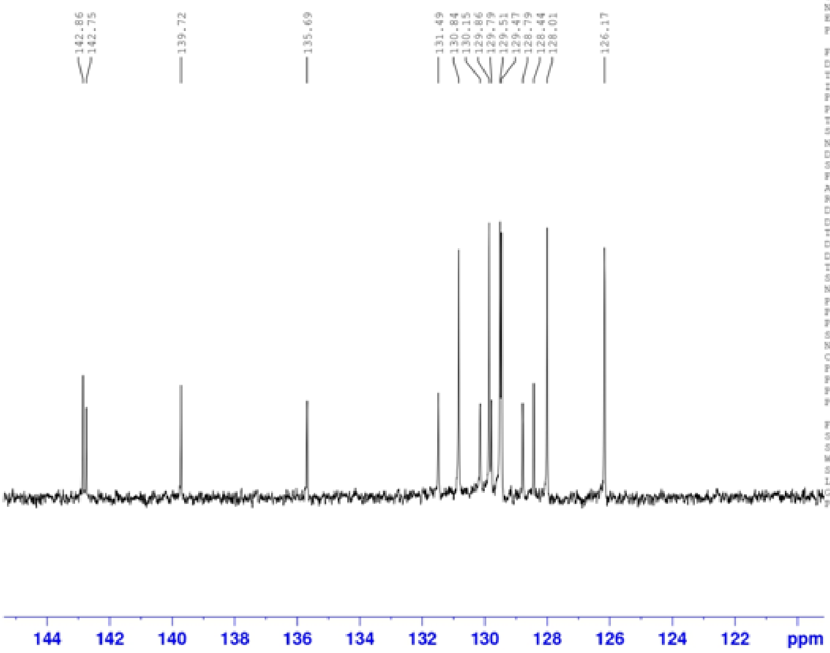

Current Data Parameters
NAME SNS FARHAT R PKJ TBD C
EXPNO 1
PROCNO 1

F2 - Acquisition Parameters
Date_ 20220723
Time 19.44 h
INSTRUM Avance
PROBHD Z8247_0054 (PH
PULPROG zgpg30
TD 65536
SOLVENT DMSO
NS 717
DS 4
SWH 23809.523 Hz
FIDRES 0.726609 Hz
AQ 1.3762560 sec
RG 101
DW 21.000 usec
DE 6.50 usec
TE 298.2 K
D1 2.00000000 sec
D11 0.03000000 sec
TD0 1
SFO1 100.6228298 MHz
NUC1 13C
P0 5.00 usec
P1 15.00 usec
PLW1 68.23799896 W
SFO2 400.1316005 MHz
NUC2 1H
CPDPRG[2 waltz65
PCPD2 90.00 usec
PLW2 14.19099998 W
PLW12 0.08298273 W
PLW13 0.04159095 W

F2 - Processing parameters
SI 32768
SF 100.6127685 MHz
WDW EM
SSB 0
LB 1.00 Hz
GB 0
PC 1.40

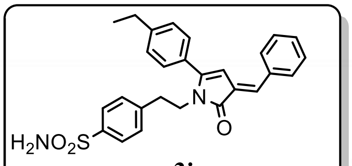

H_2NO_2S
N
O

3i

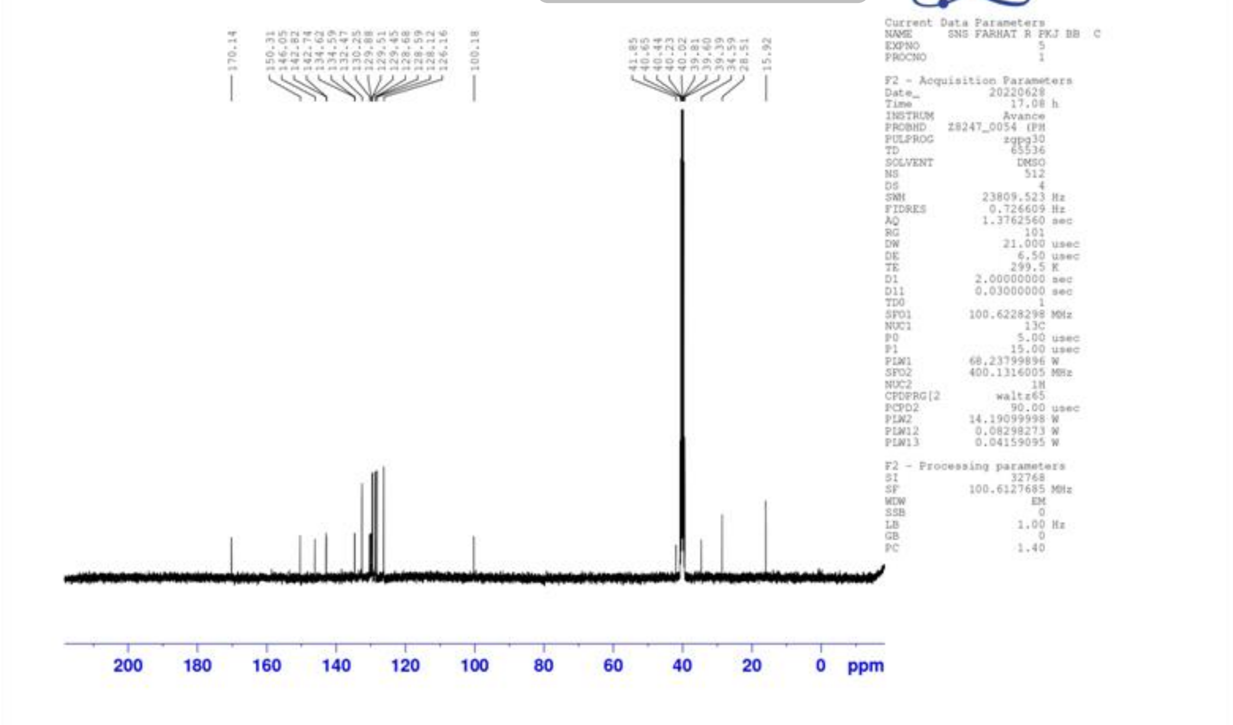

SNS FARHAT R PKJ BB
BRUKER
170.14
150.31
146.05
142.82
142.74
134.62
134.59
132.47
130.25
129.88
129.51
129.45
128.68
128.59
128.12
126.16
100.18
41.85
40.65
40.44
40.23
40.02
39.81
39.60
39.39
34.59
28.51
15.92
Current Data Parameters
NAME SNS FARHAT R PKJ BB C
EXPNO 5
PROCNO 1
F2 - Acquisition Parameters
Date_ 20220628
Time 17.08 h
INSTRUM Avance
PROBHD Z8247_0054 (PH
PULPROG zgpg30
TD 65536
SOLVENT DMSO
NS 512
DS 4
SWH 23809.523 Hz
FIDRES 0.726609 Hz
AQ 1.3762560 sec
RG 101
DW 21.000 usec
DE 6.50 usec
TE 299.5 K
D1 2.00000000 sec
D11 0.03000000 sec
TD0 1
SFO1 100.6228298 MHz
NUC1 13C
P0 5.00 usec
P1 15.00 usec
PLW1 68.23799896 W
SFO2 400.1316005 MHz
NUC2 1H
CPDPRG[2 waltz65
PCPD2 90.00 usec
PLW2 14.19099998 W
PLW12 0.08298273 W
PLW13 0.04159095 W
F2 - Processing parameters
SI 32768
SF 100.6127685 MHz
WDW EM
SSB 0
LB 1.00 Hz
GB 0
PC 1.40
200
180
160
140
120
100
80
60
40
20
0
ppm

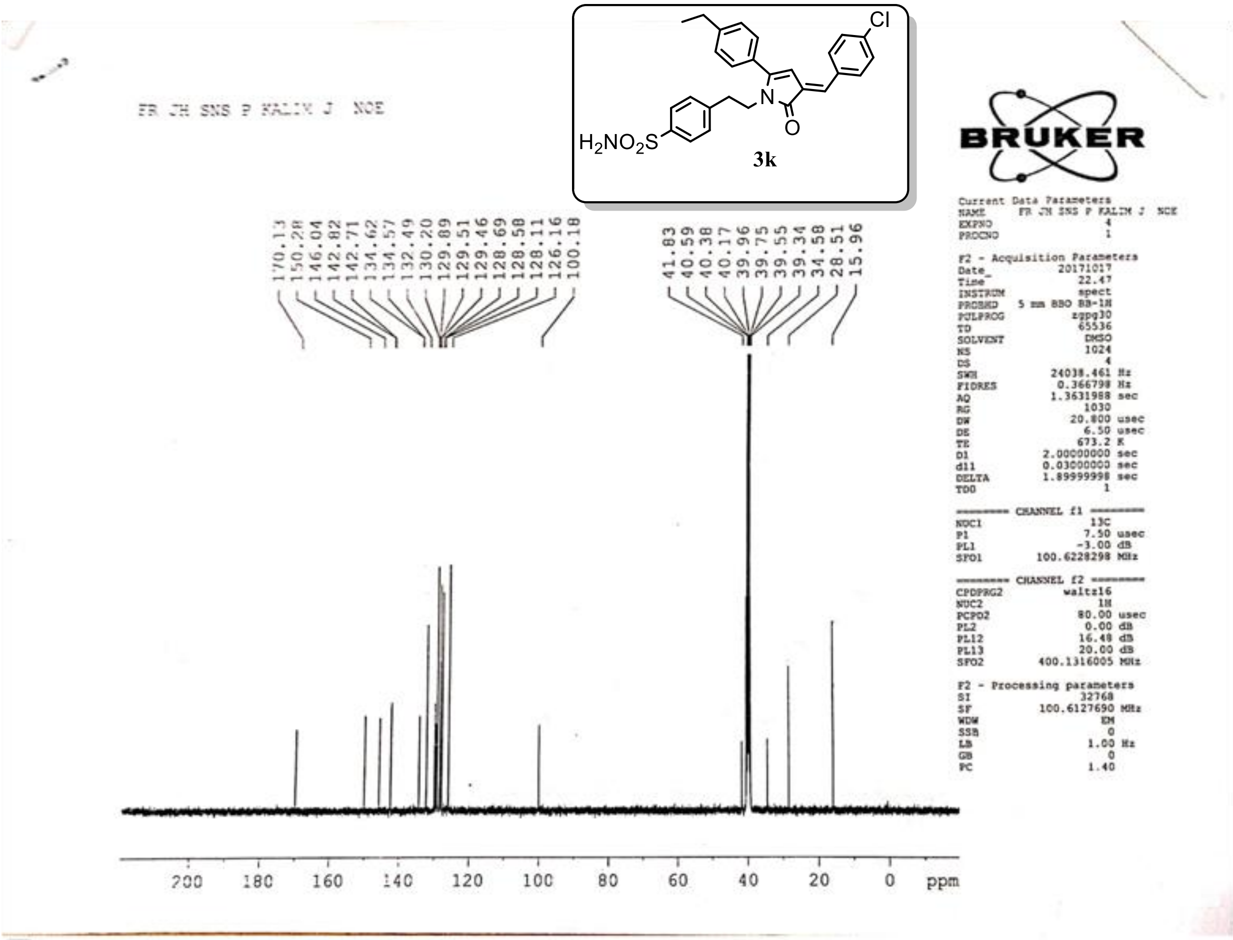

CS Scanned with CamScanner

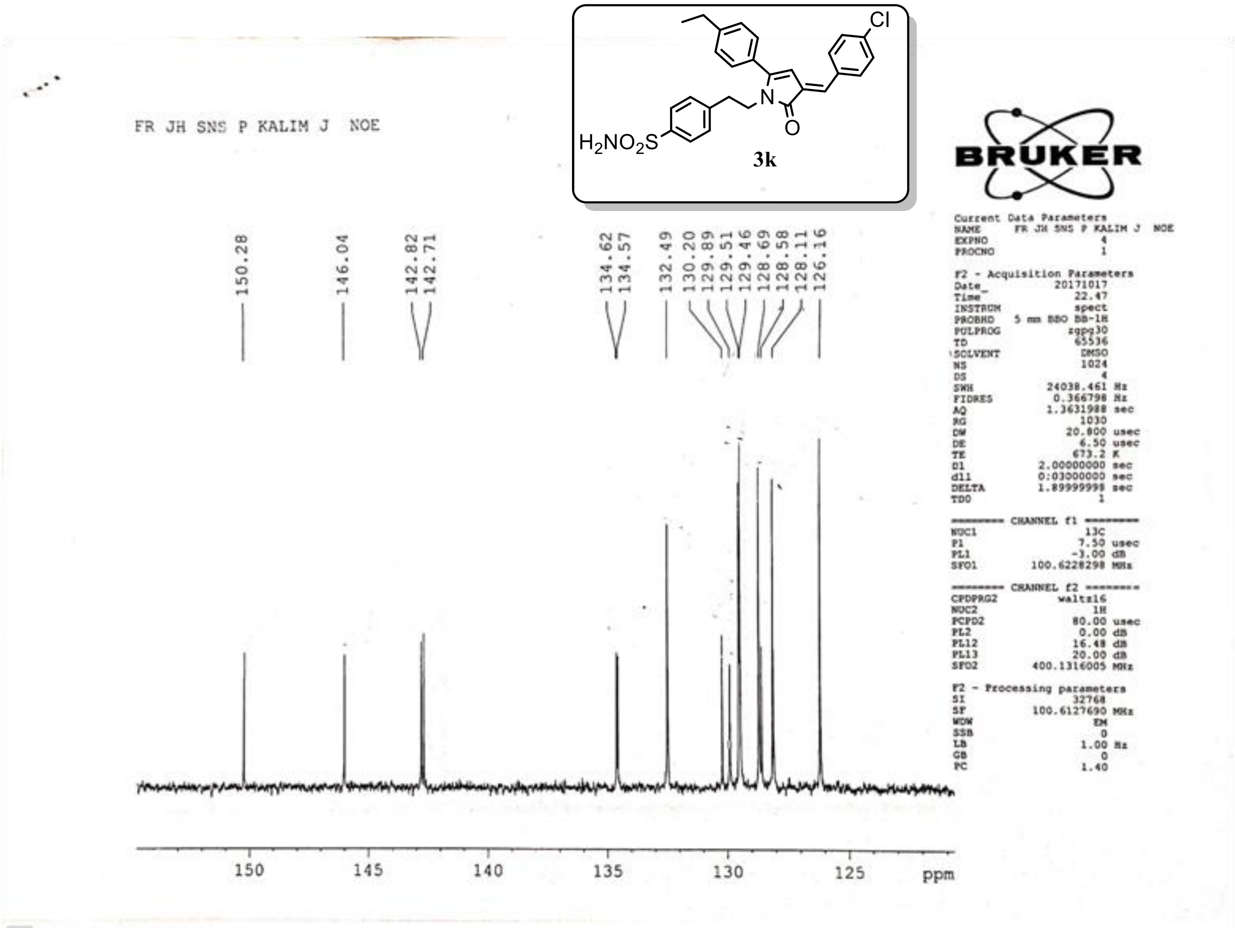

Scanned with CamScanner

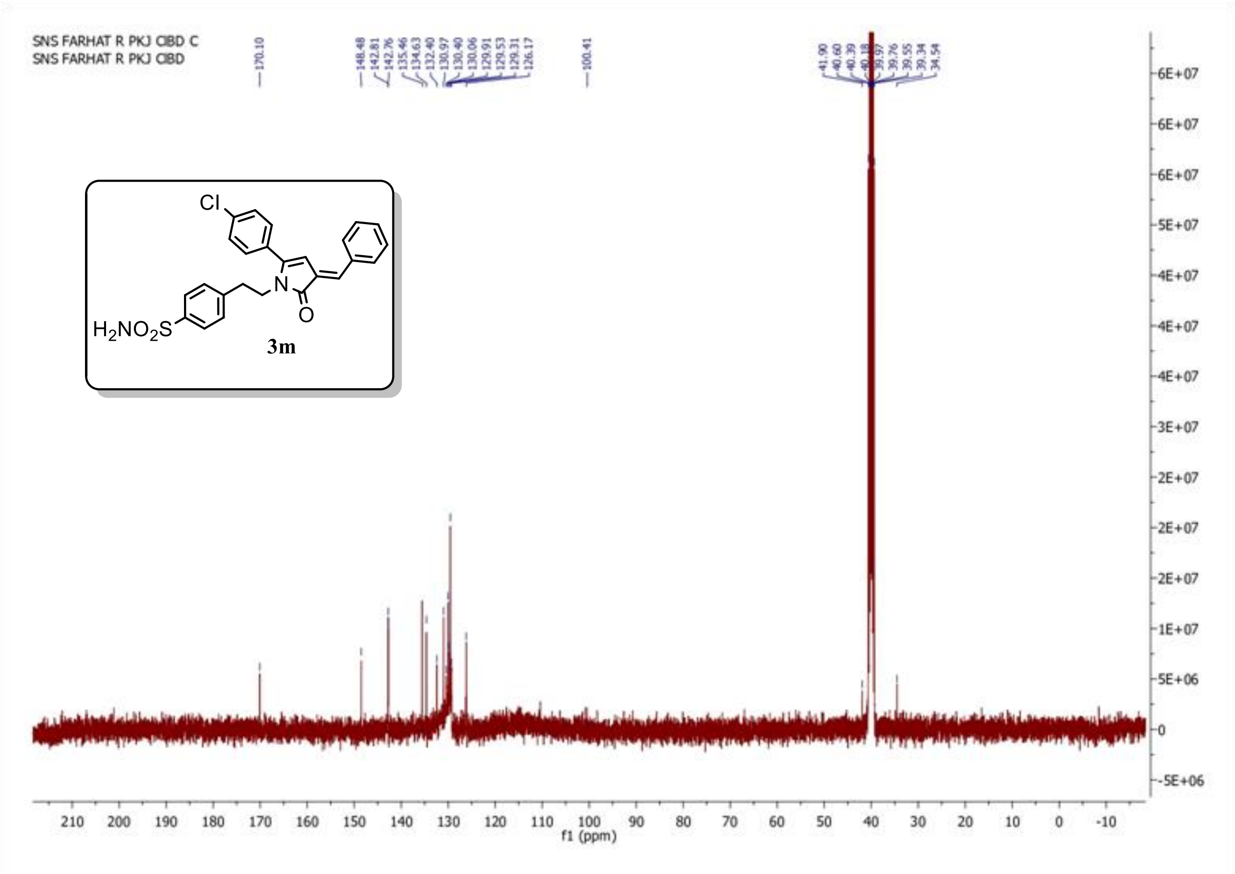
SNS FARHAT R PKJ CIBD C
SNS FARHAT R PKJ CIBD
170.10
148.48
142.81
142.76
135.46
134.63
132.40
130.97
130.40
130.06
129.91
129.53
129.31
126.17
100.41
41.90
40.60
40.39
40.18
39.97
39.76
39.55
39.34
34.54
Cl
N
O
H_2NO_2S
3m
f1 (ppm)

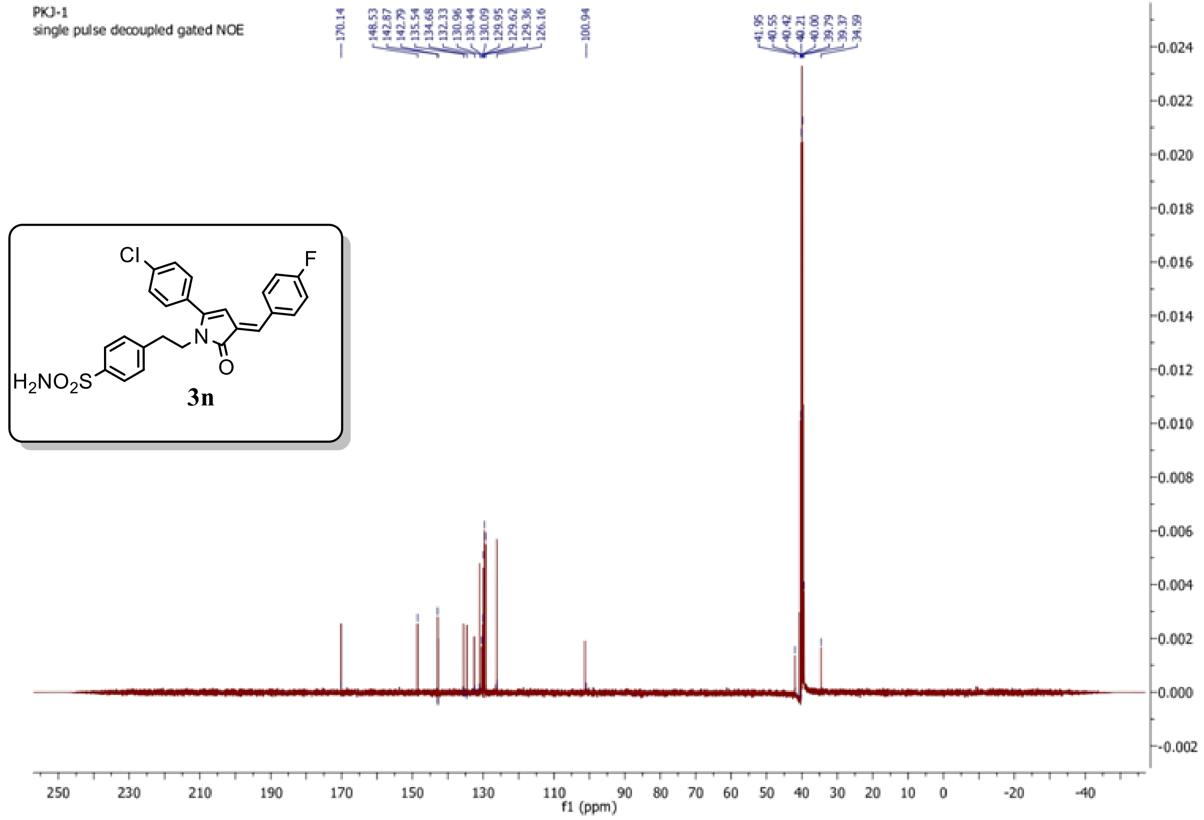
PKJ-1
single pulse decoupled gated NOE
170.14
148.53
142.87
142.79
135.54
134.68
132.33
130.96
130.44
130.09
129.95
129.62
129.36
126.16
100.94
41.95
40.55
40.42
40.21
40.00
39.79
39.37
34.59
3n
f1 (ppm)

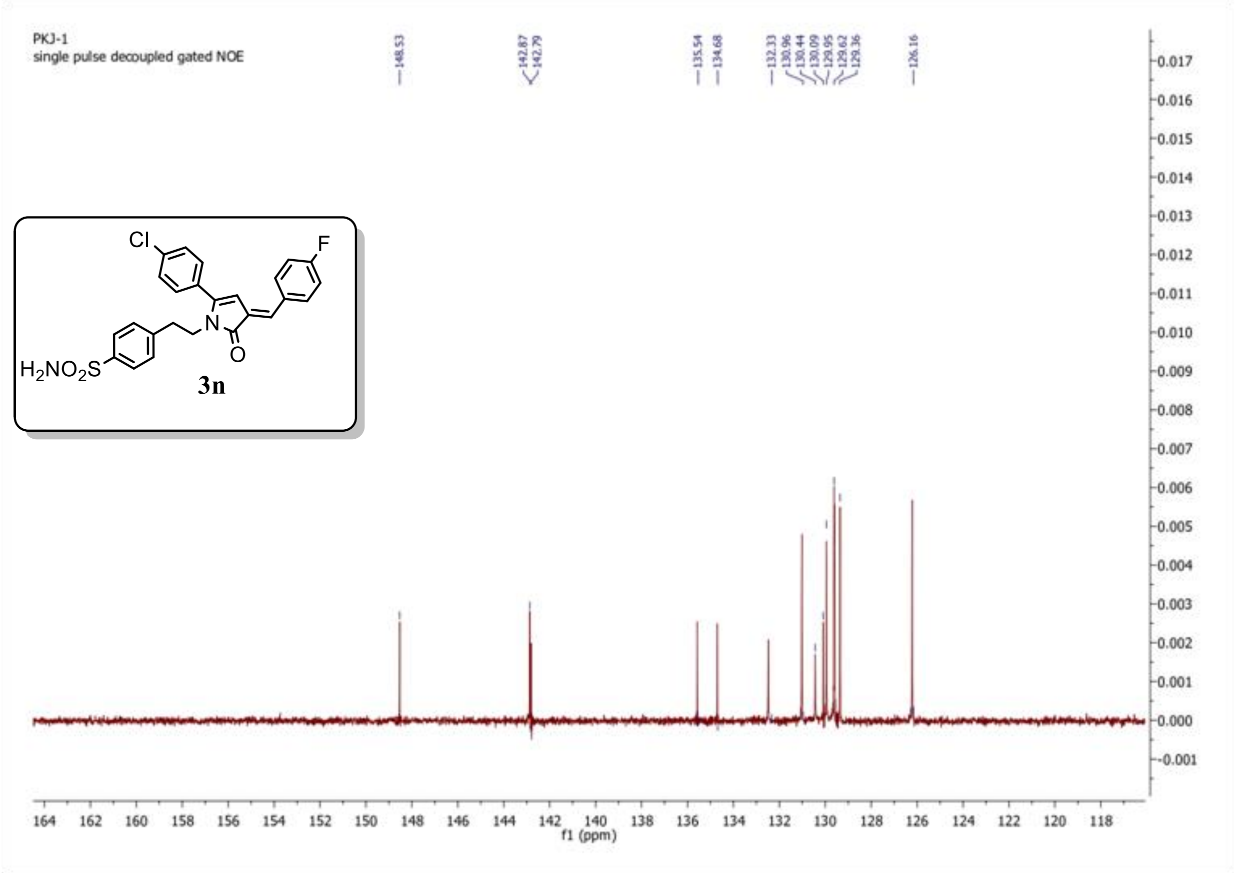

Mass spectra

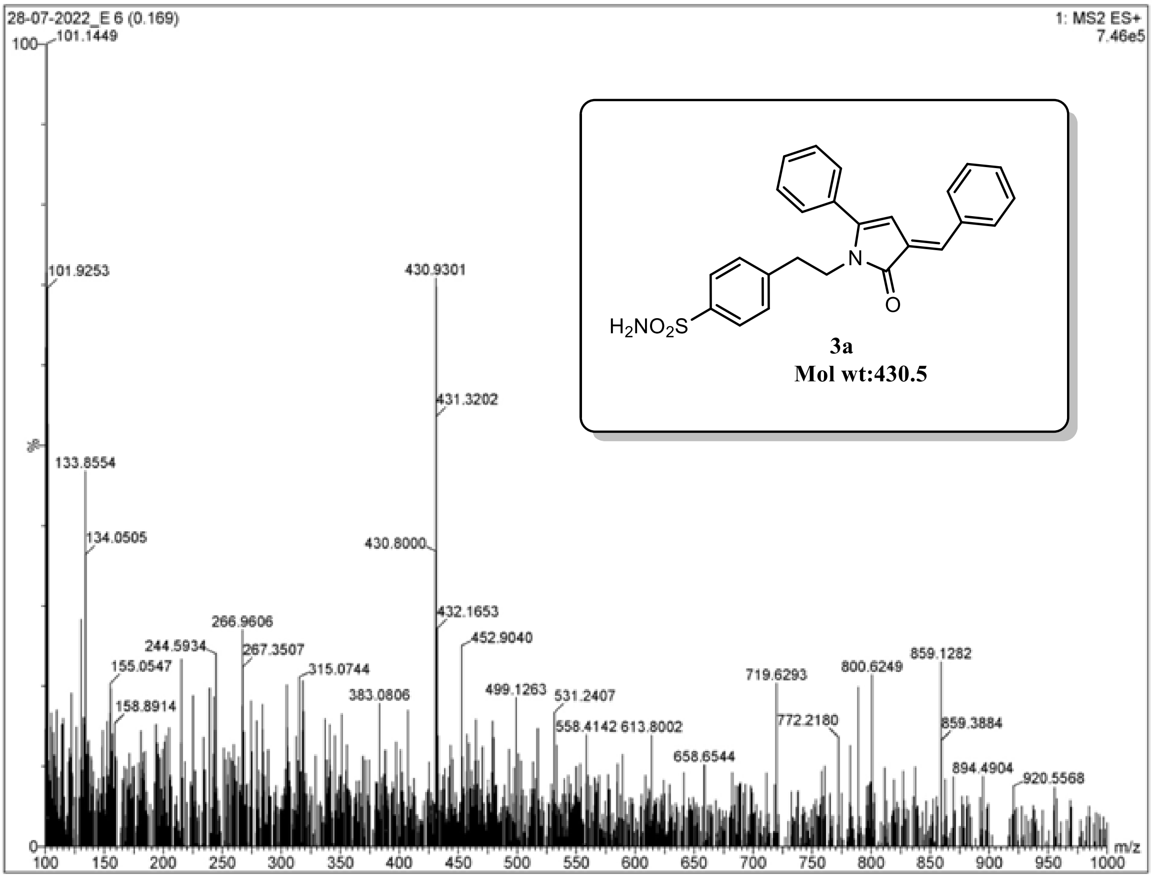

28-07-2022_E 6 (0.169)
1: MS2 ES+
7.46e5
101.1449
101.9253
133.8554
134.0505
155.0547
158.8914
244.5934
266.9606
267.3507
315.0744
383.0806
430.9301
431.3202
430.8000
432.1653
452.9040
499.1263
531.2407
558.4142
613.8002
658.6544
719.6293
772.2180
800.6249
859.1282
859.3884
894.4904
920.5568
m/z
%
H_2NO_2S
N
O
3a
Mol wt:430.5

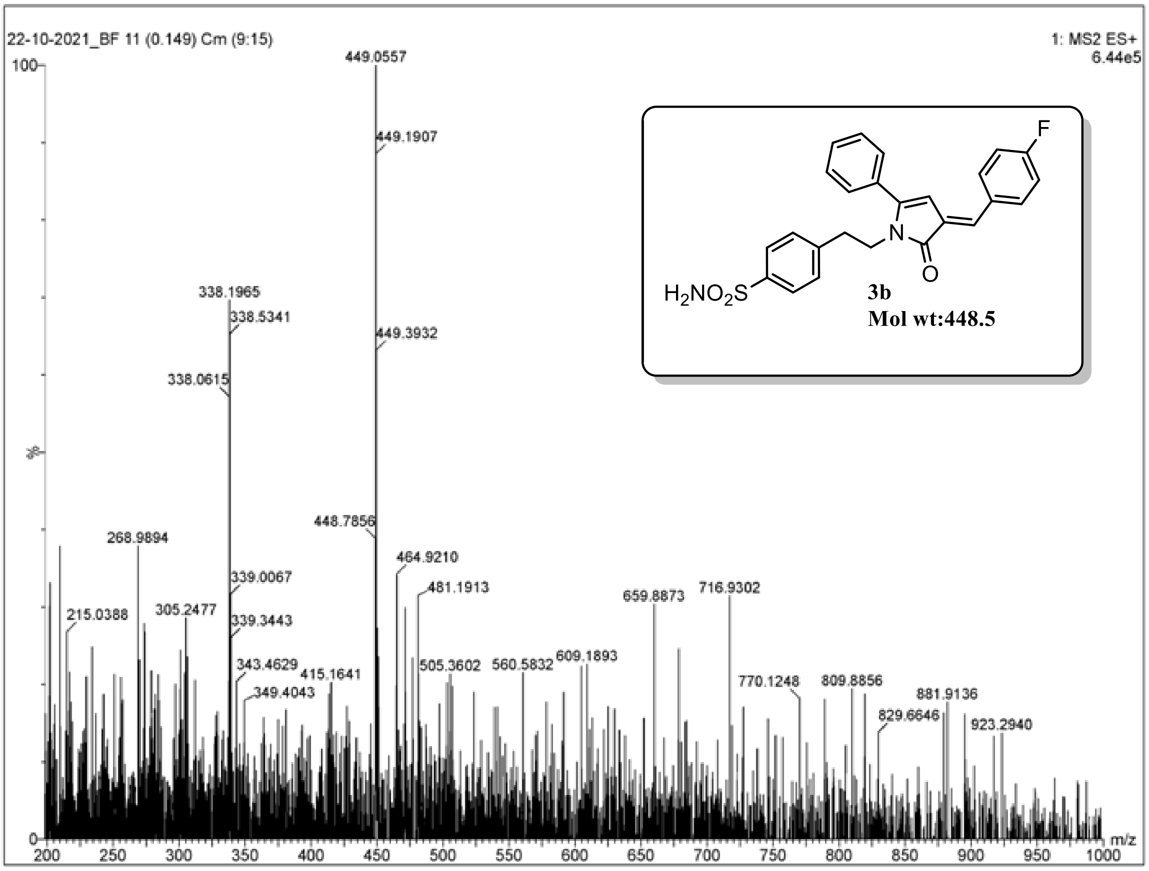
22-10-2021_BF 11 (0.149) Cm (9:15)
1: MS2 ES+
6.44e5
449.0557
449.1907
338.1965
338.5341
449.3932
338.0615
448.7856
268.9894
464.9210
339.0067
481.1913
659.8873
716.9302
215.0388
305.2477
339.3443
609.1893
343.4629
415.1641
505.3602
560.5832
770.1248
809.8856
349.4043
881.9136
829.6646
923.2940
m/z
H2NO2S
F
N
O
3b
Mol wt:448.5

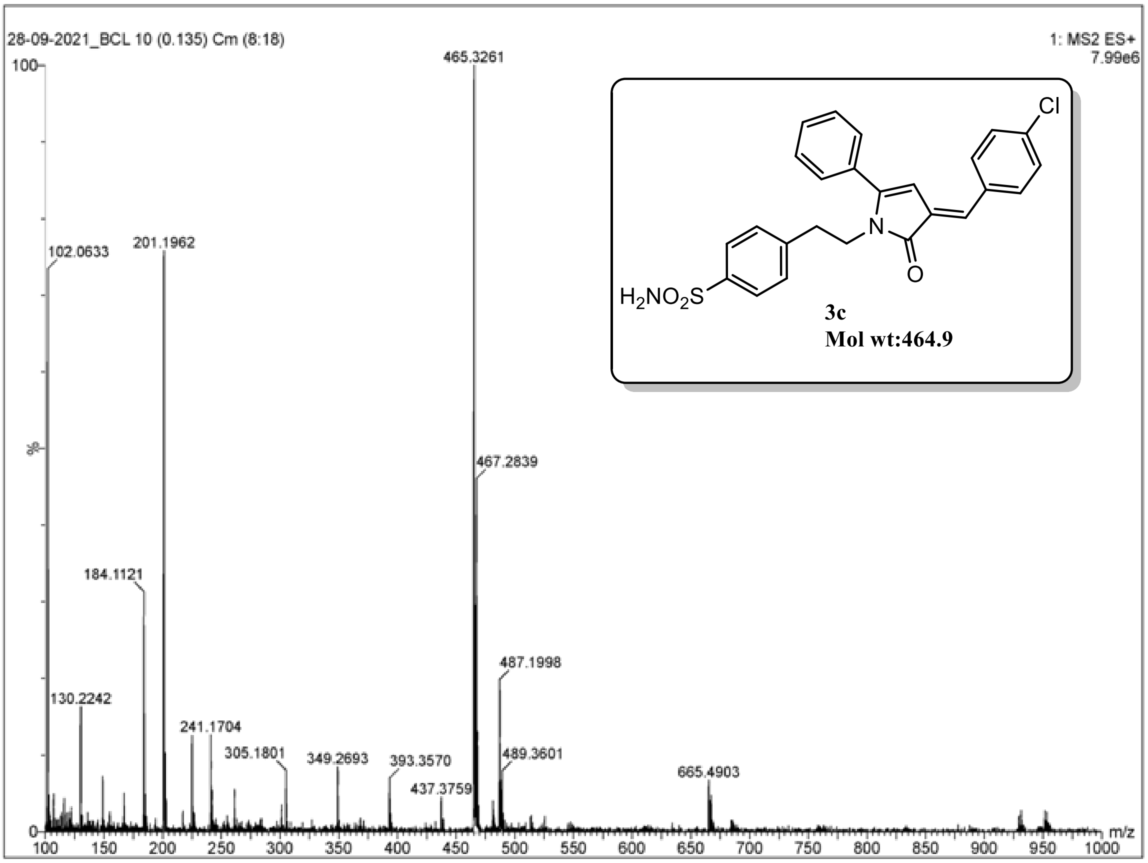
28-09-2021_BCL 10 (0.135) Cm (8:18)
1: MS2 ES+
7.99e6
100
%
0
465.3261
102.0633
201.1962
467.2839
184.1121
487.1998
130.2242
241.1704
305.1801
349.2693
393.3570
489.3601
437.3759
665.4903
m/z
100 150 200 250 300 350 400 450 500 550 600 650 700 750 800 850 900 950 1000
Cl
N
O
H_2NO_2S
3c
Mol wt:464.9

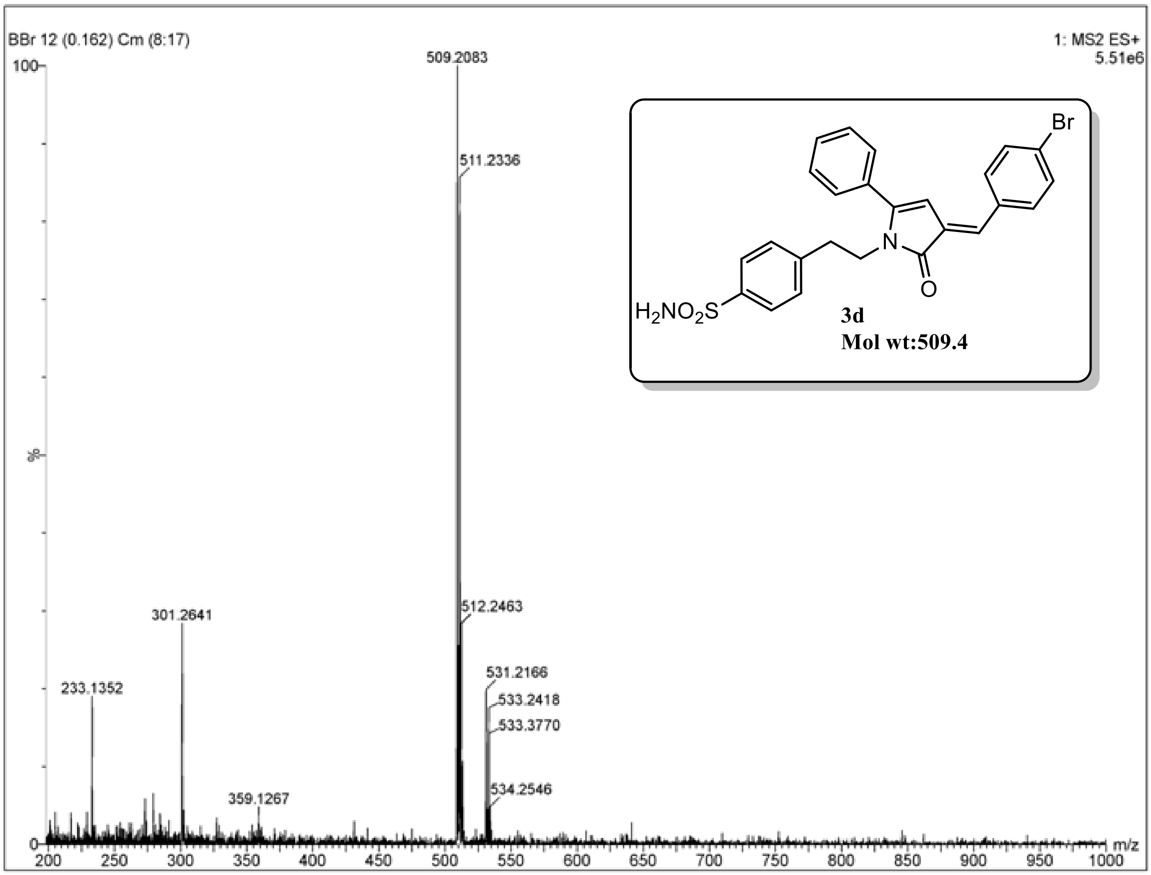
BBr 12 (0.162) Cm (8:17)
1: MS2 ES+
5.51e6
509.2083
511.2336
512.2463
531.2166
533.2418
533.3770
534.2546
301.2641
233.1352
359.1267
100
%
0
m/z
200 250 300 350 400 450 500 550 600 650 700 750 800 850 900 950 1000
Br
N
O
H_2NO_2S
3d
Mol wt:509.4

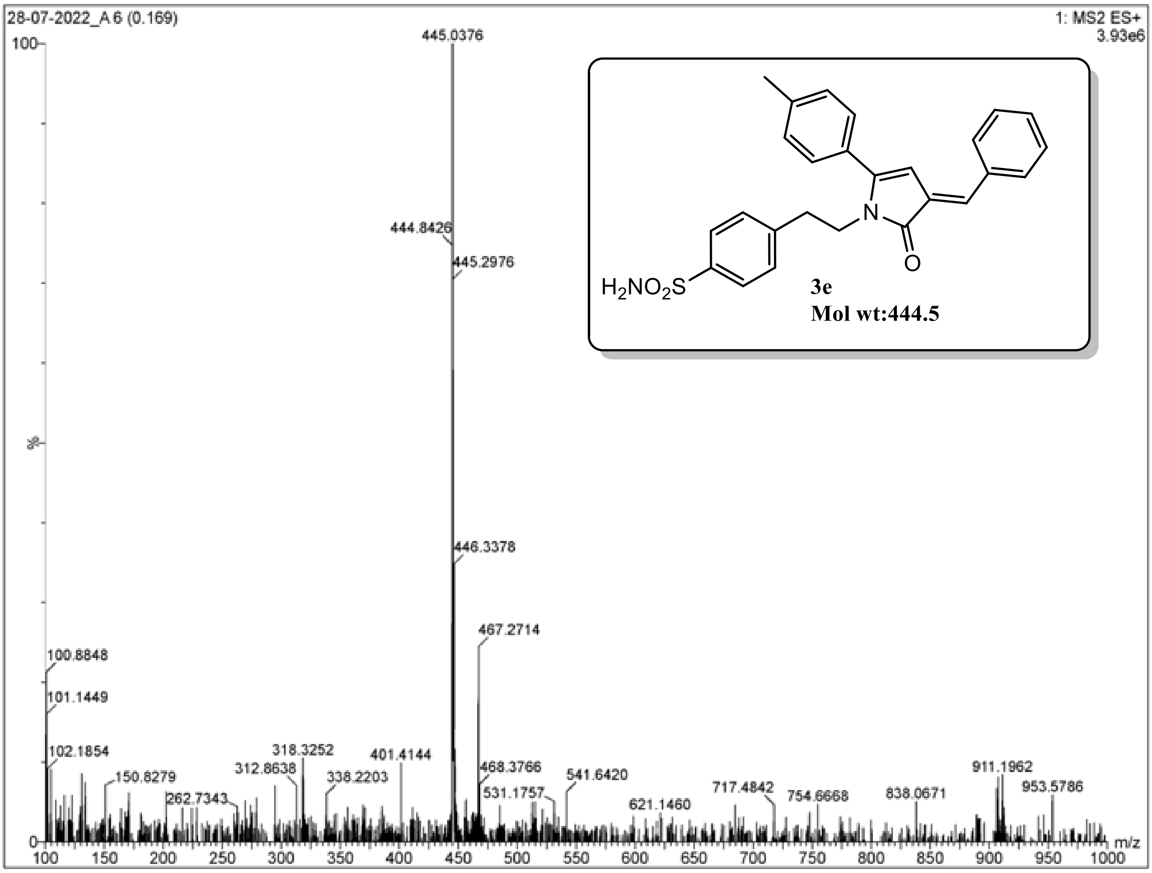
28-07-2022_A 6 (0.169)
1: MS2 ES+
3.93e6
445.0376
444.8426
445.2976
446.3378
467.2714
100.8848
101.1449
102.1854
150.8279
262.7343
312.8638
318.3252
338.2203
401.4144
468.3766
531.1757
541.6420
621.1460
717.4842
754.6668
838.0671
911.1962
953.5786
%
m/z
H_2NO_2S
N
O
3e
Mol wt:444.5

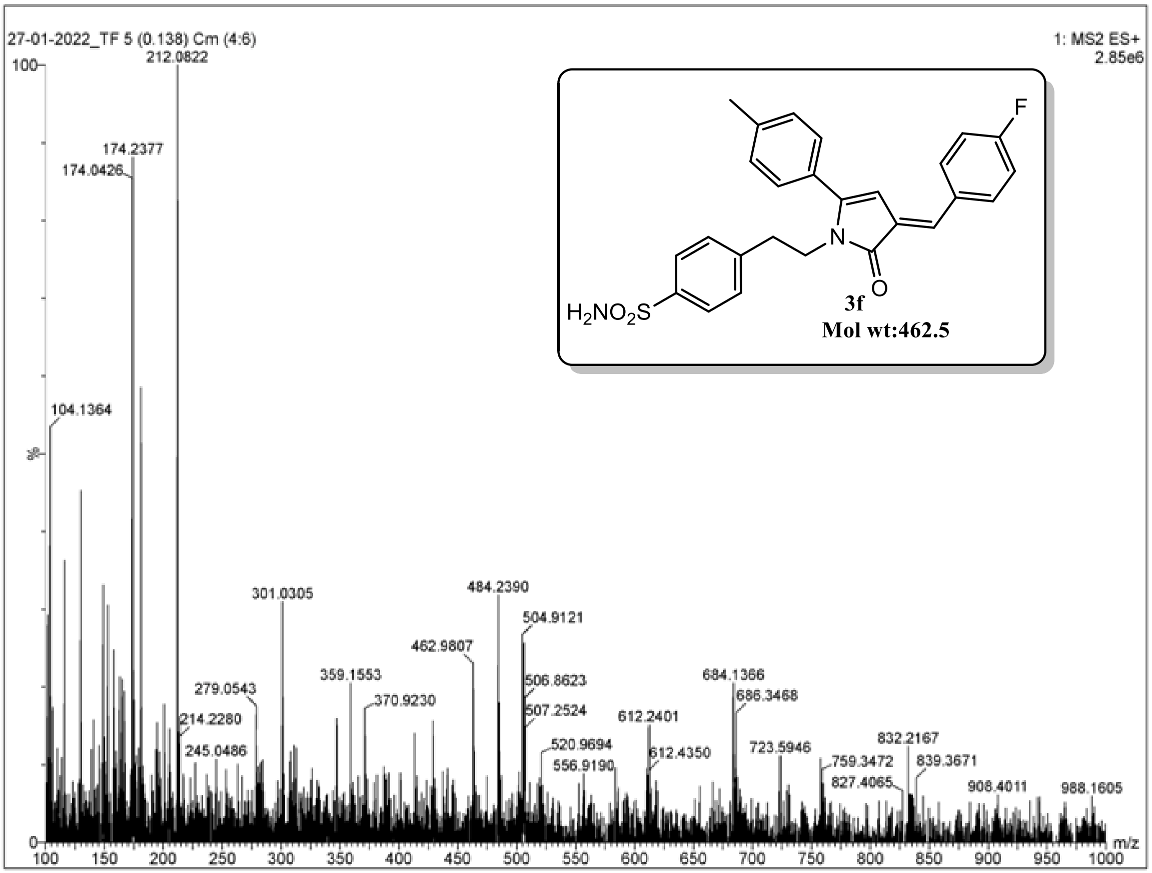
27-01-2022_TF 5 (0.138) Cm (4:6)
1: MS2 ES+
2.85e6
212.0822
174.2377
174.0426
104.1364
301.0305
484.2390
504.9121
462.9807
359.1553
506.8623
279.0543
370.9230
507.2524
214.2280
612.2401
684.1366
686.3468
245.0486
520.9694
556.9190
612.4350
723.5946
832.2167
759.3472
839.3671
827.4065
908.4011
988.1605
%
m/z
H2NO2S
F
N
O
3f
Mol wt:462.5

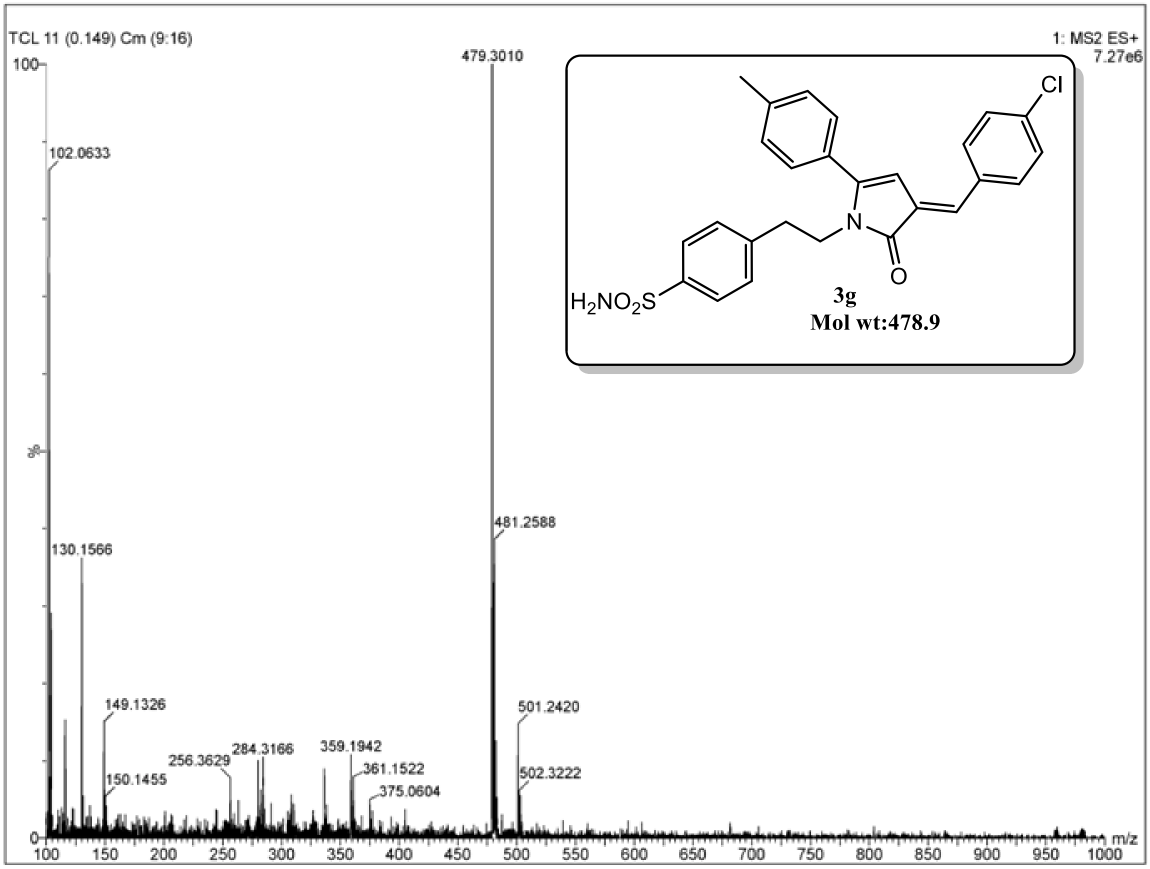

TCL 11 (0.149) Cm (9:16)
1: MS2 ES+
7.27e6
479.3010
102.0633
130.1566
149.1326
150.1455
256.3629
284.3166
359.1942
361.1522
375.0604
481.2588
501.2420
502.3222
%
m/z
Cl
N
O
H_2NO_2S
3g
Mol wt:478.9

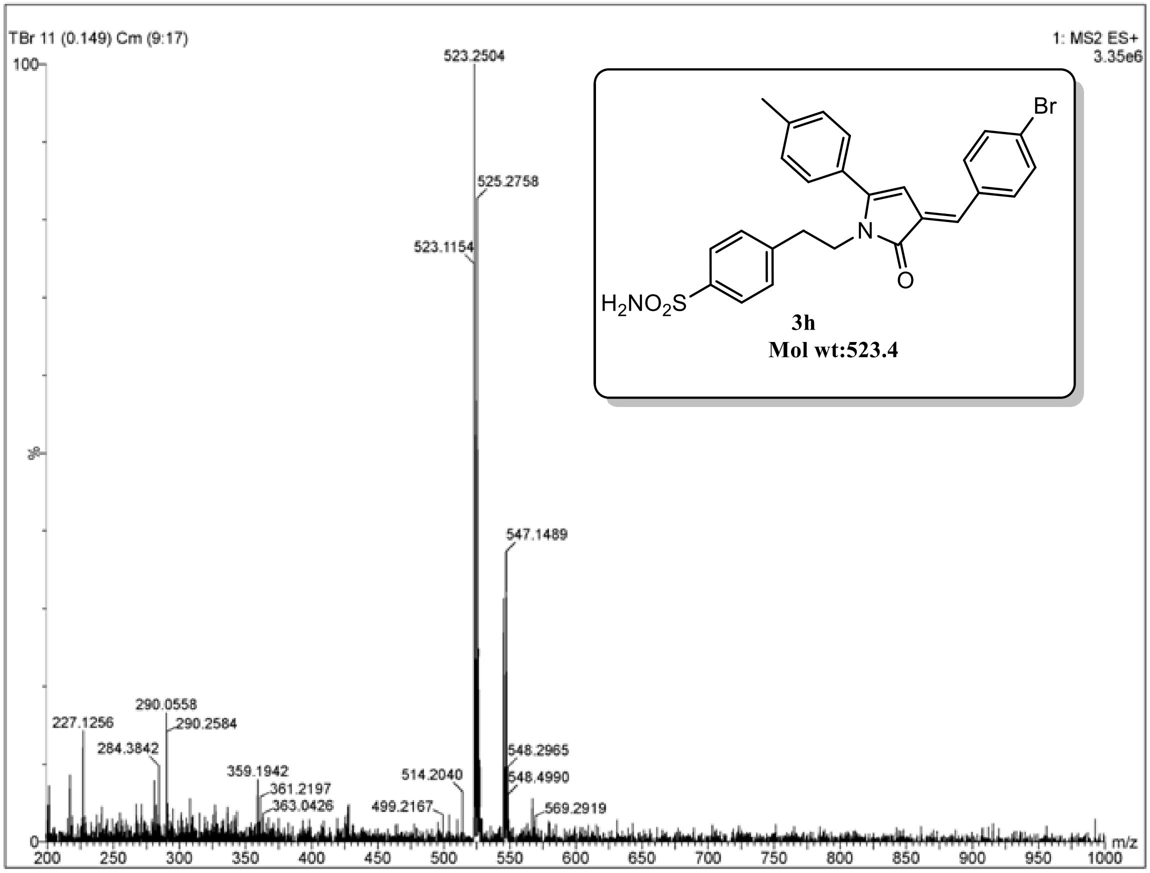
TBr 11 (0.149) Cm (9:17)
1: MS2 ES+
3.35e6
523.2504
525.2758
523.1154
547.1489
227.1256
290.0558
290.2584
284.3842
359.1942
361.2197
363.0426
514.2040
499.2167
548.2965
548.4990
569.2919
%
m/z
Br
N
O
H2NO2S
3h
Mol wt:523.4

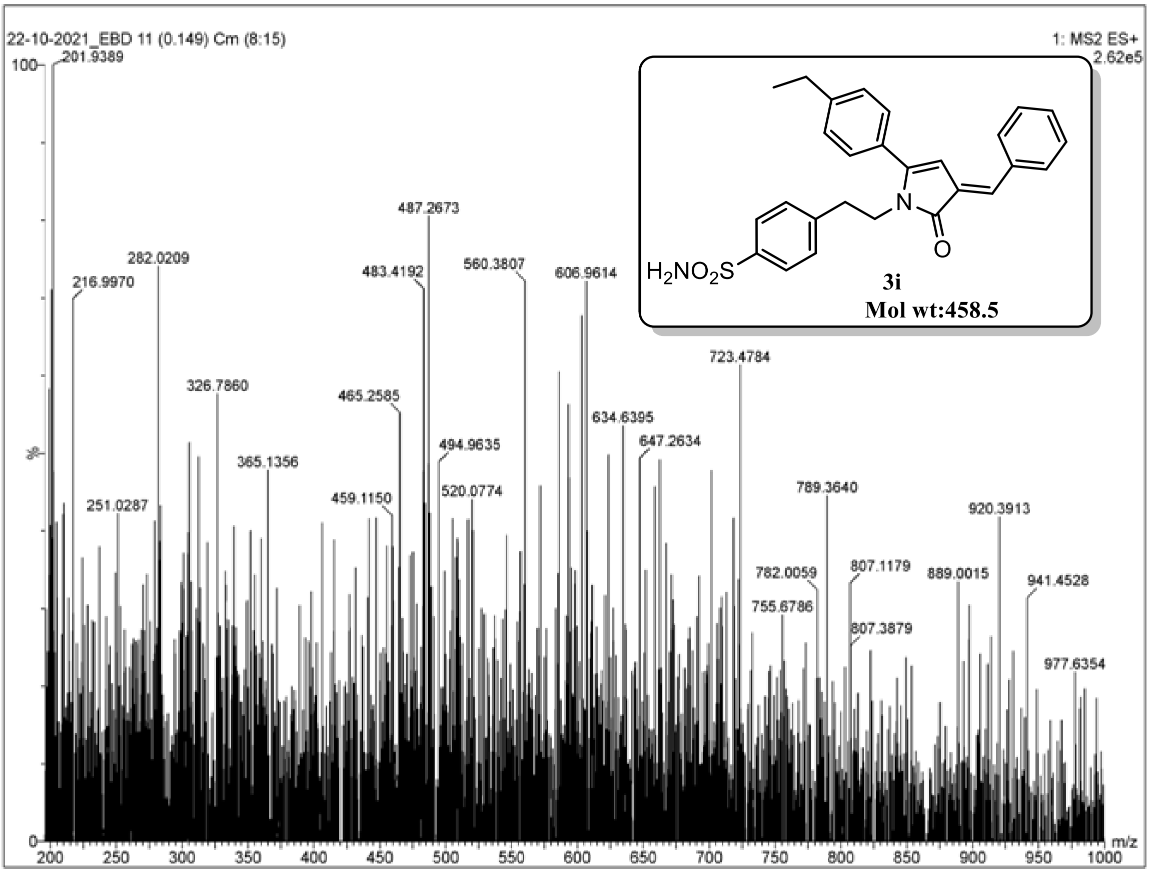
22-10-2021_EBD 11 (0.149) Cm (8:15)
1: MS2 ES+
2.62e5
201.9389
216.9970
251.0287
282.0209
326.7860
365.1356
459.1150
465.2585
483.4192
487.2673
494.9635
520.0774
560.3807
606.9614
634.6395
647.2634
723.4784
755.6786
782.0059
789.3640
807.1179
807.3879
889.0015
920.3913
941.4528
977.6354
%
m/z
H_2NO_2S
N
O
3i
Mol wt:458.5

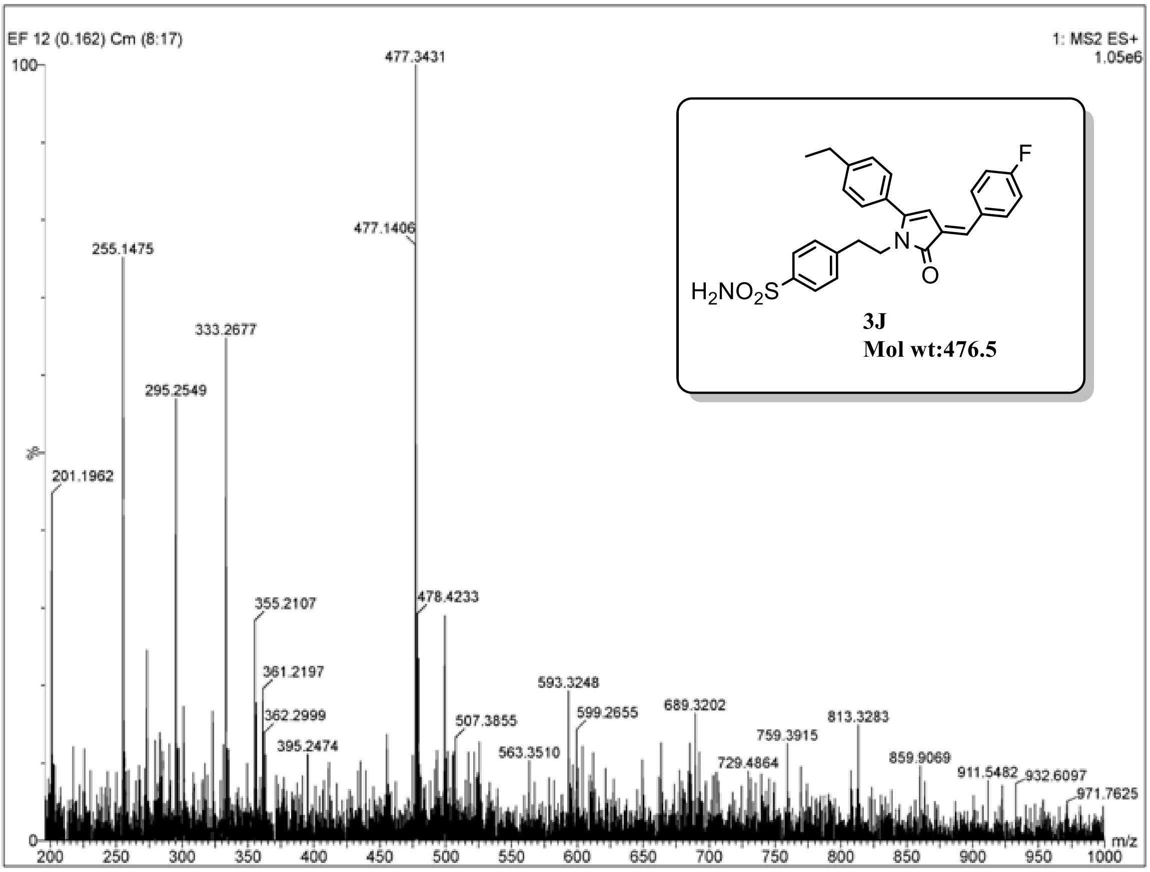
EF 12 (0.162) Cm (8:17)
1: MS2 ES+
1.05e6
100
%
0
477.3431
477.1406
255.1475
333.2677
295.2549
201.1962
355.2107
478.4233
361.2197
362.2999
395.2474
507.3855
563.3510
593.3248
599.2655
689.3202
729.4864
759.3915
813.3283
859.9069
911.5482
932.6097
971.7625
m/z
200 250 300 350 400 450 500 550 600 650 700 750 800 850 900 950 1000
F
N
O
H_2NO_2S
3J
Mol wt:476.5

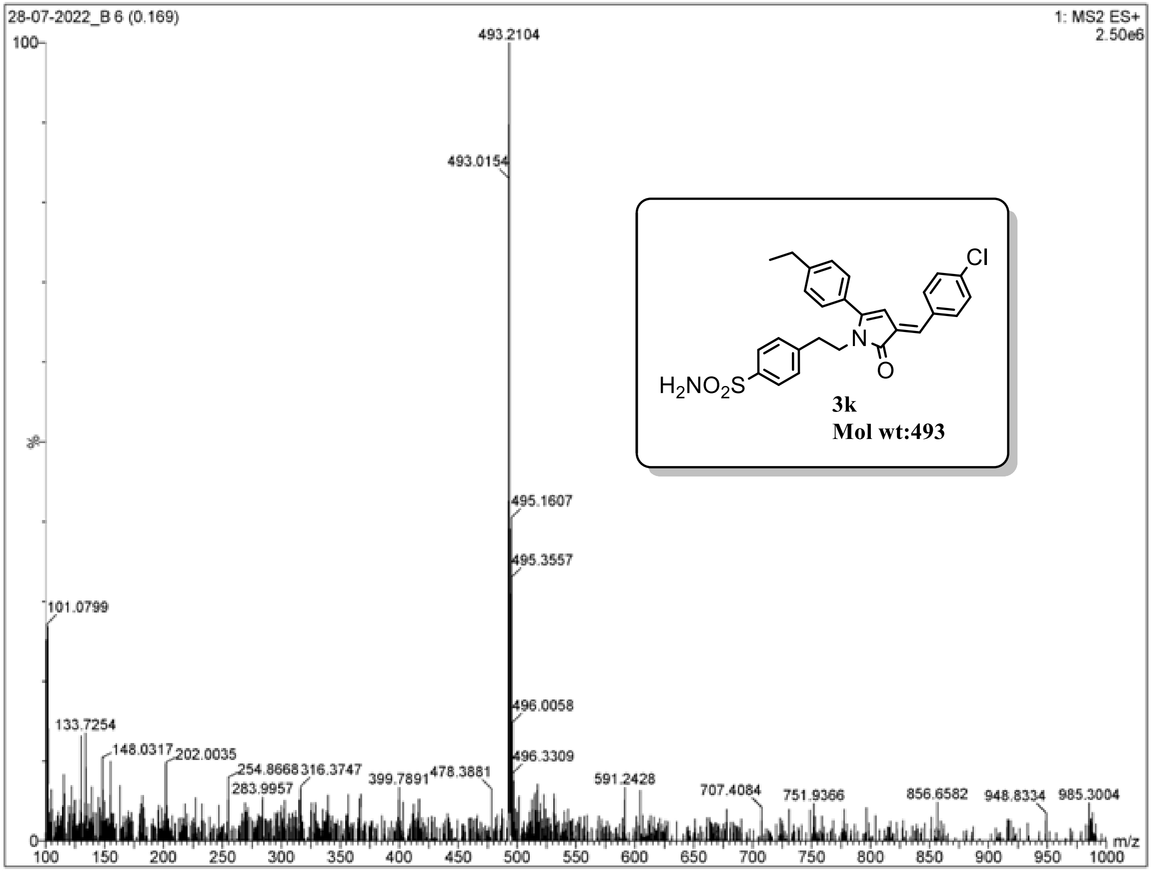
28-07-2022_B 6 (0.169)
1: MS2 ES+
2.50e6
100
%
0
493.2104
493.0154
495.1607
495.3557
101.0799
496.0058
133.7254
148.0317
202.0035
254.8668
316.3747
283.9957
399.7891
478.3881
496.3309
591.2428
707.4084
751.9366
856.6582
948.8334
985.3004
m/z
100 150 200 250 300 350 400 450 500 550 600 650 700 750 800 850 900 950 1000
Cl
N
O
H_2NO_2S
3k
Mol wt:493

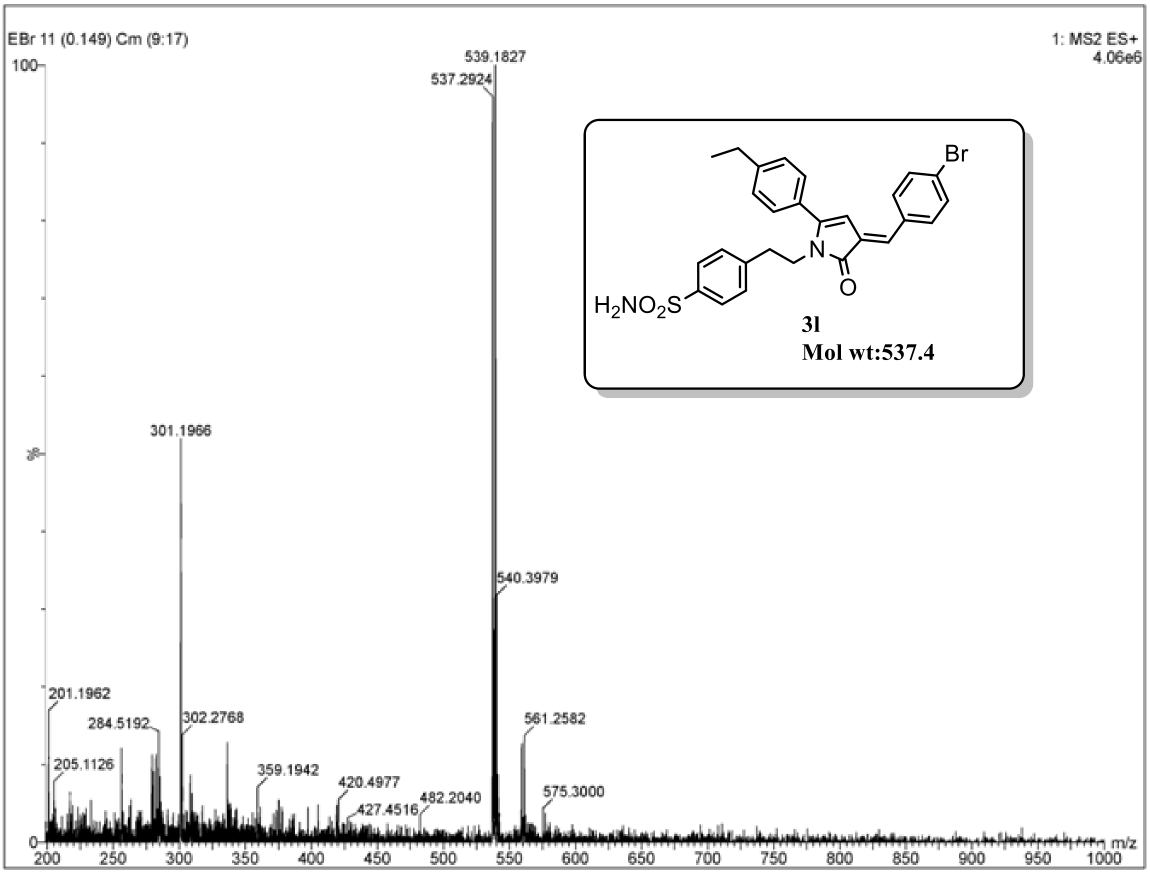
EBr 11 (0.149) Cm (9:17)
1: MS2 ES+
4.06e6
539.1827
537.2924
301.1966
540.3979
201.1962
284.5192
302.2768
205.1126
359.1942
420.4977
427.4516
482.2040
561.2582
575.3000
%
m/z
Br
N
O
H_2NO_2S
3l
Mol wt:537.4

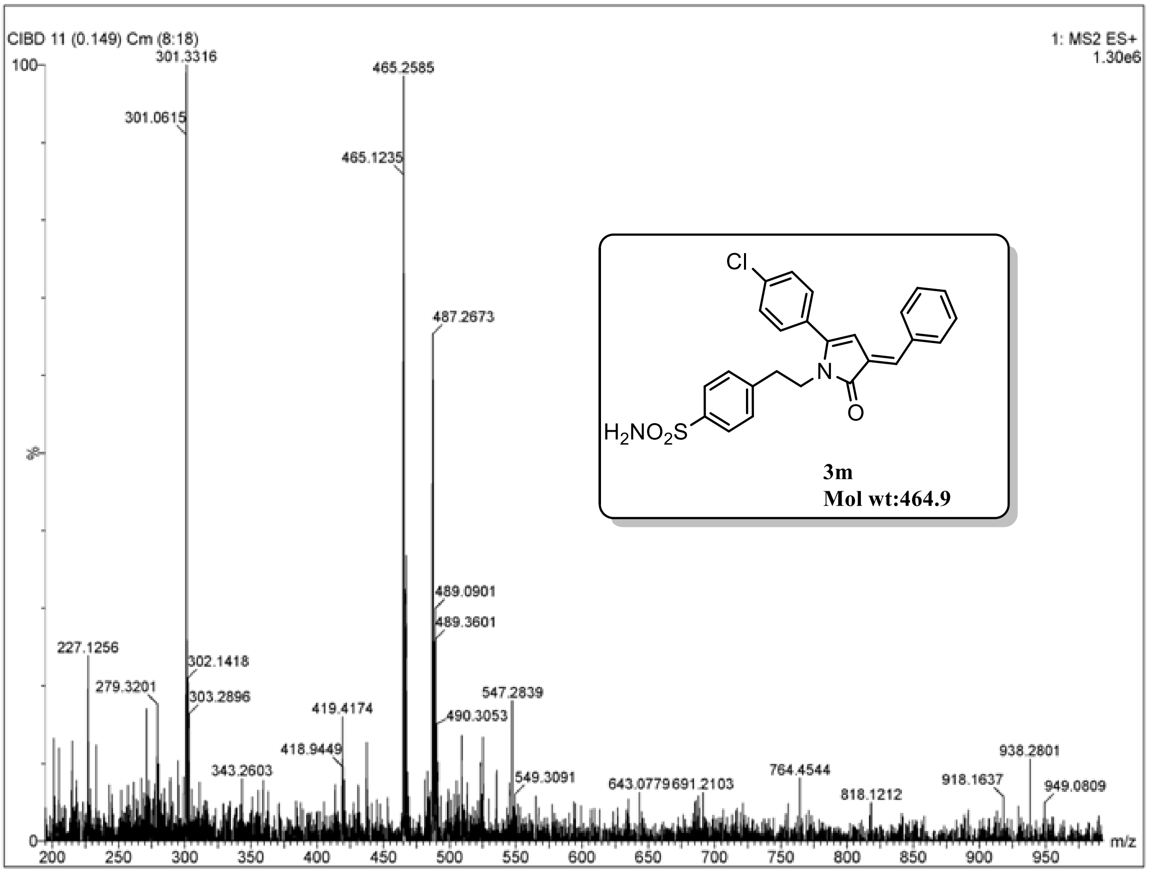
CIBD 11 (0.149) Cm (8:18)
1: MS2 ES+
1.30e6
100
%
0
301.3316
301.0615
465.2585
465.1235
487.2673
489.0901
489.3601
227.1256
302.1418
279.3201
303.2896
419.4174
547.2839
490.3053
418.9449
343.2603
549.3091
643.0779
691.2103
764.4544
818.1212
918.1637
938.2801
949.0809
200
250
300
350
400
450
500
550
600
650
700
750
800
850
900
950
m/z
Cl
N
O
H2NO2S
3m
Mol wt:464.9

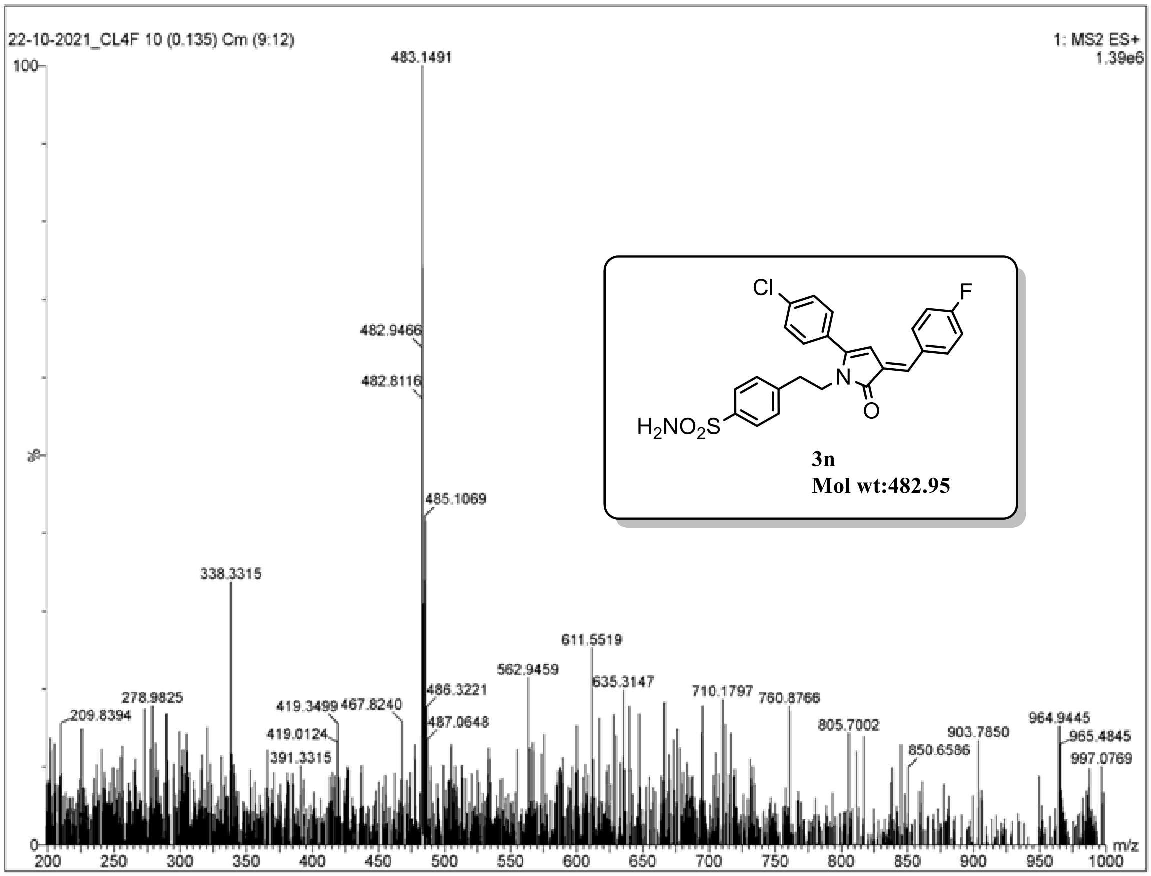

22-10-2021_CL4F 10 (0.135) Cm (9:12)
1: MS2 ES+
1.39e6
483.1491
482.9466
482.8116
485.1069
338.3315
611.5519
562.9459
635.3147
486.3221
710.1797
760.8766
278.9825
209.8394
419.3499
467.8240
487.0648
805.7002
903.7850
964.9445
965.4845
419.0124
391.3315
850.6586
997.0769
Cl
F
N
O
H2NO2S
3n
Mol wt:482.95
%
m/z

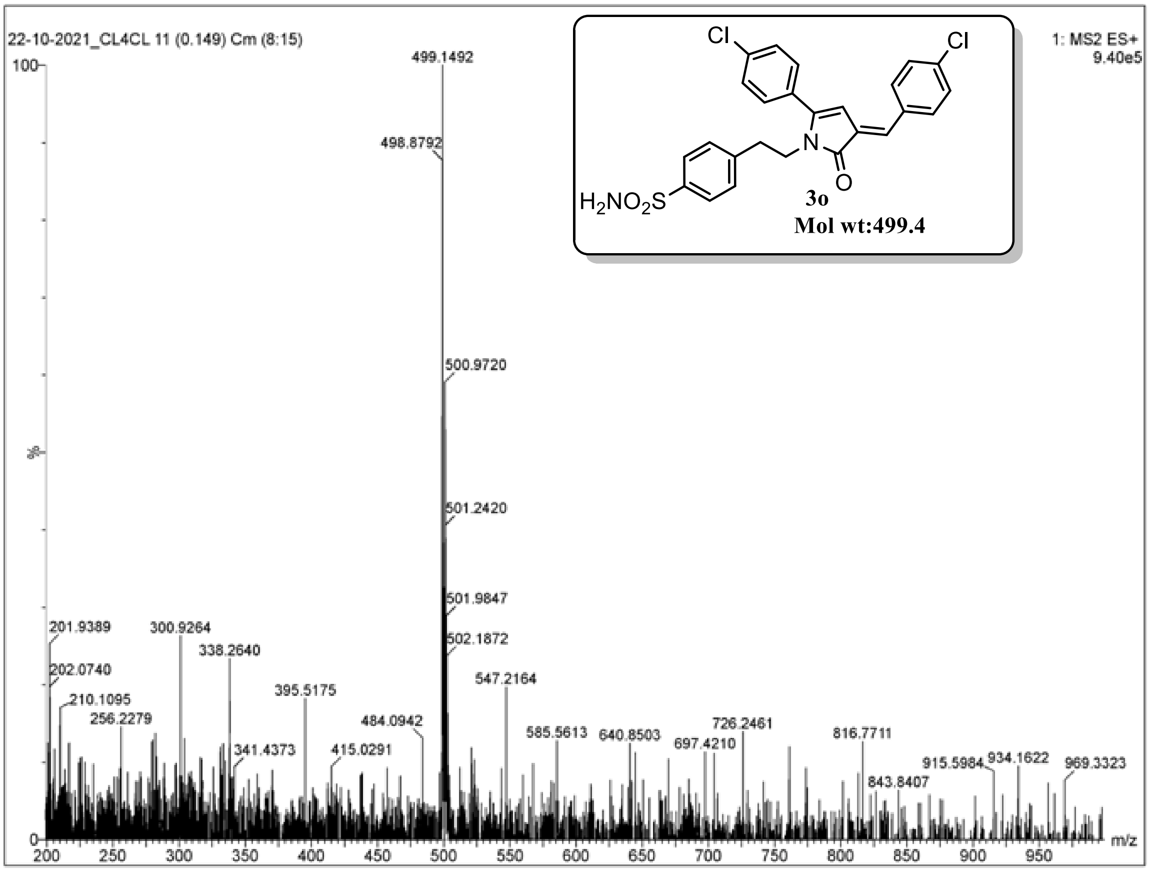

22-10-2021_CL4CL 11 (0.149) Cm (8:15)
1: MS2 ES+
9.40e5
499.1492
498.8792
500.9720
501.2420
501.9847
502.1872
201.9389
202.0740
210.1095
256.2279
300.9264
338.2640
341.4373
395.5175
415.0291
484.0942
547.2164
585.5613
640.8503
697.4210
726.2461
816.7711
843.8407
915.5984
934.1622
969.3323
%
m/z
Cl
Cl
N
O
H_2NO_2S
3o
Mol wt:499.4

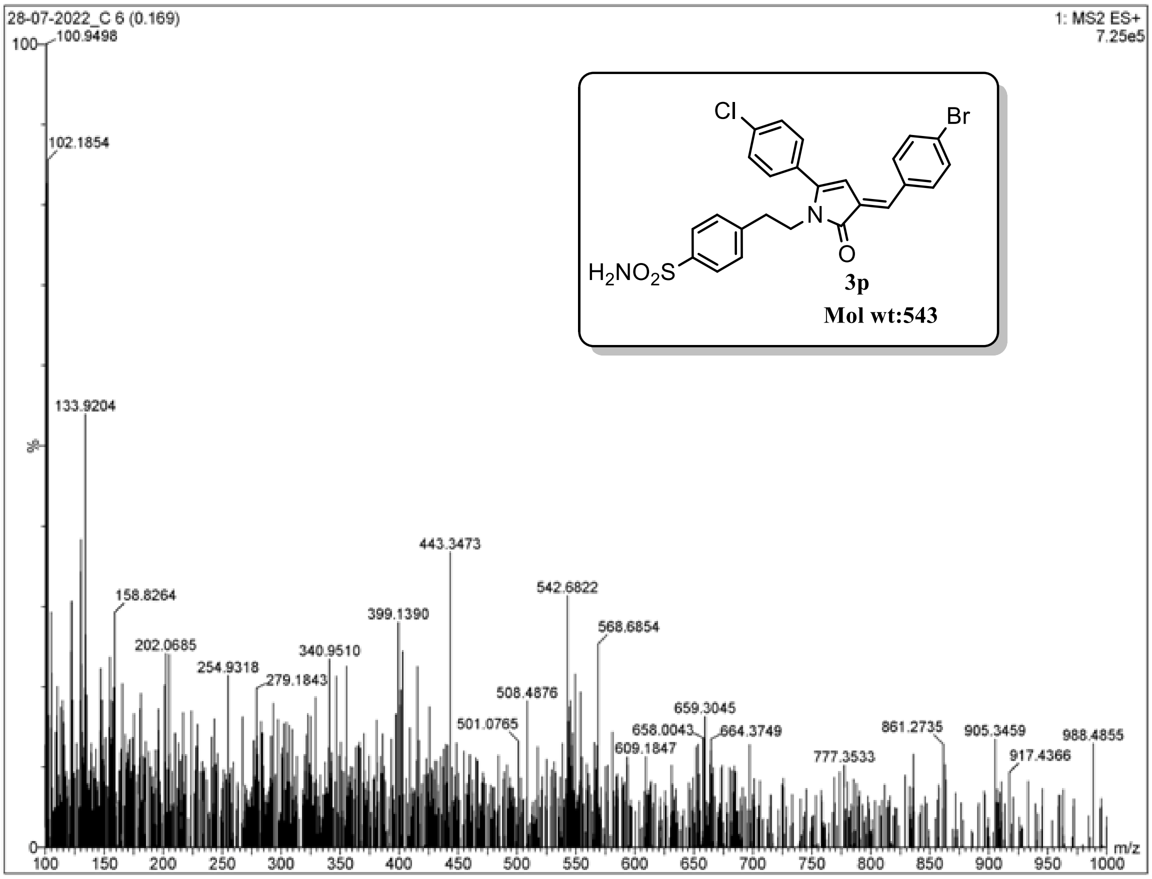
28-07-2022_C 6 (0.169)
1: MS2 ES+
7.25e5
100.9498
102.1854
133.9204
158.8264
202.0685
254.9318
279.1843
340.9510
399.1390
443.3473
501.0765
508.4876
542.6822
568.6854
609.1847
658.0043
659.3045
664.3749
777.3533
861.2735
905.3459
917.4366
988.4855
%
m/z
Cl
Br
N
O
H_2NO_2S
3p
Mol wt:543

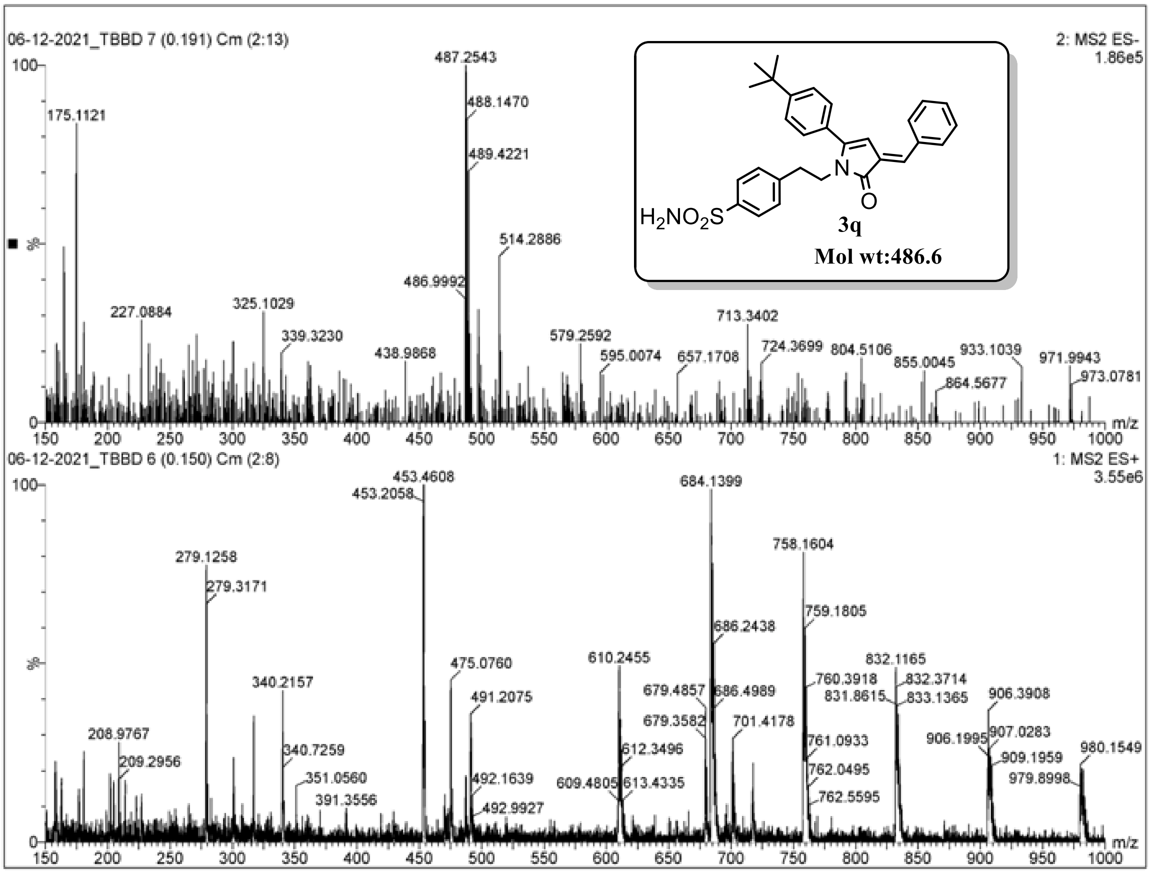

06-12-2021_TBBD 7 (0.191) Cm (2:13)
2: MS2 ES-
1.86e5
487.2543
488.1470
489.4221
175.1121
514.2886
486.9992
227.0884
325.1029
339.3230
438.9868
579.2592
595.0074
657.1708
713.3402
724.3699
804.5106
855.0045
864.5677
933.1039
971.9943
973.0781
H2NO2S
3q
Mol wt:486.6
06-12-2021_TBBD 6 (0.150) Cm (2:8)
1: MS2 ES+
3.55e6
453.4608
453.2058
684.1399
758.1604
279.1258
279.3171
759.1805
686.2438
610.2455
475.0760
340.2157
491.2075
832.1165
760.3918
832.3714
831.8615
833.1365
679.4857
686.4989
906.3908
679.3582
701.4178
208.9767
209.2956
340.7259
761.0933
906.1995
907.0283
909.1959
980.1549
612.3496
492.1639
351.0560
609.4805
613.4335
762.0495
979.8998
391.3556
762.5595
492.9927
m/z
%

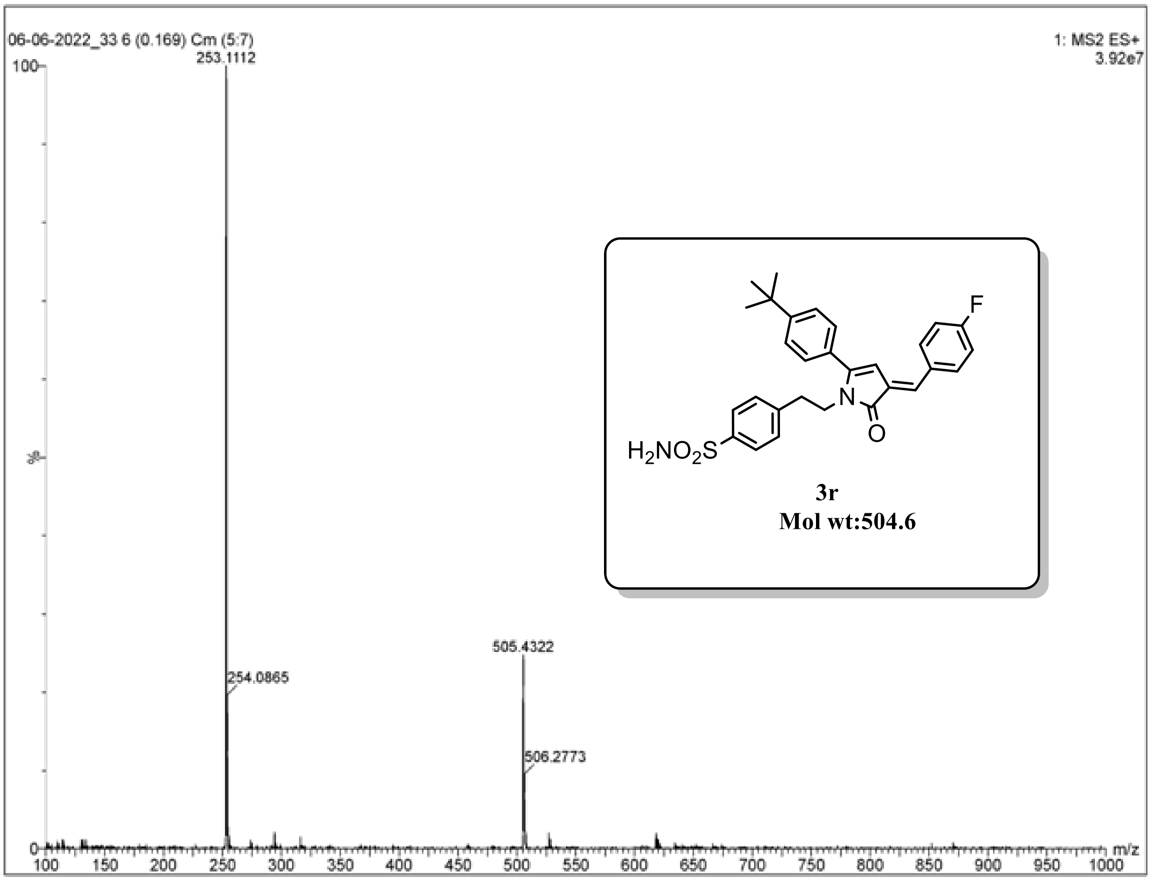
06-06-2022_33 6 (0.169) Cm (5:7)
1: MS2 ES+
3.92e7
100
%
0
253.1112
254.0865
505.4322
506.2773
m/z
100 150 200 250 300 350 400 450 500 550 600 650 700 750 800 850 900 950 1000
F
N
O
H_2NO_2S
3r
Mol wt:504.6

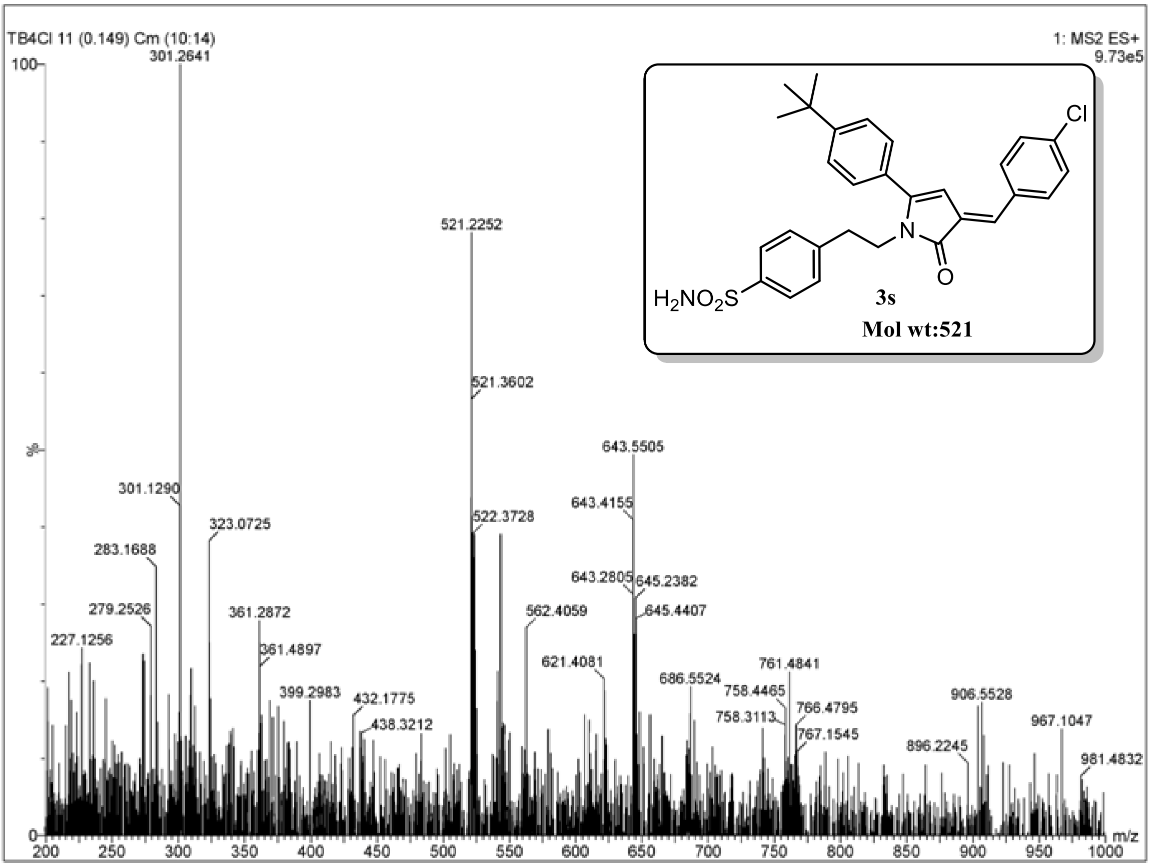

TB4Cl 11 (0.149) Cm (10:14)
1: MS2 ES+
9.73e5
100
%
0
301.2641
521.2252
521.3602
643.5505
643.4155
301.1290
323.0725
522.3728
283.1688
643.2805
645.2382
279.2526
361.2872
562.4059
645.4407
227.1256
361.4897
621.4081
761.4841
686.5524
399.2983
432.1775
758.4465
906.5528
766.4795
438.3212
758.3113
967.1047
767.1545
896.2245
981.4832
m/z
200
250
300
350
400
450
500
550
600
650
700
750
800
850
900
950
1000
Cl
N
O
H_2NO_2S
3s
Mol wt:521

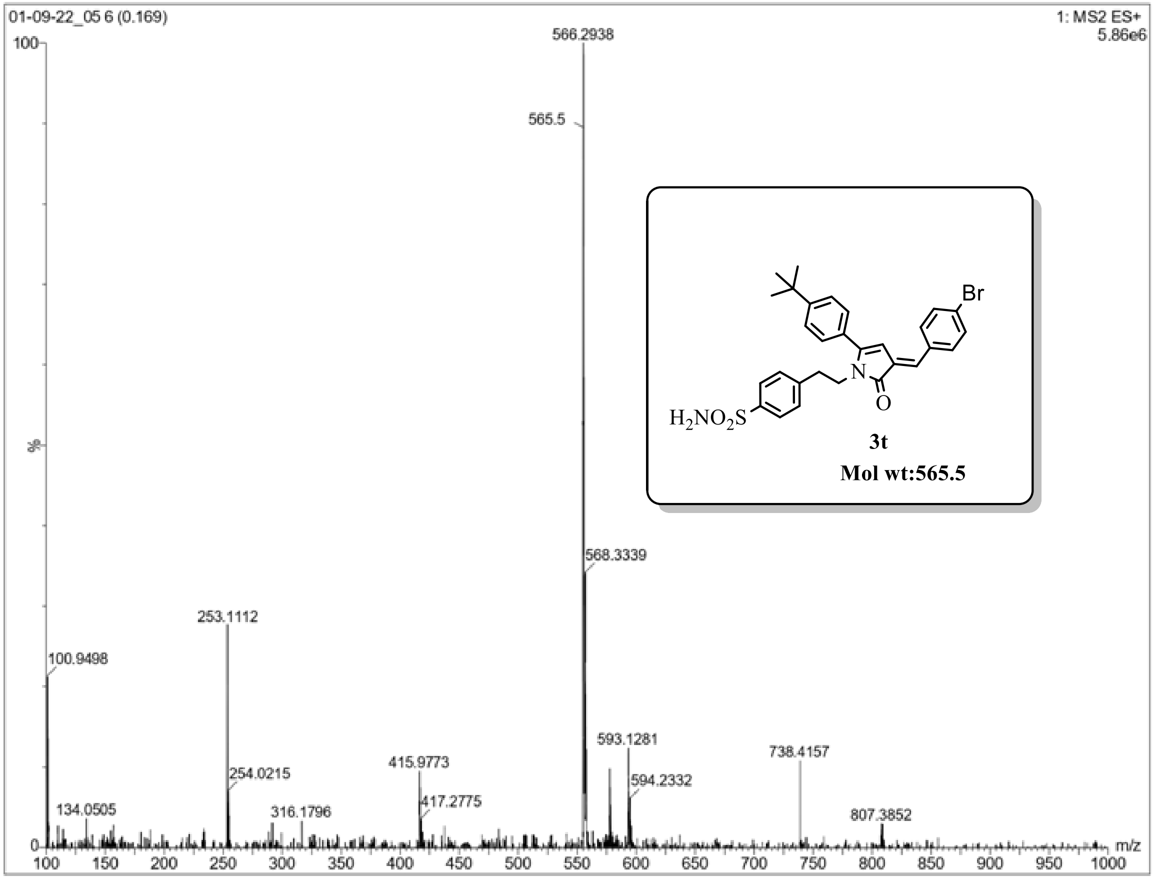

01-09-22_05 6 (0.169)
1: MS2 ES+
5.86e6
566.2938
565.5
568.3339
253.1112
100.9498
593.1281
594.2332
738.4157
415.9773
254.0215
417.2775
134.0505
316.1796
807.3852
%
m/z
Br
N
O
H2NO2S
3t
Mol wt:565.5

IR Spectra

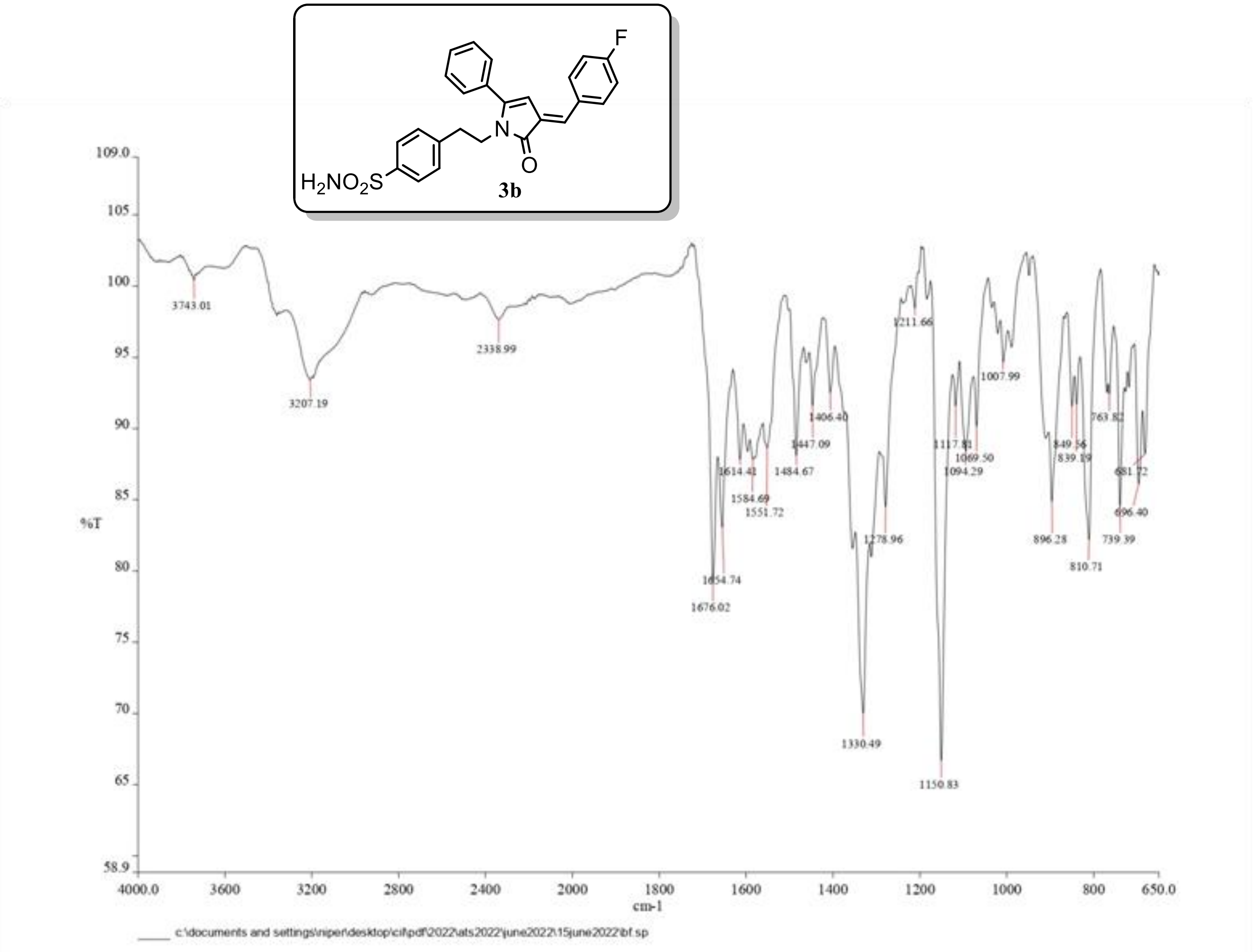

F
N
O
H_2NO_2S
3b
109.0
105
100
95
90
85
80
75
70
65
58.9
%T
3743.01
3207.19
2338.99
1676.02
1654.74
1614.41
1584.69
1551.72
1484.67
1447.09
1406.40
1330.49
1278.96
1211.66
1150.83
1117.81
1094.29
1069.50
1007.99
896.28
849.56
839.19
810.71
763.82
739.39
696.40
681.72
4000.0
3600
3200
2800
2400
2000
1800
1600
1400
1200
1000
800
650.0
cm-1
c:\documents and settings\niper\desktop\cif\pdf\2022\ats2022\june2022\15june2022\bf.sp

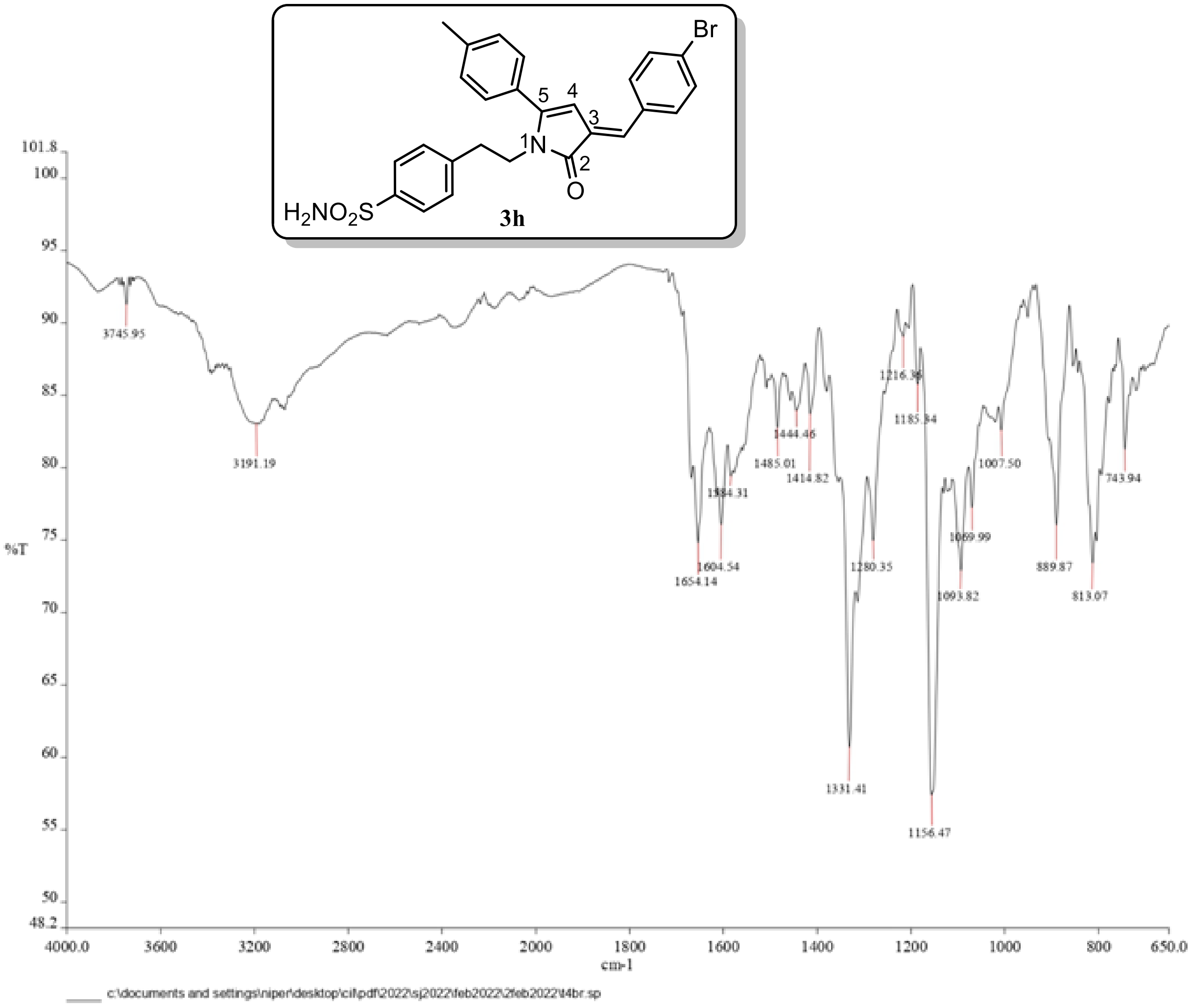

Br
5
4
3
1N
2
O
H2NO2S
3h
%T
cm-1
3745.95
3191.19
1654.14
1604.54
1485.01
1444.46
1414.82
1331.41
1280.35
1216.36
1185.34
1156.47
1093.82
1069.99
1007.50
889.87
813.07
743.94
c:\documents and settings\niper\desktop\cil\pdf\2022\sj2022\feb2022\2feb2022\t4br.sp

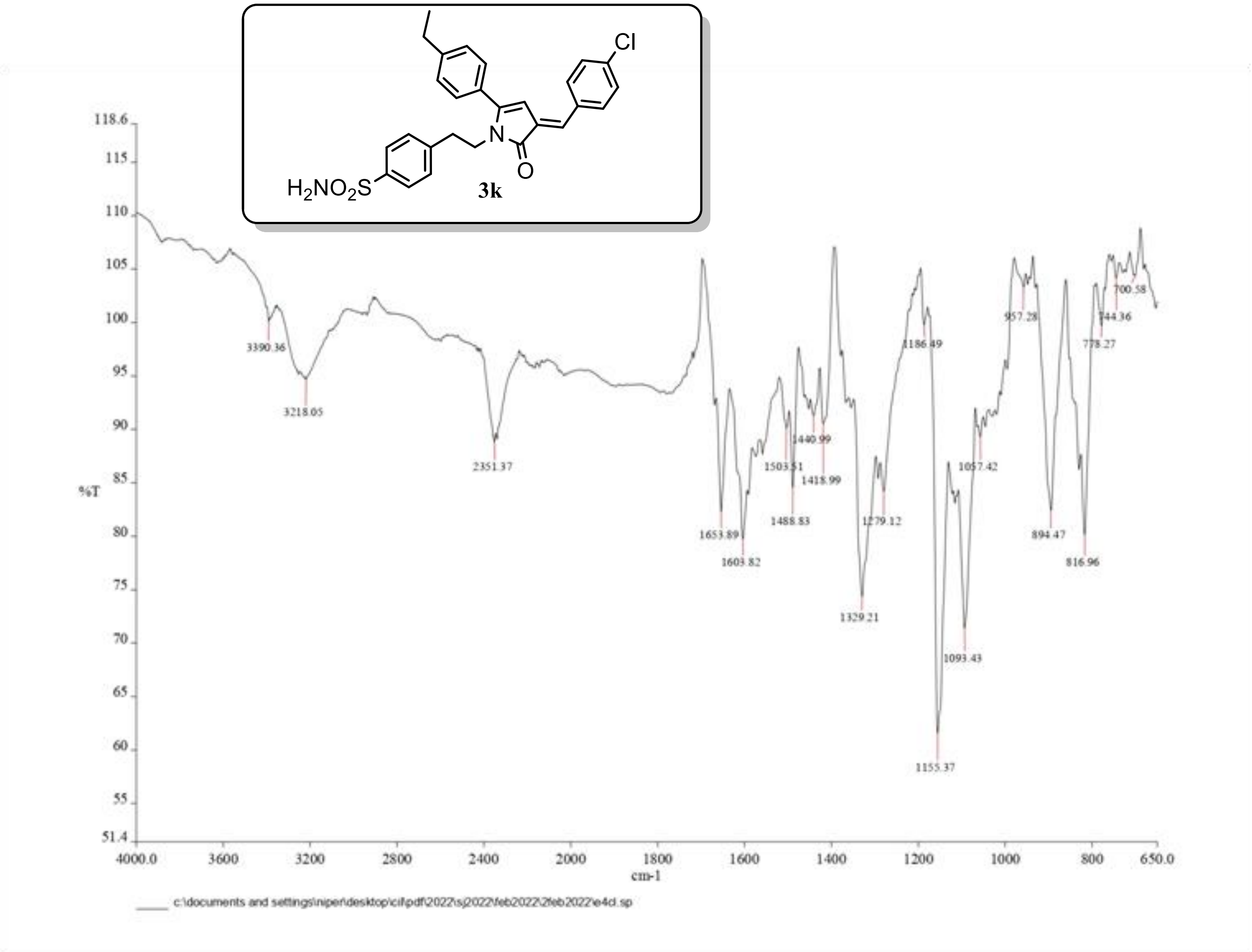
Cl
H_2NO_2S
N
O
3k
118.6
115
110
105
100
95
90
85
80
75
70
65
60
55
51.4
%T
4000.0
3600
3200
2800
2400
2000
1800
1600
1400
1200
1000
800
650.0
cm-1
3390.36
3218.05
2351.37
1653.89
1603.82
1503.51
1488.83
1440.99
1418.99
1329.21
1279.12
1186.49
1155.37
1093.43
1057.42
957.28
894.47
816.96
778.27
744.36
700.58
c:\documents and settings\niper\desktop\cif\pdf\2022\sj2022\feb2022\2feb2022\e4cl.sp

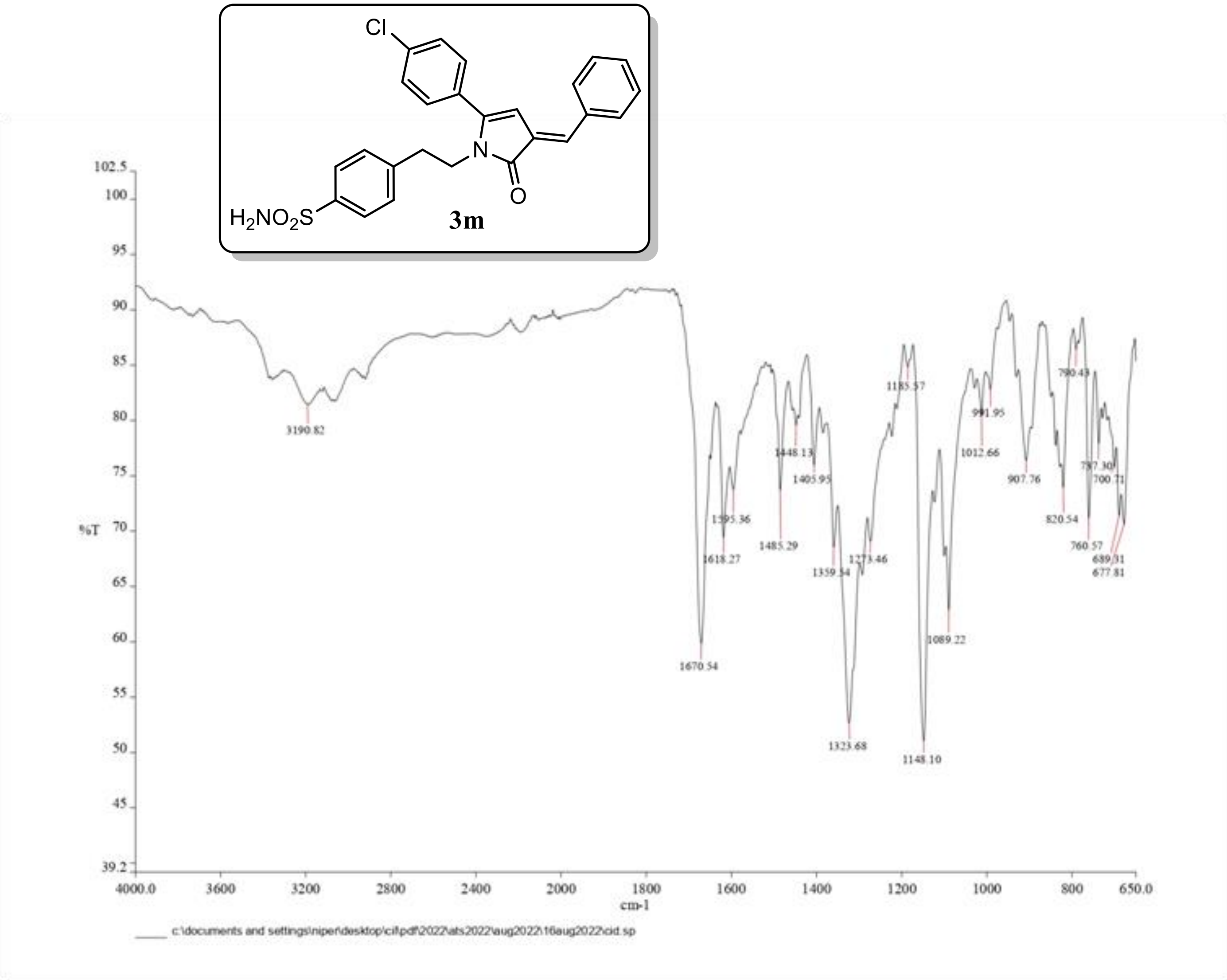
Cl
H_2NO_2S
N
O
3m
102.5
100
95
90
85
80
75
70
65
60
55
50
45
39.2
%T
3190.82
1670.54
1618.27
1595.36
1485.29
1448.13
1405.95
1359.54
1323.68
1273.46
1185.57
1148.10
1089.22
1012.66
991.95
907.76
820.54
790.43
760.57
737.30
700.71
689.31
677.81
4000.0
3600
3200
2800
2400
2000
1800
1600
1400
1200
1000
800
650.0
cm-1
c:\documents and settings\niper\desktop\cif\pdf\2022\ats2022\aug2022\16aug2022\cid.sp

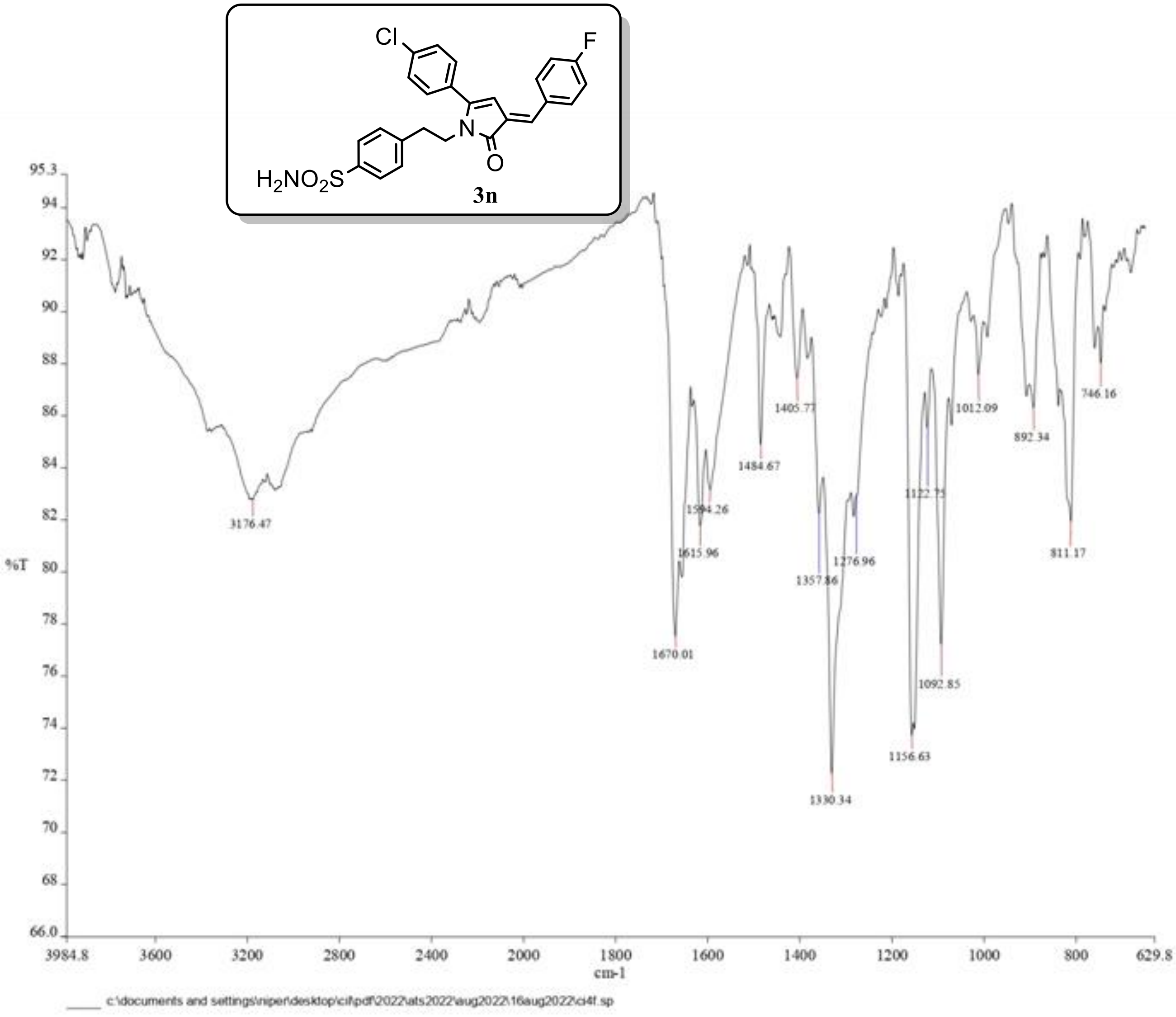
Cl
F
N
O
H2NO2S
3n
95.3
94
92
90
88
86
84
82
80
78
76
74
72
70
68
66.0
%T
3984.8
3600
3200
2800
2400
2000
1800
1600
1400
1200
1000
800
629.8
cm-1
3176.47
1670.01
1615.96
1594.26
1484.67
1405.77
1357.86
1330.34
1276.96
1156.63
1122.75
1092.85
1012.09
892.34
811.17
746.16
c:\documents and settings\niper\desktop\cif\pdf\2022\ats2022\aug2022\16aug2022\ci4f.sp

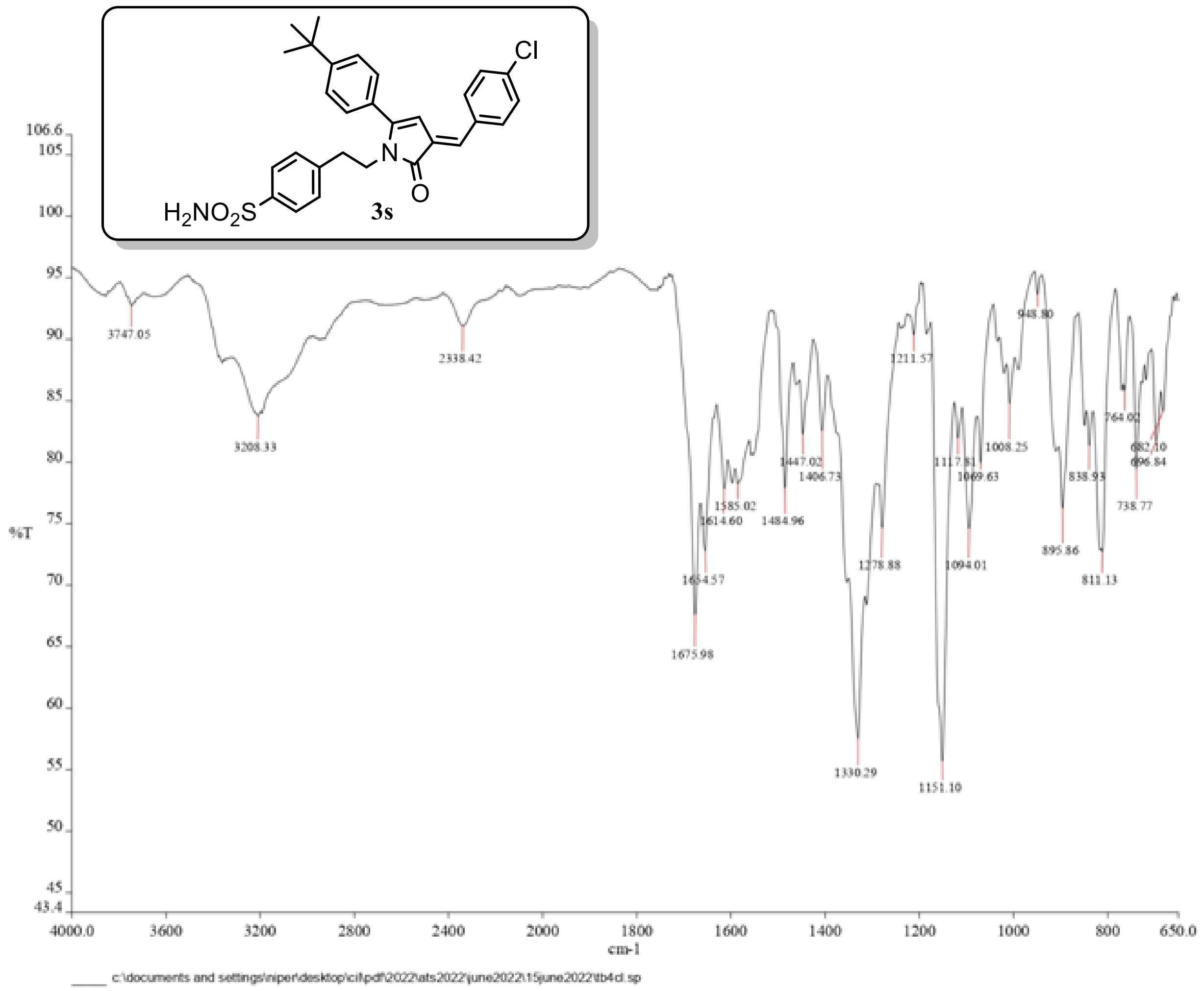

DFT Study

Table SX. Optimized Coordinates of 3n-E-isomer B3LYP/def2-TZVP D3BJ level of theory.

C	-1.567511	-1.129850	-0.368567
C	-0.212971	-1.115495	-0.022750
C	0.465101	0.100243	-0.014004
C	-0.196000	1.300552	-0.335310
C	-1.558865	1.256396	-0.658355

C	-2.252172	0.043813	-0.680711
H	0.293929	-2.047283	0.233653
H	1.527635	0.124652	0.245396
H	-2.084108	2.184311	-0.899533
H	-3.313294	-0.004846	-0.929746
S	-2.449209	-2.684915	-0.380090
O	-3.861213	-2.389973	-0.637256
O	-2.019720	-3.472863	0.776814
N	-1.782676	-3.471690	-1.721784
H	-2.387591	-3.368876	-2.537006
H	-1.645364	-4.454623	-1.480997
C	0.551898	2.606773	-0.273527
C	0.669649	3.131111	1.171110
H	0.057158	3.371865	-0.891939
H	1.565832	2.464015	-0.679002
H	-0.309551	3.471444	1.535824
H	1.005563	2.320673	1.832537
N	1.561855	4.269025	1.278051
C	1.077769	5.583915	1.279734
C	2.939707	4.275600	0.994887
C	2.276943	6.458998	1.037505
C	3.394288	5.556343	0.840643
H	4.441584	5.813992	0.717193
O	-0.087962	5.896860	1.419848
C	3.719713	3.030777	0.938445

C	4.589288	2.790157	-0.140621
C	3.624163	2.059093	1.952067
C	5.328663	1.610297	-0.219337
H	4.666142	3.530532	-0.939691
C	4.359528	0.877260	1.885445
H	2.986479	2.242398	2.819024
C	5.204978	0.654597	0.793348
H	5.991798	1.420866	-1.064910
H	4.287398	0.130104	2.677125
C	2.134963	7.811260	1.089405
Cl	6.114466	-0.830920	0.696951
H	1.123820	8.145137	1.349266
C	3.117744	8.863740	0.886539
C	2.809084	10.166810	1.335761
C	4.367127	8.663060	0.256909
C	3.712512	11.217952	1.201923
H	1.840930	10.347952	1.809279
C	5.275267	9.706958	0.108047
H	4.618963	7.685292	-0.150861
C	4.941282	10.971976	0.592194
H	3.481210	12.222998	1.559116
H	6.239162	9.561170	-0.383602
F	5.817704	11.974028	0.455899

Table SY. Optimized Coordinates of 3n-Z-isomer @B3LYP/def2-TZVP D3BJ level of theory.

C	-1.568285	-1.475774	-0.706087
C	-0.177593	-1.382859	-0.596109
C	0.418679	-0.127531	-0.668217
C	-0.355545	1.035464	-0.836580
C	-1.747839	0.910315	-0.935963
C	-2.362599	-0.342967	-0.874072
H	0.418872	-2.284521	-0.446981
H	1.505557	-0.042757	-0.580346
H	-2.360045	1.807834	-1.055726
H	-3.446243	-0.452394	-0.939067
S	-2.345202	-3.079491	-0.568407
O	-3.794446	-2.866963	-0.528901
O	-1.642328	-3.842296	0.465073
N	-1.926497	-3.823425	-2.029718
H	-2.700768	-3.773085	-2.692541
H	-1.665074	-4.790602	-1.832926
C	0.302934	2.391507	-0.817852
C	0.668596	2.792696	0.621579
H	-0.369841	3.157124	-1.229632
H	1.212666	2.382720	-1.439717
H	-0.259480	2.842736	1.212420
H	1.313532	2.031967	1.081431
N	1.333220	4.079190	0.719327
C	0.586542	5.255250	0.719133

C	2.653729	4.334753	1.148411
C	1.531602	6.338771	1.154675
C	2.791558	5.665161	1.415150
H	3.719934	6.155865	1.700310
O	-0.604836	5.279227	0.442682
C	3.683494	3.292993	1.224178
C	3.833640	2.316567	0.220204
C	4.587112	3.280605	2.303123
C	4.845169	1.360816	0.289376
H	3.170838	2.321731	-0.645924
C	5.603646	2.330560	2.382763
H	4.473919	4.018077	3.100480
C	5.726862	1.369968	1.374642
H	4.962828	0.614309	-0.497739
H	6.294981	2.322029	3.226868
C	1.343842	7.686197	1.303925
H	2.244132	8.208163	1.650438
C	0.222133	8.586590	1.105487
C	0.450578	9.959304	1.370696
C	-1.069509	8.201368	0.672217
C	-0.549695	10.911393	1.211445
H	1.439603	10.279298	1.708727
C	-2.075598	9.148931	0.508926
H	-1.268477	7.148922	0.471411
C	-1.806547	10.490544	0.778050

H	-0.376886	11.969656	1.415155
H	-3.074766	8.861935	0.175272
Cl	6.989894	0.170631	1.471054
F	-2.776237	11.398108	0.618533
